# The Two Tomato Ubiquitin E1 Enzymes Play Unequal Roles in Host Immunity

**DOI:** 10.1111/mpp.70160

**Published:** 2025-09-29

**Authors:** Chaofeng Wang, Bangjun Zhou, Xuanyang Chen, Lirong Zeng

**Affiliations:** ^1^ Center for Plant Science Innovation and Department of Plant Pathology University of Nebraska Lincoln Nebraska USA; ^2^ Biology Department University of Arkansas Little Rock Arkansas USA

**Keywords:** dual ubiquitin‐activating systems (DUAS), E2 charging, *Pseudomonas syringae* pv. *tomato*, tomato ( *Solanum lycopersicum* ), ubiquitin‐activating enzyme (E1), ubiquitin‐conjugating enzyme (E2)

## Abstract

Plants typically encode multiple ubiquitin‐activating enzymes (E1s or UBAs), but their functional equivalence or divergence remains unclear. Here, we demonstrate that the two tomato ( *Solanum lycopersicum* ) E1s, SlUBA1 and SlUBA2, differentially regulate development and immunity. Knockdown of *SlUBA1* or *SlUBA2* caused distinct growth and developmental defects in tomato, while silencing both genes resulted in severe abnormalities, rapid etiolation, and plant death within 5–7 weeks. Notably, silencing *SlUBA2*, but not *SlUBA1*, compromised plant immunity against the bacterial pathogen *Pseudomonas syringae* pv. *tomato* (Pst). SlUBA1 and SlUBA2 exhibited distinct charging efficiencies for E2s from groups IV (SlUBC32/33/34), V (SlUBC7/14/35/36), VI (SlUBC4/5/6/15) and XII (SlUBC22), with SlUBA2 showing significantly higher efficiency. Swapping the C‐terminal ubiquitin‐folding domains (UFDs) between SlUBA1 and SlUBA2 largely reversed their E2‐charging efficiency for these groups. Furthermore, mutating a key residue (SlUBA2^Q1009^) in the UFD or deleting a conserved 13‐amino‐acid sequence unique to group V E2s altered the E2‐charging profiles of both E1s. These findings suggest dual ubiquitin‐activating systems (DUAS) operate in tomato. Given the established role of group IV E2s in plant immunity against Pst, the SlUBA2‐group IV E2 module likely plays a central role in modulating host defence. Similarly, the *Arabidopsis* E1s, AtUBA1 and AtUBA2, differentially charge homologues of tomato group IV E2s, suggesting a conserved mechanism by which plant E1s fulfil distinct physiological roles.

## Graphical abstract

Tomato ubiquitin E1 enzymes SlUBA1 and SlUBA2 differentially regulate plant immunity by charging immunity‐associated E2 enzymes with distinct efficiencies.

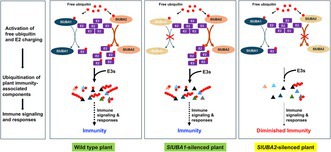

## 1 Introduction

Ubiquitination is a posttranslational protein modification (PTM) that plays key roles in numerous cellular and physiological processes, including host immunity against several kinds of pathogens (Zhang and Zeng 2020; Zhou et al. 2017). The stepwise enzymatic cascade catalysing ubiquitination typically consists of three different classes of enzymes, ubiquitin‐activating enzyme (E1 or UBA), ubiquitin‐conjugating enzyme (E2 or UBC) and ubiquitin ligase (E3) (Callis 2014). In the E1‐E2‐E3 cascade, E1 stands at the apex; thus, the modification of proteins by ubiquitin depends on the abundance, activity, and specificity of the E1 enzymes (Schulman and Harper 2009). The E1 enzyme initiates the ubiquitination process by coordinating two intricately connected reactions. The first reaction activates a free ubiquitin molecule by coupling ATP hydrolysis with the formation of a high‐energy E1 ~ ubiquitin thioester linkage between an E1 catalytic cysteine residue and the C terminus of ubiquitin (Haas et al. 1982). The second reaction recruits the cognate E2 enzyme and transfers the activated ubiquitin from the E1 to the E2 catalytic cysteine in a process called E1‐E2 thioester transfer (Hershko et al. 1983). Integration of the two reactions by an E1 enzyme leads to the attachment of an activated ubiquitin molecule to the cognate E2 enzyme with fidelity, a process also termed as E2 charging. Charged E2 ~ ubiquitin then interacts with various E3 ligases that facilitate the conjugation of one or more ubiquitin molecules to specific substrates (Ye and Rape 2009).

The importance of E1 enzymes has been investigated in various organisms and cell lines. Classic experiments using rodent temperature‐sensitive UBE1 mutant cell lines revealed that blocking the function of UBE1 resulted in disruption of ubiquitin conjugation, reduced protein turnover, and cell cycle arrest (Finley et al. 1984; Ciechanover et al. 1984). Studies on yeast indicated that the E1 enzyme UBA1 is indispensable for yeast sporulation and cell proliferation, and loss of E1 function results in cell death (Ghaboosi and Deshaies 2007; McGrath et al. 1991). In *Caenorhabditis elegans* , the E1 protein UBA‐1 plays multiple roles throughout the development of the worm, and the *uba‐1* knockout mutant is lethal (Kulkarni and Smith 2008). Phenotypic analysis of both weak and strong E1 (Uba1) mutant alleles of *Drosophila* demonstrated that impaired ubiquitin conjugation has significant consequences for the organism (Lee et al. 2008). Nevertheless, the weak and strong *Uba1* mutant alleles behave genetically differently, with the weak *Uba1* alleles protecting cells from cell death, whereas the strong *Uba1* alleles are highly apoptotic. Despite these discoveries, the precise mechanistic basis of Uba1 functioning in various processes is unclear.

An eukaryotic genome typically encodes dozens of different E2s and hundreds of different E3s (Vierstra 2009; Petroski and Deshaies 2005). By contrast, considerably fewer E1 enzymes exist. Humans possess two ubiquitin E1 activation systems that are directed by distantly related E1 enzymes UBE1 and UBA6 (Jin et al. 2007). The UBA6 and UBE1 display distinct preferences for E2 charging in vitro, with the E1‐E2 specificity depending partly on their C‐terminal UFD, which is similar to that of the yeast E1 (Lee and Schindelin 2008; Olsen and Lima 2013; Jin et al. 2007). The UBA6 orthologues were detected in vertebrates and the echinoderm sea urchin but not in insects, worms, fungi, and plants (Jin et al. 2007). To date, plant ubiquitin E1 enzymes have been isolated, with their ubiquitin‐activating activity being demonstrated, from wheat (Hatfield and Vierstra 1992), *Nicotiana tabacum* (Takizawa et al. 2005), *Arabidopsis* and soybean (Zhang et al. 2018). These studies and our data mining of various plant genomes reveal that most plant species encode two or more E1s that are homologues of human UBE1 (Table S1). However, no systematic studies of plant E1 enzymes have been reported and whether the E1s in a plant play equal or differential roles remains largely unknown. In *N. tabacum* , expression of the two E1 genes, *NtUBA1* and *NtUBA2*, is induced in response to viral infection, wounding, and defence‐related hormones, leading to the speculation that they might play equal roles in stress responses (Takizawa et al. 2005). In contrast, the two *Arabidopsis* E1 enzymes apparently function differentially in plant disease resistance, albeit the underlying mechanism remains to be elucidated (Goritschnig et al. 2007).

In this study, we reveal that tomato ( *Solanum lycopersicum* ) possesses dual E1 ubiquitin‐activating systems (DUAS) that are directed by two E1s, SlUBA1 and SlUBA2. The DUAS involve differential charging of four groups of E2s and play unequal roles in plant immunity and development. The C‐terminal UFD of the tomato E1s is shown to play a vital role in governing differential charging of the E2s. In addition, we reveal that the subtle difference in the E2 structure also contributes to the differential E2 charging by the two tomato E1s. Noteworthy, the two *Arabidopsis* E1s also differentially charge the *Arabidopsis* counterparts of tomato group IV E2s, AtUBC32, AtUBC33 and AtUBC34. The plant E2 enzymes UBC32, UBC33, and UBC34 were recently reported to be involved in endoplasmic reticulum (ER)‐associated protein degradation (ERAD) and play important roles in plant immunity against bacterial pathogen *Pseudomonas syringae* pv.*tomato* (Pst) (Wang et al. 2025). These results suggest that differential charging of relevant E2s contributes to the unequal roles played by the DUAS in plant immunity.

## 2 Results

### 2.1 Tomato and *Nicotiana benthamiana* Genomes Encode Two and Four Ubiquitin E1s, Respectively

**FIGURE 1:**
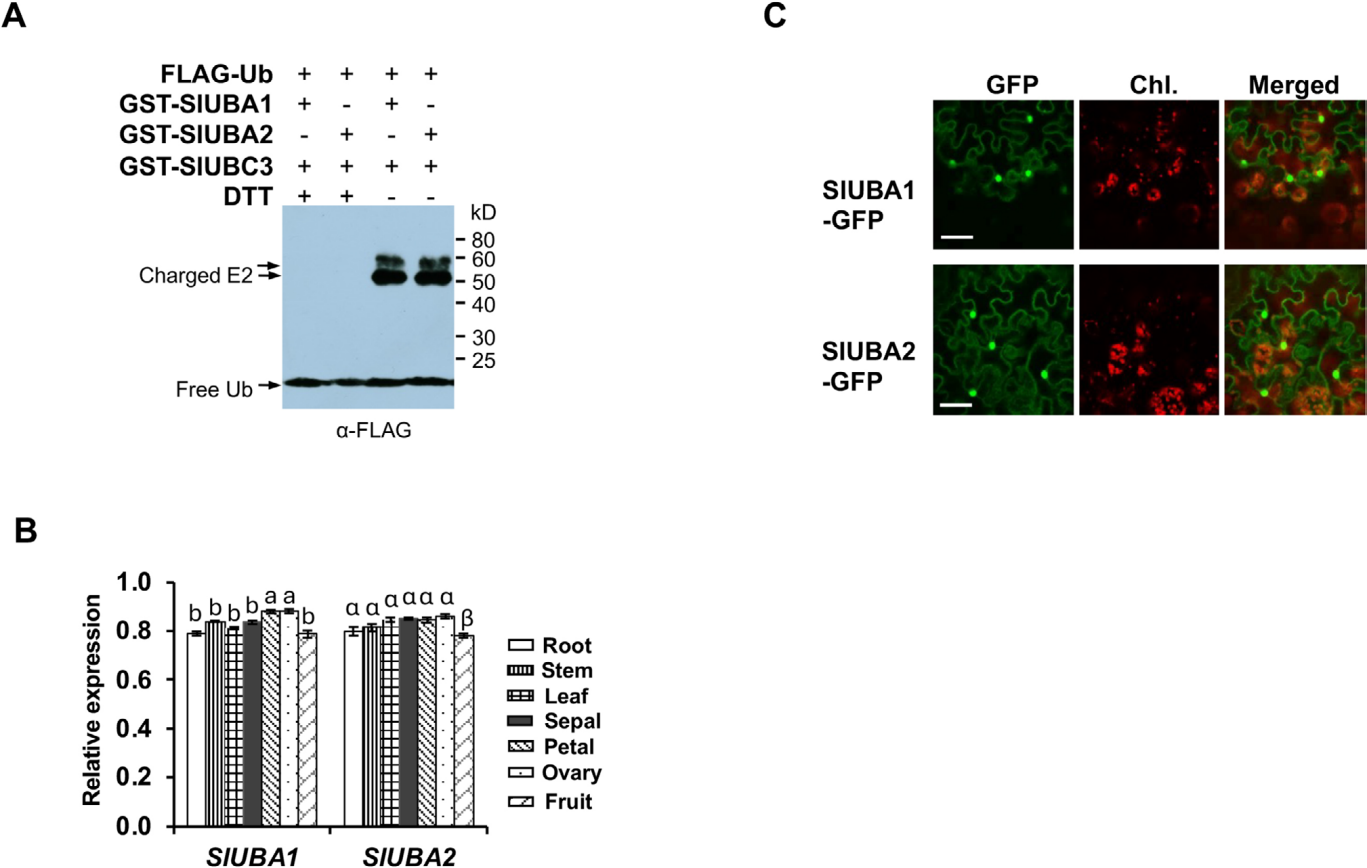
The tomato genome encodes two active ubiquitin E1s that show comparable expression in various tissues and similar subcellular localisation. (A) Thioester assay of SlUBC3 shows the tomato SlUBA1 and SlUBA2 are active E1 enzymes. The numbers on the right denote the molecular mass of marker proteins in kDa. (B) The *SlUBA1* and *SlUBA2* genes show comparable levels of expression across all tomato tissues tested. *SlUBA1* showed significantly higher expression in sepals and ovaries, whereas *SlUBA2* displayed significantly lower expression in fruits relative to its expression in other tissues. The expression of the E1 genes was examined by reverse transcription‐quantitative PCR using three biological replicates, with three technical replicates for each biological replicate. The expression levels of the E1 genes were analysed using Tukey–Kramer HSD test. Significant differences are marked with different lowercase letters (*p* = 0.05). (C) SlUBA1 and SlUBA2 are present in both cytoplasm and nucleus. GFP‐fused SlUBA1 and SlUBA2 in *Nicotiana benthamiana* leaves was examined by confocal microscopy. White bar marks a scale of 20 μm.

The E1 ubiquitin‐activating enzyme exhibits a characteristic architecture comprising three conserved domains: a pseudo‐dimeric adenylation domain responsible for ubiquitin activation, a Cys domain containing the catalytic cysteine residue, and a ubiquitin‐fold domain (UFD) that facilitates E2 enzyme recruitment (Schäfer et al. 2014; Olsen and Lima 2013; Lee and Schindelin 2008). Using the sequences of ubiquitin E1 enzymes from *Arabidopsis* and wheat to query the tomato ( *Solanum lycopersicum* ) genome, we identified two genes—*Solyc06g007320* and *Solyc09g018450*—encoding proteins with all the hallmark E1 domains and an estimated molecular mass of approximately 110 kDa (Hatfield and Vierstra 1992; Hatfield et al. 1997; The Tomato Genome Consortium 2012) (Figures S1 and S12A). These genes were designated *SlUBA1* ( *Solanum lycopersicum* ubiquitin‐activating enzyme1, *Solyc06g007320*) and *SlUBA2* (*Solyc09g018450*), respectively, based on the order of their cloning. In vitro thioester assays demonstrated that both SlUBA1 and SlUBA2 catalyse the formation of ubiquitin adducts with the tomato E2 enzyme SlUBC3, a reaction sensitive to dithiothreitol (DTT) (Figure 1A), confirming their ubiquitin‐activating activity. Like tomato, *N. benthamiana*, another solanaceous species widely used as a model for plant immunity studies, was analysed for E1 homologues. Searching the *N. benthamiana* genome (Wang et al. 2024) with *SlUBA1* and *SlUBA2* sequences revealed four genes—*Nbe03g13750.1*, *Nbe04g02160.1*, *Nbe14g09490.1* and *Nbe18g13930.1*—encoding proteins with all conserved E1 domains. These genes, named *NbUBA1a*, *NbUBA1b*, *NbUBA2a* and *NbUBA2b*, exhibit high DNA sequence identity with their tomato counterparts. *NbUBA1a* (*Nbe03g13750.1*) and *NbUBA1b* (*Nbe04g02160.1*) are 90.95% and 90.16% identical to *SlUBA1*, respectively, whereas *NbUBA2a* (*Nbe14g09490.1*) and *NbUBA2b* (*Nbe18g13930.1*) share 90.86% and 90.89% identity with *SlUBA2* (Figures S3 and S4). Consistent with prior findings (Jin et al. 2007), no homologues of human *UBA6* were detected in either tomato or *N. benthamiana*. Phylogenetic analysis confirmed that SlUBA1 and SlUBA2 proteins share the highest homology with NbUBA1a, and NbUBA1b, NbUBA2a and NbUBA2b, respectively (Figure S2).

Expression analysis revealed that both *SlUBA1* and *SlUBA2* are expressed across all tested tomato tissues (root, stem, leaf, sepal, petal, ovary and fruit), with no significant differences in expression levels between the two E1 genes in any tissue (Figure 1B). *SlUBA1* showed significantly higher expression in sepals and ovaries, whereas *SlUBA2* displayed significantly lower expression in fruits relative to its expression in other tissues (Figure 1B). Subcellular localisation studies further showed that SlUBA1 and SlUBA2 are present in both the nucleus and cytoplasm (Figure 1C), consistent with the ubiquitous role of ubiquitination throughout the cell.

### 2.2 SlUBA1 and SlUBA2 Play Differential Roles in Plant Development

The similarity in gene expression and subcellular localisation between the two tomato E1 ubiquitin‐activating enzymes, SlUBA1 and SlUBA2, led us to investigate whether their functions are also comparable. To explore this, we silenced *UBA1* or *UBA2* gene expression in tomato and *N. benthamiana* using virus‐induced gene silencing (VIGS). Based on DNA sequence alignments of the *UBA1* and *UBA2* genes from both species, we selected a fragment from *SlUBA1* that is highly conserved among tomato *SlUBA1* and *N. benthamiana NbUBA1a* and *NbUBA1b* genes for specific silencing of these orthologues. Similarly, a fragment from *SlUBA2*, nearly identical across tomato *SlUBA2* and *N. benthamiana NbUBA2a* and *NbUBA2b* genes, was chosen for their targeted silencing (Figure S5A,B). These fragments were combined to simultaneously silence both *UBA1* and *UBA2* genes in tomato and *N. benthamiana*. Sequence alignments revealed low homology between the DNA fragments used for silencing *UBA1* and *UBA2*, with no identical stretches exceeding 23 consecutive base pairs (Figure S6A,B), indicating a low risk of off‐target silencing. Reverse transcription‐quantitative PCR (RT‐qPCR) confirmed specific and efficient silencing of *UBA1* and *UBA2* genes in tomato and *N. benthamiana* plants infected with *TRV‐SlUBA1*, *TRV‐SlUBA2* or *TRV‐SlUBA1/2* constructs (Figure S7).

**FIGURE 2:**
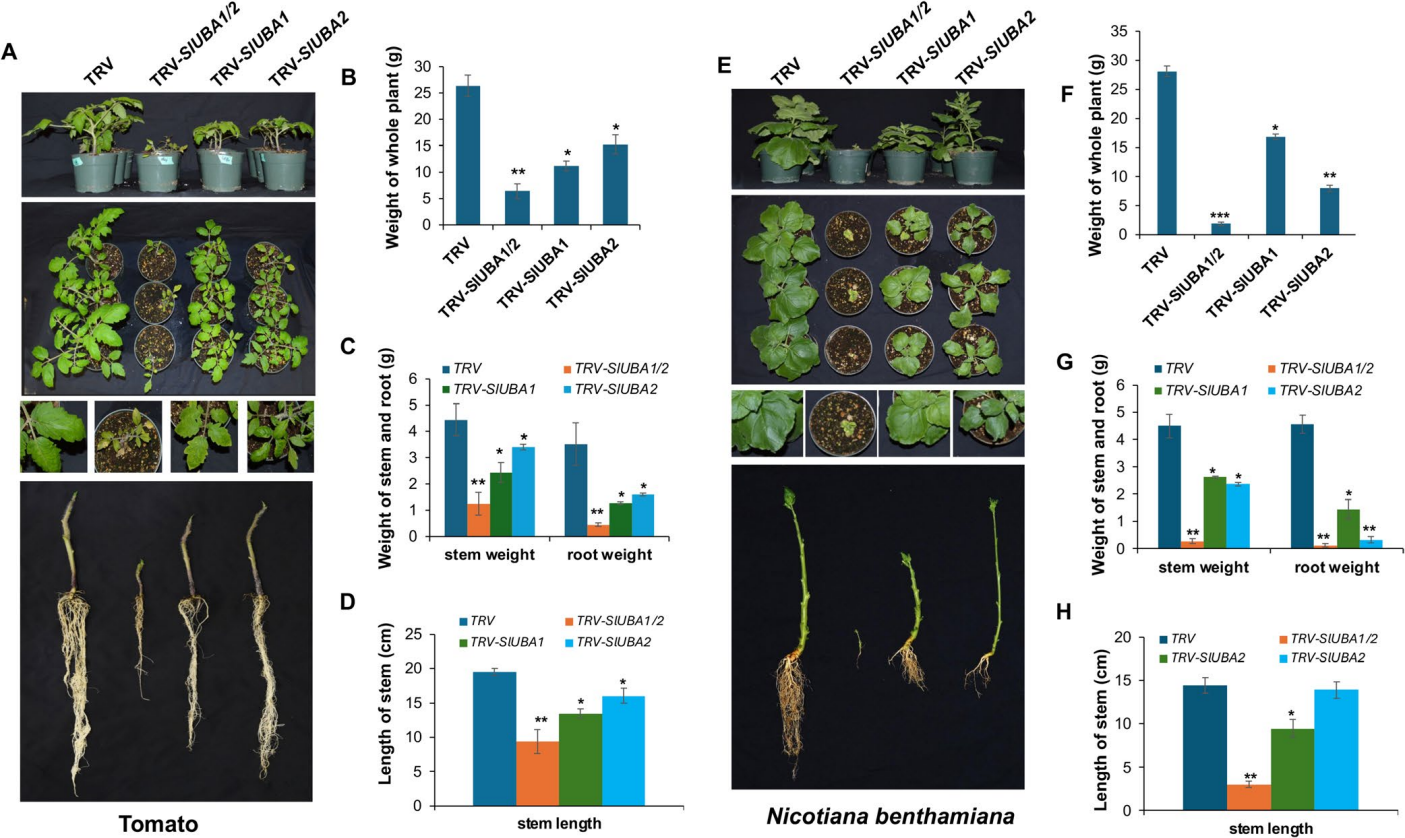
Differential roles of ubiquitin E1 genes in growth and development of tomato and *Nicotiana benthamiana*. (A–D) Tomato and (E–H) *N. benthamiana* plants with silenced *SlUBA1* or *NbUBA1a/1b* (TRV‐*SlUBA1*), *SlUBA2* or *NbUBA2a/2b* (TRV‐*SlUBA2*), or both (*SlUBA1/2*) were compared to TRV empty vector controls. (A, E) Photographs of approximately 7‐week‐old plants, taken ~4 weeks post‐virus‐induced gene silencing (VIGS) infiltration of ~3‐week‐old seedlings, showing: Top panels, side views; second panels, top views; third panels, close‐up leaf morphology; bottom panels, stems and roots. Plants with silenced *SlUBA1/2* or *NbUBA1/2* died 1–3 weeks after photography. (B–D, F–H) Quantification of (B, F) whole‐plant biomass, (C, G) stem and root biomass, and (D, H) stem length in silenced and control plants. Data represent means from at least four plants per treatment, analysed by one‐way ANOVA (*p < 0.05, **p < 0.01).

Knockdown of *UBA1* or *UBA2* in tomato ( *S. lycopersicum* ) and *N. benthamiana* resulted in distinct growth and developmental alterations. Plants silenced for either *SlUBA1/NbUBA1a/b* or *SlUBA2/NbUBA2a/b* genes exhibited reduced growth and altered development compared to controls (Figure 2A,E, Figure S8A,C). Specifically, *UBA1* (*SlUBA1* or *NbUBA1a/b*)‐silenced plants were dwarfed, with shorter internodes reduced taproot length, fewer lateral roots and slightly smaller leaves. In contrast, *UBA2*‐silenced plants maintained similar height to controls but displayed shorter taproots, significantly fewer lateral roots, and smaller, narrowly shaped leaves (Figure 2, Figure S8A,C). Quantification and statistical analyses revealed significantly reduced biomass in whole plants, stems and roots of *SlUBA1*‐, *SlUBA2*‐ and *SlUBA1/2*‐silenced plants compared to control plants transfected with the empty TRV2 vector (Figure 2B,C,F,G). Stem length was significantly reduced in *SlUBA1‐* and *SlUBA1/2‐*silenced tomato and *N. benthamiana* plants (Figure 2D,H), while *SlUBA2‐*silenced plants showed reduced stem length only in tomato (Figure 2D). Additionally, fully expanded tomato leaves and the third and fourth expanded leaves of *N. benthamiana* from *SlUBA1*‐ and *SlUBA2*‐silenced plants had significantly smaller areas than those of control plants (Figure S8B,D). These findings suggest distinct roles for UBA1 and UBA2 in modulating plant growth and development. Notably, plants silenced for both *UBA1* and *UBA2* genes exhibited severe growth defects, rapid etiolation, and death within 5–7 weeks post‐inoculation with TRV‐*UBA1/2*, underscoring functional redundancy between these E1 enzymes and the critical role of ubiquitination in plant development.

### 2.3 SlUBA1 and SlUBA2 Play Differential Roles in Host Immunity

**FIGURE 3:**
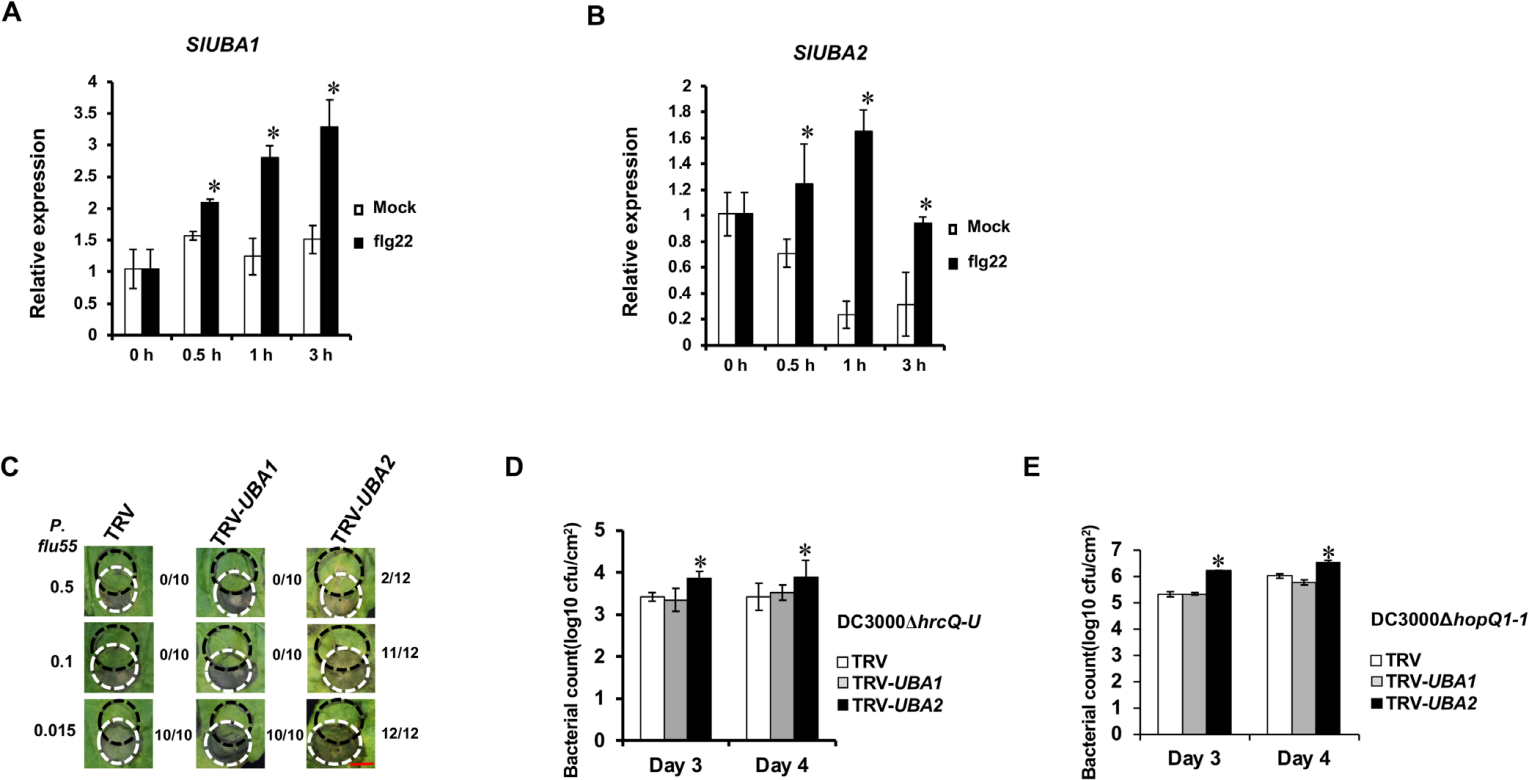
The two tomato E1s play differential roles in plant immunity. (A and B) The expression pattern of tomato E1 genes *SlUBA1* and *SlUBA2* after flg22 treatment. Reverse transcription‐quantitative real‐time PCR (RT‐qPCR) was used to measure the level of gene expression at indicated time points after flg22 infiltration, with three biological replicates and three technical replicates for each biological replicate being used. The mock (sterile water) infiltrated leaf tissues were used as control. Error bars indicate standard deviation. Asterisks indicate significantly elevated expression compared to mock plants at the same time point based on the one‐way ANOVA (*p* < 0.01). (C) Virus‐induced gene silencing (VIGS) of *UBA2* gene in *Nicotiana benthamiana* compromised PAMP‐triggered immunity (PTI)‐mediated cell death suppression. Black dashed circles denote the infiltration area of *Pseudomonas fluorescens* 55 (*P. flu55*) while white dashed circles denote infiltration area of *Pseudomonas syringae* pv. *tomato* (Pst) DC3000. The numbers on the left side indicate the corresponding concentration of *P. flu55* (OD_600_ value) used to activate PTI. The numbers on the right side of each image represent the number of overlapping infiltration areas that displayed cell death out of the total number of overlapping infiltration areas. Photographs were taken on Day 4 after infiltration of Pst DC3000. Red bar marks a scale of 1 cm. (D) and (E) Bacterial growth in *UBA1‐* or *UBA2*‐silenced tomato (D) and *N. benthamiana* (E) plants. Non‐silenced (TRV) plants served as control. Tomato VIGS plants (D) were vacuum infiltrated with Pst DC3000Δ*hrcQ‐U*. *N. benthamiana* VIGS plants (E) were vacuum infiltrated with *P. flu55* to induce PTI and then inoculated with Pst DC3000Δ*hopQ1–1* 6 h later. Asterisks indicate significantly elevated bacterial growth compared to the control plants based on the one‐way ANOVA (*p* < 0.01).

To determine whether SlUBA1 and SlUBA2 also contribute differently to host immunity, we first assessed their expression in tomato following treatment with flg22, a pathogen‐associated molecular pattern (PAMP). Both *SlUBA1* and *SlUBA2* genes were upregulated by flg22 (Figure 3A,B), suggesting their involvement in host immunity. We then evaluated PAMP‐triggered immunity (PTI) in *NbUBA1a/b*‐ and *NbUBA2a/b*‐silenced *N. benthamiana* plants using two assays. First, a cell death suppression assay (CDSA) was conducted on silenced and control plants (Chakravarthy et al. 2010). In this assay, PTI induced by the nonpathogenic *Pseudomonas fluorescens* 55 typically suppresses hypersensitive cell death caused by subsequent inoculation with *Pseudomonas syringae* pv. *tomato* (Pst) DC3000 in overlapping leaf areas (Figure 3C). However, cell death occurred in the overlapping regions of *NbUBA2a/b*‐silenced plants but not in *NbUBA1a/b*‐silenced plants, indicating compromised PTI in *UBA2*‐silenced *N. benthamiana* but not in *UBA1*‐silenced counterparts.

We further investigated the effects of *SlUBA1/NbUBA1a/b* and *SlUBA2/NbUBA2a/b* silencing on PTI by measuring the growth of two Pst strains, DC3000Δ*hrcQ‐U* and DC3000Δ*hopQ1*‐*1*, in tomato and *N. benthamiana*, respectively. The type III secretion system (T3SS)‐deficient Pst strain DC3000Δ*hrcQ‐U* elicits PTI in tomato (Kvitko et al. 2009). On *SlUBA2*‐silenced tomato plants, the growth of DC3000Δ*hrcQ‐U* was significantly higher at Days 3 and 4 post‐inoculation compared to *SlUBA1*‐silenced and TRV‐infected control plants (Figure 3D). Similarly, in *N. benthamiana*, the growth of Pst DC3000Δ*hopQ1*‐*1* was significantly increased in *NbUBA2a/b*‐silenced plants relative to *NbUBA1a/b*‐silenced and non‐silenced controls at Days 3 and 4 (Figure 3E). These results confirm that UBA1 and UBA2 contribute differentially to PTI, with UBA2 (SlUBA2 or NbUBA2a/b) playing a more prominent role in both tomato and *N. benthamiana*.

We subsequently selected tomato and *N. benthamiana* plants exhibiting varying levels of *SlUBA2/NbUBA2a/b* expression due to differences in gene silencing and divided them into two groups: Group 1, with stronger *SlUBA2* or *NbUBA2a/b* silencing, and Group 2, with weaker silencing. We evaluated their host immunity by measuring the growth of Pst DC3000Δ*hrcQ‐U* on tomato and DC3000Δ*hopQ1*‐*1* on *N. benthamiana* plants. As shown in Figure S9A,B, plants in Group 1, with greater *UBA2* silencing, exhibited slightly but statistically significantly higher pathogen growth compared to Group 2. Consistently, all *UBA2*‐silenced plants showed significantly higher pathogen growth than control plants. These results further support the connection between *UBA2* gene silencing and the observed immunity phenotypes in our assays.

### 2.4 Tomato SlUBA1 and SlUBA2 Differentially Charge Four Groups of E2s

**FIGURE 4:**
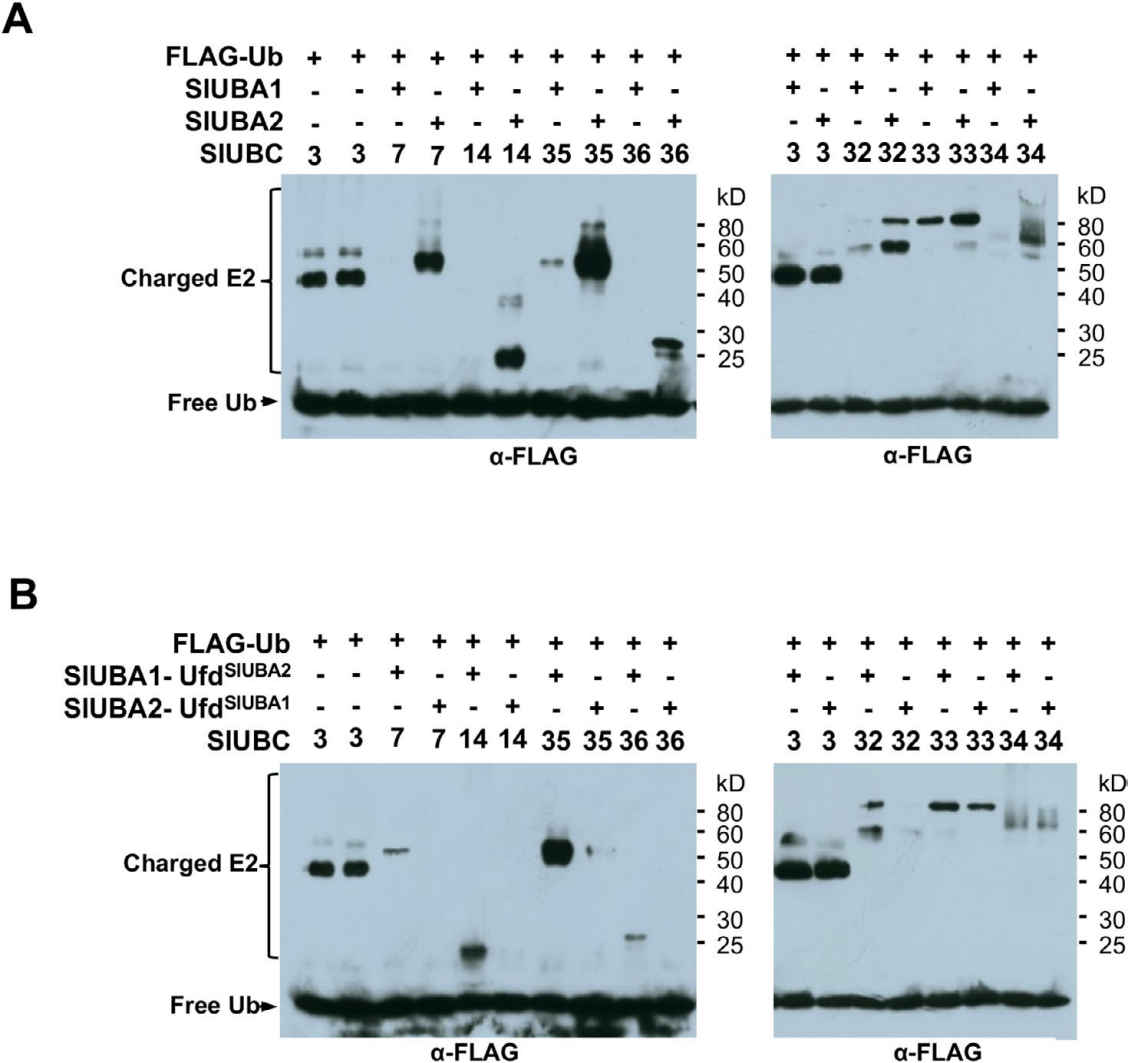
The two tomato E1s differentially charge a subset of ubiquitin E2s and the ubiquitin fold domain (UFD) of E1 plays a significant role in governing the specificity of E2 charging. (A) SlUBA1 and SlUBA2 differentially charge groups IV (SlUBC32, 33 and 34) and V (SlUBC7, 14, 35 and 36) E2s in thioester assay. SlUBC3 was used as control. (B) Chimeric E1s SlUBA1‐UFD^SlUBA2^ and SlUBA2‐UFD^SlUBA1^ reverse the specificities of SlUBA1 and SlUBA2 in charging group IV and V E2s. The numbers on the right denote the molecular mass of marker proteins in kilodaltons (kDa).

Ubiquitin‐activating enzymes (E1s) initiate the ubiquitination cascade by activating ubiquitin and transferring it to cognate E2 conjugating enzymes, a process known as E2 charging. To elucidate the molecular basis for the distinct roles of tomato E1s in plant development and host immunity, we assessed the charging efficiency of SlUBA1 and SlUBA2 using thioester assays across a panel of 34 E2s identified in our previous study (Zhou et al. 2017). Most tested E2s were charged by both SlUBA1 and SlUBA2 with comparable efficiency (Figure S10, Table S2). However, E2s in groups IV (SlUBC32/SlUBC33/SlUBC34), V (SlUBC7/SlUBC14/SlUBC35/SlUBC36), VI (SlUBC4/SlUBC5/SlUBC6/SlUBC15) and XII (SlUBC22) exhibited differential charging, with SlUBA2 demonstrating significantly higher efficiency than SlUBA1 (Figure 4A, Figure S10). For instance, in group V, all E2s were efficiently charged by SlUBA2, whereas SlUBA1 charged SlUBC7 and SlUBC36 minimally and SlUBC14 and SlUBC35 with extremely low efficiency. Notably, the tomato E2s in groups IV, V and VI that are differentially charged by SlUBA1 and SlUBA2 fell into the same clades as human E2s that are differentially charged by human E1s UBE1 and UBA6 (Figure S11). These findings suggest that, like humans, tomato employs dual ubiquitin E1 activation systems with distinct E2 preferences.

### 2.5 The UFD of Tomato E1s Plays an Important Role in Determining the Specificity of E2 Charging

Prior studies in yeast and humans have shown that the ubiquitin‐fold domain (UFD) of E1s mediates E2 recruitment and is critical for specifying E2 charging (Lee and Schindelin 2008; Jin et al. 2007). To determine if the UFD of plant E1s serves a similar role, we constructed chimeric SlUBA1 and SlUBA2 proteins (SlUBA1‐UFD^SlUBA2^ and SlUBA2‐UFD^SlUBA1^) by swapping their UFD domains (Figure S12A,B). Both chimeric proteins charged the control E2, SlUBC3, with efficiencies comparable to those of wild‐type SlUBA1 and SlUBA2 (Figures 1A and 4A). In contrast, E2s from groups IV and V were charged by SlUBA1‐UFD^SlUBA2^ at markedly higher efficiencies than by wild‐type SlUBA1, reversing the pattern observed with native SlUBA1 and SlUBA2 (Figure 4B). This indicates that the UFD of tomato E1s is an important regulator of E2 charging efficiency. However, the slightly weaker charging of E2s by SlUBA1‐UFD^SlUBA2^ compared to SlUBA2 suggests that the UFD is not the sole determinant of specificity. This conclusion is reinforced by comparable interaction strengths observed between the UFDs of SlUBA1 and SlUBA2 and group IV E2s (Figure S12C), hinting at additional factors influencing E2 recognition.

### 2.6 The Gln^1009^ Residue of SlUBA2 Is Critical and Confers Specificity for E2 Charging

**FIGURE 5:**
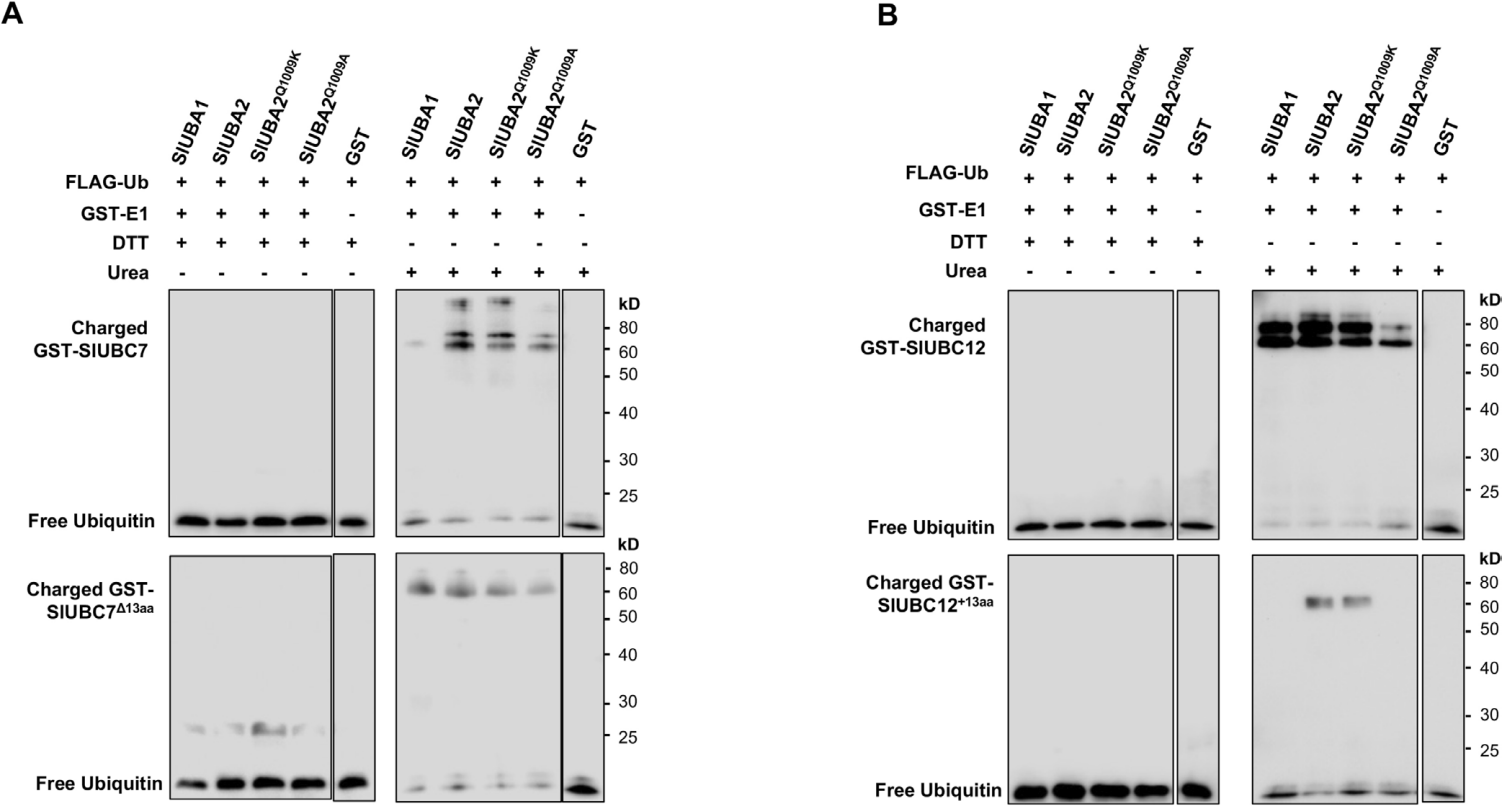
Both the Gln^1009^ in the ubiquitin fold domain (UFD) of SlUBA2 and variation of E2 contribute to the differential specificities in E2 charging by the tomato E1s. (A) Charging of SlUBC7 and SlUBC7^Δ13aa^ by SlUBA1, SlUBA2, SlUBA2^Q1009K^ and SlUBA2Q^1009A^ in thioester assay. The glutathione S‐transferase (GST) tag was used as a negative control. The numbers on the right denote the molecular mass of marker proteins in kDa. (B) Charging of SlUBC12 and SlUBC12^+13aa^ by SlUBA1, SlUBA2, SlUBA2^Q1009K^ and SlUBA2Q^1009A^ in thioester assay. The GST tag was used as a negative control. The numbers on the right denote the molecular mass of marker proteins in kilodaltons (kDa).

To further investigate the role of the UFD in differential E2 charging, we aligned the C‐terminal amino acid sequences containing the UFD of tomato (SlUBA1, SlUBA2) and *N. benthamiana* (NbUBA1a/1b, NbUBA2a/2b) E1 enzymes (Figure S13). The UFD sequences are highly conserved across these E1s, with variability limited to a few positions only. In particular, the Gln^1009^Asn^1010^ of UBA2 represents the only two consecutive residues of the UFD where tomato and *N. benthamiana* UBA2 and UBA1 possess different amino acids (amino acid with amidic side chain vs. aliphatic or acidic side chain). Extended sequence analysis of E1s from *Arabidopsis*, tomato, *N. benthamiana* and rice revealed that these positions exhibit significant variation among plant E1s, despite overall homology in UFD among these species (Figure S13). To test their functional importance, we generated SlUBA2 mutants, substituting Gln1009 with Lys (Q1009K) or Ala (Q1009A), and assessed their E2 charging activity. The SlUBA2^Q1009K^ mutant showed only a slight reduction in charging efficiency for SlUBC7 and SlUBC12 compared to wild‐type SlUBA2, whereas the SlUBA2^Q1009A^ mutant exhibited a marked decrease (Figure 5A,B, top panel). Furthermore, the charging efficiency difference between SlUBA2 and SlUBA1 for SlUBC7 was diminished in the Q1009A mutant. These results highlight Gln1009 as a key determinant of E2 charging specificity. The pronounced reduction in activity with SlUBA2^Q1009A^ mutant—versus the modest effect of SlUBA2^Q1009K^—suggests that the small, nonpolar Ala side chain disrupts charging more severely than the larger, polar Lys side chain, which shares chemical similarities with Gln.

Intriguingly, structural studies of human UBE1 and yeast UBA1 indicate that the region corresponding to Gln1009 (Figure S14) does not directly contact E2 but facilitates conformational changes essential for E1‐E2 thioester transfer (Lee and Schindelin 2008; Olsen and Lima 2013; Lv et al. 2018).

### 2.7 The Unique Feature of E2s Also Contributes to the Specificity of Their Charging by Tomato E1s

**FIGURE 6:**
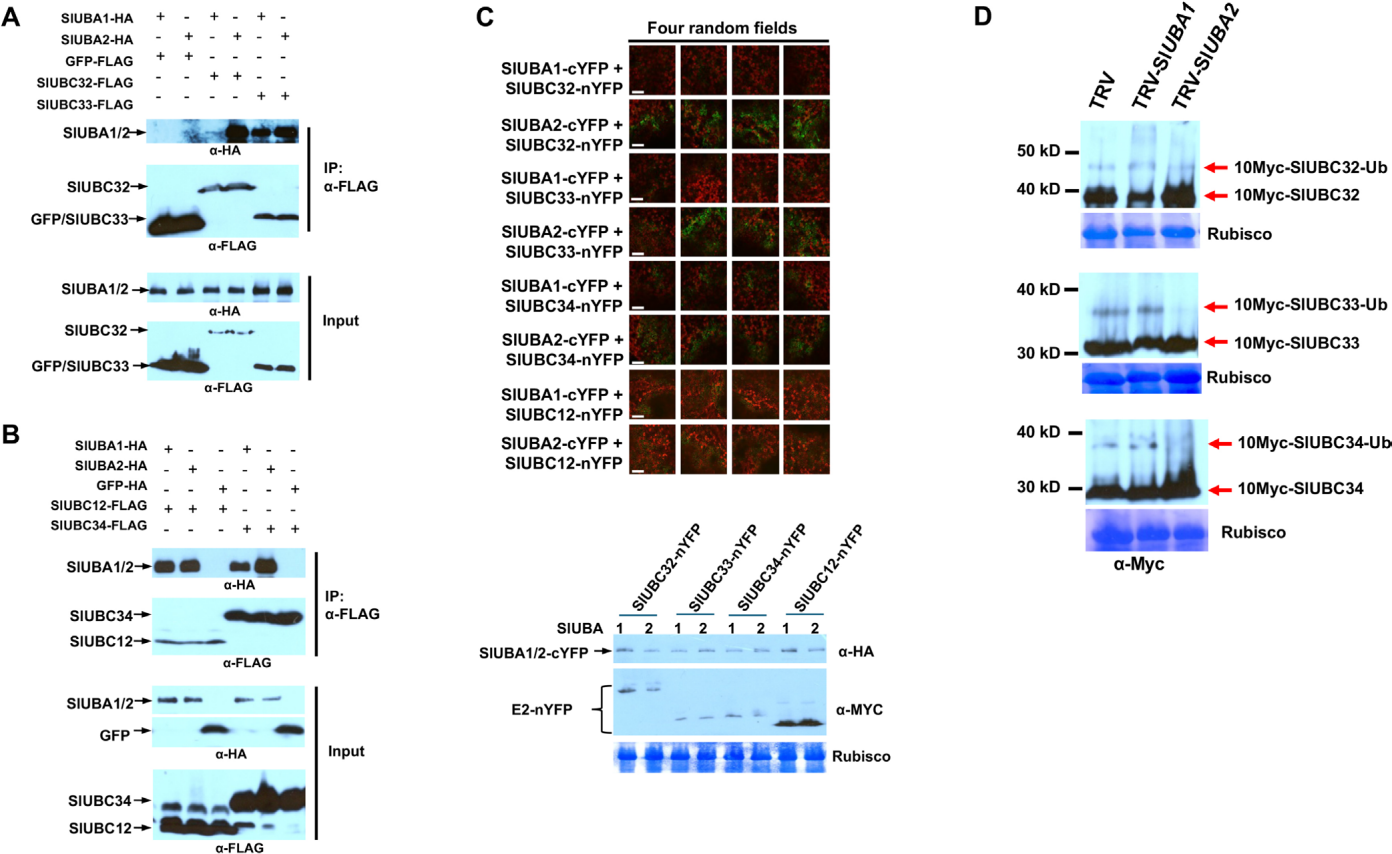
The tomato E1s exhibit differential intensities of interaction with group IV E2s in vivo. (A and B) Significantly higher amount of tomato SlUBA2 than SlUBA1 was pulled down by group IV E2s, SlUBC32, SlUBC33 and SlUBC34 in co‐immunoprecipitation assay. FLAG‐tagged group III E2 SlUBC12 and GFP were used as negative controls. (C) SlUBA2 showed significantly stronger interaction with SlUBC32, SlUBC33 and SlUBC34 than SlUBA1 in the bimolecular fluorescence complementation assay. Group III E2 SlUBC12 was used as control. Images from four random microscopic fields for each tested construct pair were presented. White bar represents a scale of 20 μm. Bottom panel shows the levels of protein expressed in planta for the E1 (SlUBA1 or SlUBA2)‐E2 pairs tested in the assay. (D) Silencing of *UBA2* affected the charging of group IV E2s in planta. 10Myc‐tagged tomato UBC32, UBC33 and UBC34 were transiently expressed in *NbUBA1‐* or *NbUBA2*‐silenced *Nicotiana benthamiana* plants. The adducts of SlUBC32‐, SlUBC33‐ and SlUBC34‐ubiquitin were detected by western blot using anti‐myc antibody. The empty TRV vector‐infected plants (TRV) were used as control. Coomassie Brilliant Blue staining of the RuBisCO large subunit was used as control for equal sample loading.

All ubiquitin E2 enzymes share a core catalytic UBC domain of approximately 150 amino acids, characterised by an α/β‐fold structure with four α‐helices and a four‐stranded β‐sheet (Ye and Rape 2009). Despite this conserved framework, sequence and subtle structural variations among E2s may influence their charging by E1 enzymes (Zhou et al. 2017; Stewart et al. 2016). To explore this, we aligned the protein sequences of tomato group V E2s (SlUBC7/14/35/36) with SlUBC3 and SlUBC12 (group III members) (Figure S15A), which are charged equally by SlUBA1 and SlUBA2 (Figures 4A, 5B, and 6B; Figure S10). Unlike SlUBC3 and SlUBC12, all group V E2s feature a highly conserved 13‐amino‐acid insertion predicted to reside within the loop connecting the fourth β‐strand and the second α‐helix of the UBC domain (Figure S15A,B). We thus generated a SlUBC7 mutant lacking this segment (SlUBC7^Δ13aa^) and assessed its charging. As shown in the bottom panel of Figure 5A, SlUBC7^Δ13aa^ exhibited significantly increased charging specificity by SlUBA1 but reduced specificity by SlUBA2, resulting in nearly equal charging by both E1s. Moreover, the E1 mutants SlUBA2^Q1009K^ and SlUBA2^Q1009A^ charged SlUBC7^Δ13aa^ less efficiently than wild‐type SlUBA2, with SlUBA2^Q1009A^ showing the greater reduction. These findings indicate that the 13‐amino‐acid insertion unique to group V E2s contributes to their differential charging by tomato E1s.

To further validate this observation, we engineered a SlUBC12 mutant (SlUBC12^+13aa^) by inserting the 13‐amino‐acid segment between Lys90 and Glu91 (Figure S14A) and tested its charging by SlUBA1, SlUBA2 and the SlUBA2 mutants (Figure 5B, bottom panel). Unlike wild‐type SlUBC12, which is charged with comparable specificity by both E1s (Figure 5B, top panel; Figure S10), SlUBC12^+13aa^ displayed differential charging, with SlUBA2 exhibiting significantly higher specificity and efficiency, mirroring the behaviour of SlUBC7 (Figure 5A, top panel). The SlUBA2^Q1009K^ mutant showed slightly reduced specificity compared to wild‐type SlUBA2, while SlUBA2^Q1009A^ failed to charge SlUBC12^+13aa^ entirely. These results reinforce the critical role of the Gln1009 residue in SlUBA2's E2 charging activity and demonstrate that the 13‐amino‐acid insertion alters E2 charging preferences. Collectively, these data suggest that specific structural features of E2s play a role in determining their charging by tomato E1 enzymes.

### 2.8 SlUBA2 Exhibits Stronger In Vivo Interactions With Group IV E2s Compared to SlUBA1

Our observation that tomato SlUBA1 and SlUBA2 differentially charge certain E2s in in vitro thioester assays led us to investigate whether similar differences occur in vivo by examining the ability and strength of E1‐E2 interactions. Recent studies have implicated plant group IV E2s—UBC32, UBC33, and UBC34—in endoplasmic reticulum‐associated protein degradation (ERAD) and highlighted their important roles in plant tolerance to biotic and abiotic stresses, with complex functional interplay among them (Wang et al. 2025). We thus selected SlUBC32, SlUBC33 and SlUBC34 as representative examples for our experiments. We employed three protein–protein interaction assays—co‐immunoprecipitation (Co‐IP), bimolecular fluorescence complementation (BiFC) and yeast two‐hybrid (Y2H)—to assess the strength of interaction between SlUBA1 and SlUBA2 and these E2s. These methods have been established for semiquantitative measurement of protein–protein interactions, provided the expression levels of the compared proteins are comparable (Wang et al. 2011; Roy et al. 2012; van der Geer 2014; Burckhardt et al. 2021; Kerppola 2006; Hu et al. 2002).

In the Co‐IP assay, SlUBC32, SlUBC33 and SlUBC34 consistently pulled down higher levels of SlUBA2 compared to SlUBA1 (Figure 6A,B). Similarly, the BiFC assay revealed markedly stronger in planta interactions between SlUBA2 and these E2s compared to SlUBA1 (Figure 6C, top panel). Importantly, the comparable expression levels of SlUBA1 and SlUBA2 proteins, along with their corresponding E2 proteins, confirmed that the observed differences in interaction strength were not due to variations in protein abundance (Figure 6C, bottom panel). The Y2H assay further corroborated these findings, demonstrating that group IV E2s interacted more strongly with SlUBA2 than with SlUBA1 (Figure S16A). Comparable protein expression levels were confirmed for all tested pairs of SlUBA1‐E2 and SlUBA2‐E2 (Figure S16B), ruling out differential protein levels as the cause of observed interaction differences.

Collectively, these results demonstrate that SlUBA2 exhibits stronger interactions with group IV E2s than SlUBA1 in vivo. To further validate the differential charging observed in vitro, we examined the charging of group IV E2s in planta by transiently expressing myc‐tagged E2s in *N. benthamiana* leaves, where expression of either *NbUBA1a/1b* or *NbUBA2a/2b* genes was silenced. Compared to control plants, silencing *NbUBA1a/1b* had no discernible effect on group IV E2 charging. In contrast, silencing *NbUBA2a/2b* nearly eliminated charging of these E2s (Figure 6D), suggesting that SlUBA2 predominantly mediates the charging of this E2 triplet in vivo.

### 2.9 The Two *Arabidopsis* E1s Differentially Charge Homologues of Tomato Group IV E2s

**FIGURE 7:**
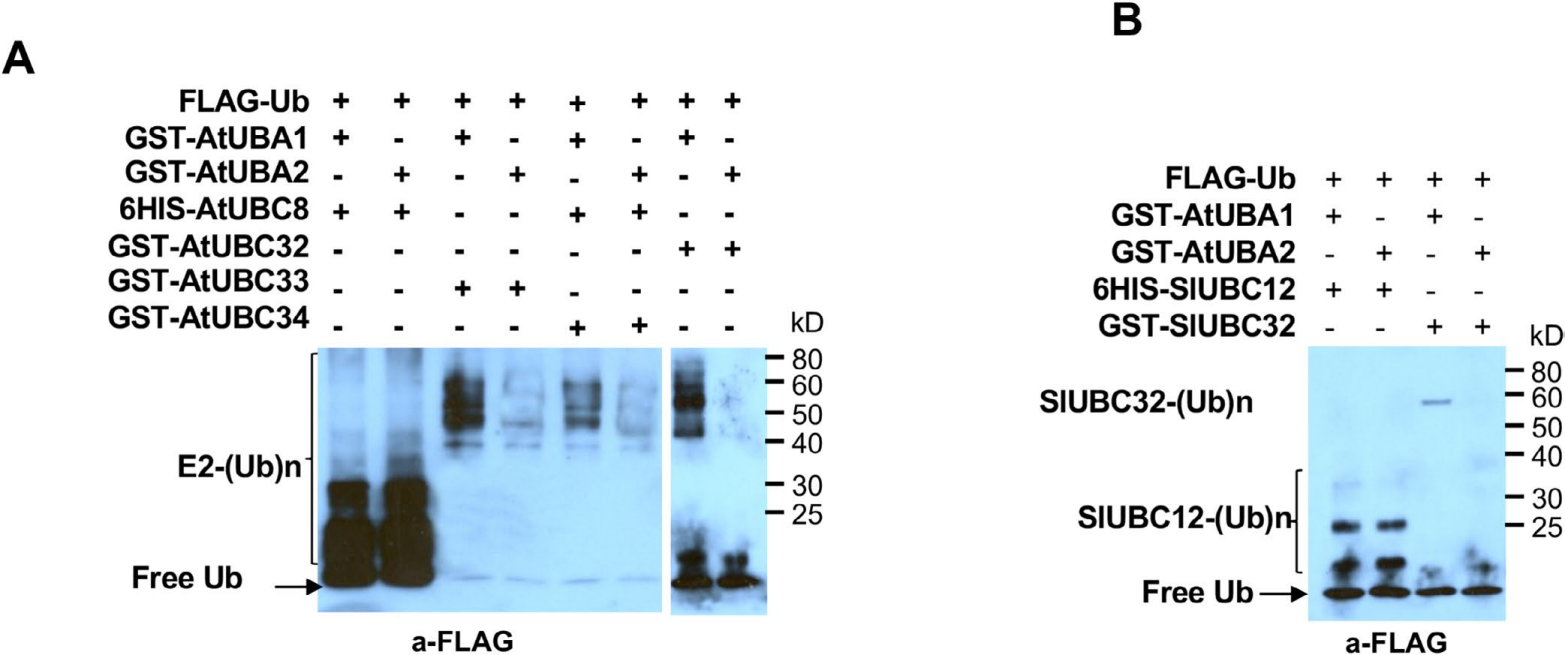
*Arabidopsis* E2 enzymes AtUBC32, AtUBC33 and AtUBC34 are differentially charged by the E1s AtUBA1 and AtUBA2. (A) The *Arabidopsis* E2s AtUBC32, AtUBC33 and AtUBC34 were charged by AtUBA1 at significantly higher efficiencies than that of AtUBA2 in thioester assay. The AtUBC8 was used as control. (B) Tomato SlUBC32 was charged by AtUBA1 with higher specificities than that of AtUBA2 in thioester assay. The tomato SlUBC12 was used as control.

As previously noted, most plant genomes encode two or more ubiquitin E1 enzymes (Table S1). Protein sequence alignments of plant E1 proteins from *Arabidopsis* (Hatfield et al. 1997), tomato, *N. benthamiana* (Wang et al. 2024), *N. tabacum* (Wang et al. 2024), soybean (Zhang et al. 2018) and rice revealed that the E1s are highly homologous, even though their N‐terminal regions show variations (Supporting Information S1). Additionally, the *Arabidopsis* homologues of group V E2s feature a highly conserved 13‐amino‐acid insertion (Figure S15), similar to their tomato counterparts. These observations led us to hypothesise that, in addition to tomato, E1s from other plant species are involved in differential E2 charging as well. To investigate this, we assessed the charging efficiency of the *Arabidopsis* group IV E2s—AtUBC32, AtUBC33 and AtUBC34—by the two *Arabidopsis* E1 enzymes, AtUBA1 and AtUBA2. Consistent with findings in tomato, AtUBC32, AtUBC33 and AtUBC34 were differentially charged by AtUBA1 and AtUBA2, with AtUBA1 exhibiting significantly greater specificity (Figure 7A). We further examined the charging efficiency of two tomato E2s, SlUBC12 and SlUBC32, by the *Arabidopsis* E1s. Intriguingly, AtUBA1 and AtUBA2 charged SlUBC12 with similar specificity, yet they differentially charged SlUBC32, with AtUBA1 again showing higher specificity (Figure 7B). These findings indicate that *Arabidopsis*, like tomato, maintains dual ubiquitin‐activating systems.

## 3 Discussion

**FIGURE 8:**
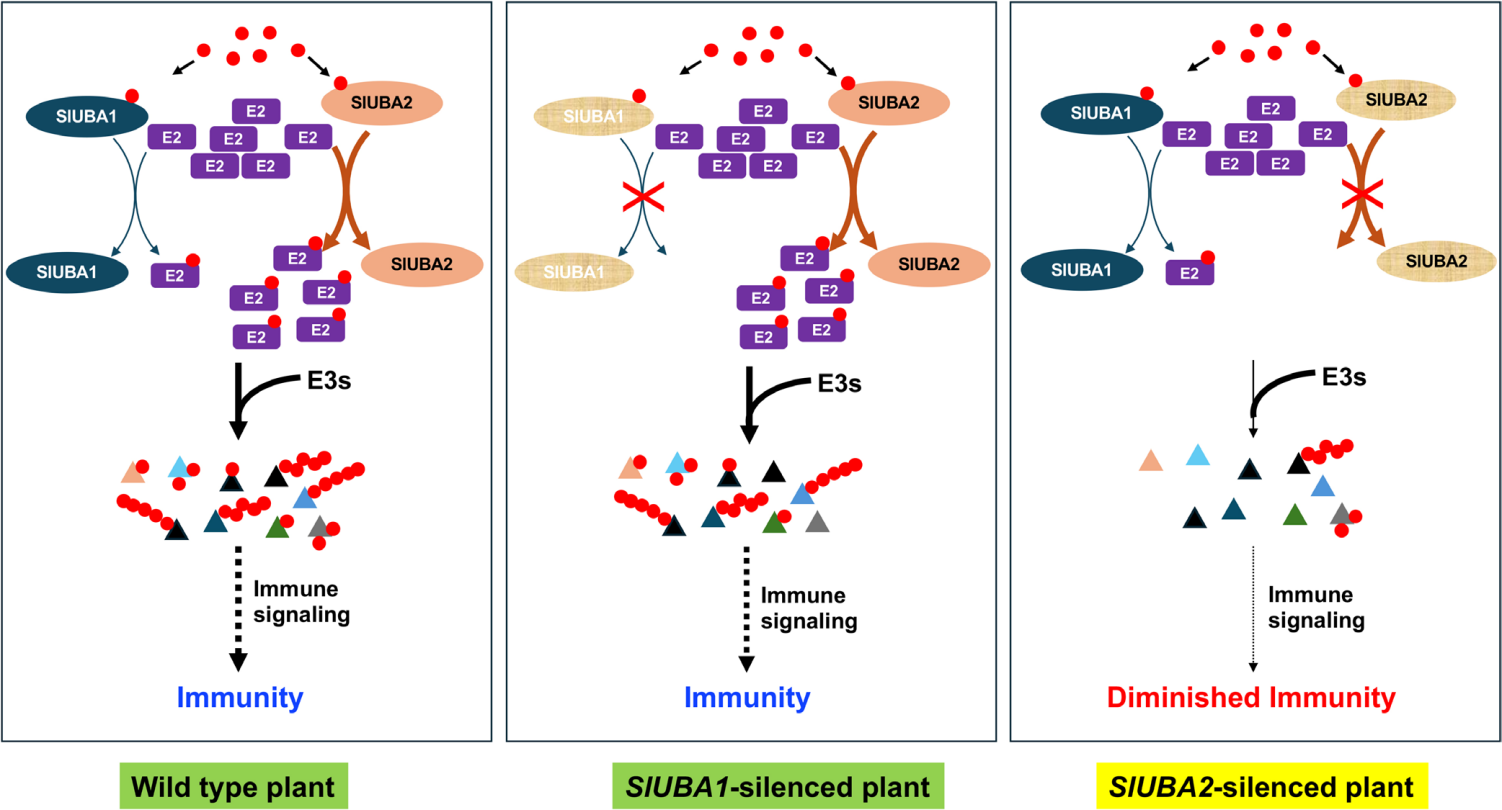
A working model illustrates how the tomato dual ubiquitin‐activating system (DUAS) functions distinctly in host immunity. The two tomato E1s exhibit differential E2 charging efficiencies toward groups IV, V, VI and XII E2s, with SlUBA2 displaying significantly higher efficiencies. Some of these E2s, such as those in group IV, are immunity‐associated. Differential charging of immunity‐associated E2s contributes to the unequal roles of SlUBA1 and SlUBA2 in plant immunity. In *SlUBA1*‐silenced plants, SlUBA2 effectively charges these E2s, enabling them to work with E3s to ubiquitinate substrate proteins associated with plant immunity. Consequently, plant immunity in these plants is not impaired. However, silencing the *SlUBA2* gene significantly reduces the charging of these E2s, impairing their cooperation with E3s to modulate plant immunity‐related substrates and weakening immunity. Nearly all sequenced plant genomes encode two or more ubiquitin E1s, suggesting that plants likely possess multiple ubiquitin E1 activation systems that do not play equal roles in various biological processes. Red dots denote ubiquitin molecules, purple‐filled rectangular boxes represent E2s (groups IV, V, VI and XII) that are differentially charged by SlUBA1 and SlUBA2, and coloured triangles represent various cognate substrate proteins. The different attachments of red dots to these proteins denote various types of ubiquitination. The thickness of the arrows indicates the relative efficiency or strength in E2 charging, ubiquitination of cognate substrates and relevant immune signalling.

In the current study, we reveal that tomato encodes two, whereas *N. benthamiana*, as an allotetraploid crop, encodes four, ubiquitin E1s, with NbUBA1a/1b being highly homologous to SlUBA1 and NbUBA2a/2b sharing high identity to SlUBA2. The UBA1 and UBA2 E1 enzymes play unequal roles in plant immunity and development. We demonstrate that the two E1 enzymes of tomato, SlUBA1 and SlUBA2, differentially charge E2 enzymes of groups IV (SlUBC32/SlUBC33/SlUBC34), V (SlUBC7/SlUBC14/SlUBC35/SlUBC36), VI (SlUBC4/SlUBC5/SlUBC6/SlUBC15) and XII (SlUBC22) in vitro and in vivo, with UBA2 showing significantly higher charging efficiency. The tomato group IV E2s were very recently shown to play important roles in plant immunity (Wang et al. 2025). Based on these results, we propose that SlUBA1 and SlUBA2 play unequal roles in host immunity by differential charging of E2 enzymes that are key to immunity (Figure 8). When *SlUBA1* is silenced, SlUBA2 can still charge these E2 enzymes efficiently. Therefore, the charging of these E2s in the cell is not affected; hence, plant immunity is not compromised. However, when *SlUBA2* is silenced, the charging of these E2s is significantly reduced because SlUBA1 has very low efficiency in charging them. This reduction affects their interaction with cognate E3s to ubiquitinate corresponding plant immunity‐related substrates, consequently compromising plant immunity (Figure 8). While group IV E2s have been confirmed to be critical for plant immunity, it is possible that E2s from groups V, VI and/or XII are also involved in plant immunity, contributing to the differential roles of the two E1s as well.

Ubiquitination is omnipresent and plays a critical role in plant growth, development, and immunity by modulating multiple phytohormone pathways (Kelley and Estelle 2012). Accordingly, E1 gene knockdowns likely impact multiple related processes. We examined the expression of *Indole‐3‐Acetic Acid Inducible 17* (*IAA17*, auxin signalling) (Gray et al. 2001), and *Jasmonate‐Zim Domain 1* (*JAZ1*, jasmonic acid signalling) (Pauwels and Goossens 2011) homologues in tomato and *N. benthamiana* plants with silenced *SlUBA1*, *SlUBA2* or both (*SlUBA1/2*) ubiquitin E1 genes. As shown in Figure S17, *IAA17* expression was reduced in all knockdown tomato plants but substantially decreased only in *SlUBA1‐* and *SlUBA1/2‐*knockdown *N. benthamiana* plants. *JAZ1* expression remained comparable to controls in tomato but was markedly reduced in *SlUBA1*‐ and *SlUBA1/2*‐knockdown *N. benthamiana* plants. These findings align with the altered growth and development observed in E1‐knockdown plants. However, the effects of ubiquitination on phytohormone signalling and responses are complex. The changes in plant growth, development and immunity in E1 gene‐silenced plants are likely a result of alterations in multiple phytohormone pathways, such as auxin, gibberellin, salicylic acid and jasmonic acid and their crosstalk in a spatiotemporal manner. Further identification of pathways differentially regulated by the two E1 proteins will enhance our understanding of ubiquitination's role in these processes.

Similar to yeast and human, the UFD of tomato E1s plays an important role in determining the specificities of E2 charging. The UFD of human and yeast E1s adopts a β‐grasp fold resembling the ubiquitin molecule and is required for the initial recruitment of E2s (Lee and Schindelin 2008; Olsen and Lima 2013). Although intensive structural studies have been conducted on human, animal and yeast E1s, no such study of plant ubiquitin E1 has been reported. Prediction using AlphaFold showed that the UFD of SlUBA1 and SlUBA2 also possesses a β‐grasp fold structure (Figure S14). Based on sequence alignment of E1s of tomato and *N. benthamiana*, we identified the two residues at the Gln^1009^ and Asn^1010^ of SlUBA2 UFD vary between tomato and *N. benthamiana* UBA1 and UBA2 proteins and confirmed that Gln^1009^ is important for the activity and efficiency of E2 charging by the tomato E1 (Figure 5). Though not tested, the Asn^1010^ is likely to be important for the E2 charging as well. Importantly, the amino acid residues corresponding to Gln^1009^ and Asn^1010^ also show high variation in other plant E1s examined (Figure S13), suggesting that the two residues likely also play a key role in determining the activity and efficiency of E2 charging by other plant E1s. In human UBE1 and yeast UBA1, the corresponding region is located at the opposite side of the E2‐interacting interface provided by the UFD and the second catalytic cysteine half‐domains (SCCH, part of the CCD domain) and is involved in facilitating the change of UFD from distal to proximal position to the E2 that is required for E1‐E2 thioester transfer (Lv et al. 2018; Olsen and Lima 2013). AlphaFold protein structure prediction with very high confidence suggests that the Gln^1009^ is located at the end of the first α‐helix of the UFD followed by a loop that links to the second β strand of the β sheet (Figure S14), which is highly similar to the corresponding region of human UBE1 and yeast UBA1. Thus, the Gln^1009^ residue and the region where it resides are likely also critical for conformational changes required for tomato E1‐E2 thioester transfer. Future structural study would help verify this projection.

In addition to the UFD of E1 enzymes, other factors may also contribute to the efficiency in plant E2 charging. In this study, we use the tomato group V E2 as an example and reveal that variations in E2 also play a role in the specificity of E2 charging. The tomato group V E2s and their counterparts from *Arabidopsis* all contain a highly conserved stretch of 13 amino acids insertion that is predicted to be part of the loop linking the fourth β strand and the second α helix of the UBC domain (Figure S15). This insertion is reminiscent of the acidic loop insertion in the *Schizosaccharomyces pombe* UBC15 and human homologues, such as hCDC34 and hUBE2G2 that lie in proximity to the E2 catalytic cysteine (Lv et al. 2017). The loop has been shown in many structural studies to be highly flexible, adopting multiple conformations ranging from folding back toward the E2 active site (‘closed’) to extending away from the active site (‘open’), thereby increasing accessibility of the active cysteine residue. Study of the crystal structure of *S. pombe* UBA1‐UBC15 complex suggests that an open position of the acidic loop facilitates E1‐E2 thioester transfer activity and deletion of the acidic loop of *S. pombe* Ubc15, human CDC34b and UBE2G2 increases the thioester transfer activities of the resulting E2 (Lv et al. 2017). In our study, deletion of the 13 amino acid residues in SlUBC7 resulted in increased efficiency in charging by SlUBA1 but reduced efficiency by SlUBA2, indicating the two tomato E1s interact differently with the loop in charging group V E2s. Interestingly, as shown in Figure S11, the tomato group V E2s are classified to the same clade in phylogenetic analysis as the human “family three” E2s, including CDC34, CDC34B and UBE2G2 that display differential specificities in charging by the human UBE1 and UBA6 (Jin et al. 2007), which suggests the acidic loop of this group of E2 generally plays a role in determining the specificities of charging by E1s. Similarly, it is expected that variations in group IV, VI and XII E2s may contribute to their specificity of being charged by E1 as well.

Given that most plants possess more than one ubiquitin E1 enzyme (Table S1) and the E2 enzymes are relatively highly conserved among plants (Zhou et al. 2017; Zhang et al. 2018), it is likely that plants generally possess more than one ubiquitin E1 activation system by differentially charging certain subgroups of E2s to fulfil unequal roles in different biological processes. This notion was supported by our results that tomato SlUBA1 and SlUBA2 differentially charge 12 out of the 34 tested E2s and *Arabidopsis* E1s AtUBA1 and AtUBA2 also differentially charge the E2s AtUBC32, AtUBC33 and AtUBC34. With more than one ubiquitin E1 activation system endowed with unequal roles in certain biological processes, a plant has the flexibility of both functional redundancy and specificity in modulating numerous pathways by ubiquitination. The two (or more) ubiquitin E1‐activation systems in a plant can work either in parallel, or temporarily and/or spatially separated, or both to allow regulation of different pathways with plasticity yet precision. In this regard, the E2 enzymes that are differentially charged by plant E1s and their cognate E3s would serve as intriguing targets for studying the pathways/processes they modulate, such as plant immunity. Identification and characterisation of such E2 enzymes and the cognate E3 enzyme with which they work will open a new avenue for probing the regulation and fine‐tuning of plant immunity by ubiquitination.

In addition to intrinsic efficiency in charging various E2s by the ubiquitin E1, other factors, such as posttranslational modifications that affect the activities and localisations of E1s and E2s, might also contribute to the distinct roles of the plant ubiquitin E1 activation systems. In mammalian cells, S‐glutathionylation was reported to suppress E1 and E2 activity (Huang et al. 1993). Human E1 enzyme UBE1 and E2 enzymes were found to be phosphorylated in vitro and in vivo (Cook and Chock 1995; Kong and Chock 1992; Stephen et al. 1996). The human Casein kinase 2 (CK2) phosphorylates E2 Cell Division Cycle 34 (CDC34) to regulate its subcellular localisation (Block et al. 2001). In addition, the import and/or retention of human ubiquitin E1 UBE1 to the nucleus is cell cycle‐dependent (Stephen et al. 1996). More recently, a structural study of the yeast E1‐E2 (UBC15) complex suggests that phosphorylation of residues at the N termini of ubiquitin E2s broadly inhibits their ability to function with ubiquitin E1 (Lv et al. 2017). Although no plant E1 and E2 enzymes for ubiquitination have been shown to be modified by other posttranslational modifications, it is believed that such modifications do exist in plants (Zhang and Zeng 2020), which may serve as an extra layer of modulation in addition to differential E2 charging that leads to unequal roles of the plant ubiquitin E1 enzymes.

## 4 Experimental Procedures

### 4.1 Growth of Bacteria and Plant Materials

*Agrobacterium tumefaciens* strains GV3101 and GV2260 were grown on Luria Bertani medium and strains of Pst and *Pseudomonas fluorescens* 55 were grown on King's B medium, at 28°C with appropriate antibiotics. *N. benthamiana* and tomato RG‐pto11 (*pto11/pto11*, *Prf/Prf*) seeds were germinated and plants were grown on autoclaved soil in a growth chamber with 16 h light (~300 μmol/m^2^/s at the leaf surface of the plants), 24°C/23°C day/night temperature, and 50% relative humidity.

### 4.2 DNA Manipulations and Plasmid Constructions

Standard molecular biology techniques were employed for DNA manipulations (Green et al. 2012). See the Supporting Information file for details.

### 4.3 Sequence Alignment and Phylogenetic Analysis

For sequence alignment, sequences of interest in the FASTA format were entered into the Clustal Omega programme and aligned using the Clustal Omega algorithm (Sievers et al. 2011). See the Supporting Information file for details.

### 4.4 Expression and Purification of Recombinant Proteins

GST‐tagged fusion proteins were expressed in *E. coli* BL21 (DE3) and purified with Glutathione Sepharose 4 Fast Flow beads (GE Healthcare) by following the protocol provided by the manufacturer. The purified proteins were further desalted and concentrated in the protein storage buffer (50 mM Tris–HCl pH 8.0, 50 mM KCl, 0.1 mM EDTA, 1 mM dithiothreitol [DTT], 0.5 mM phenylmethylsulfonyl fluoride [PMSF]) using the Amicon centrifugal filter (Millipore). The desalted and concentrated recombinant protein was stored at −80°C in the presence of a final concentration of 40% glycerol until being used. The concentration of purified protein was determined using protein assay agent (Bio‐Rad).

### 4.5 Examination of Charging Ubiquitin E2s by E1s via Thioester Assay

To examine the efficiencies of charging E2s by E1s, the thioester assay was performed as described with modifications (Mural et al. 2013). See the Supporting Information file for details.

### 4.6 RT‐qPCR

For detecting gene expression, samples of tomato root, stem, leaf, sepal, petal, ovary and green fruit from 10‐week‐old tomato plants; leaf tissues of 3‐ to 4‐week‐old tomato RG‐pto11 (*pto11/pto11*, *Prf/Prf*) plants infiltrated with 2 μM flg22 or sterile water (mock, used as control); leaf tissues of *N. benthamiana* E2‐RNAi transgenic lines and VIGS plants; and leaf tissues from *Arabidopsis* plants with different treatments were collected for total RNA extraction using the RNeasy Plant Mini Kit with DNase treatment (QIAGEN) by following the protocol provided by the manufacturer. The first‐strand cDNA was synthesised using the Superscript III reverse transcriptase and oligo(dT) primer (Life Technologies) according to the instructions from the manufacturer. Quantitative real‐time PCR (qPCR) was performed using gene‐specific primers and SYBR Green (Life Technologies) on the LightCycler 480 Instrument II (Roche). All primers used in RT‐qPCR are shown in Table S3. *S* *lEF1a*, *NbEF1a* and *AtActin2* were used as the internal references for tomato, *N. benthamiana* and *Arabidopsis* samples, respectively.

### 4.7 Y2H Assays

For testing the interaction of two proteins using the LexA‐based yeast two‐hybrid system, procedures were followed as described (Golemis et al. 2008). See the Supporting Information file for details.

### 4.8 BiFC Assay

The BiFC assay that is based on split yellow fluorescent protein (YFP) was used to test the interaction of various E1‐E2 pairs in the leaves and protoplasts (Chen et al. 2006; Waadt et al. 2008). See the Supporting Information file for details.

### 4.9 Co‐IP Assay

The coimmunoprecipitation assay of HA‐tagged E1s and FLAG‐tagged E2s was performed as described previously with some modifications (Zhou et al. 2017; Moffett et al. 2002). See the Supporting Information file for details.

### 4.10 VIGS

Gene silencing was induced using the tobacco rattle virus (TRV) vectors as previously described (Mural et al. 2013). See the Supporting Information file for details.

### 4.11 Extraction of Plant Total Proteins and Immunoblotting

Each tomato and *N. benthamiana* sample was homogenised in 300 μL 1× Laemmli buffer and then boiled for 5 min, followed by being resolved using 10% SDS‐PAGE. Each *Arabidopsis* sample was homogenised in 300 μL protein extraction buffer (25 mM Tris–HCl, pH 7.5, 150 mM NaCl, 5% glycerol, 0.05% Nonidet P‐40, 2.5 mM EDTA, 1 mM PMSF, and 1× complete cocktail of protease inhibitors). The concentration of total proteins was determined using protein assay agent (Bio‐Rad). Extraction of each sample containing 20 μg proteins was added to 2× SDS protein loading buffer and boiled for 5 min, then resolved by 10% SDS‐PAGE. The immunoblottings were performed with appropriate antibodies: anti‐FLAG (Sigma), anti‐HA (Sigma), anti‐myc (Santa Cruz), and anti‐Ub (P4D1) (Santa Cruz).

### 4.12 Bacterial Population Assay

The bacterial population assay was conducted as described previously (Nguyen et al. 2010). See the Supporting Information file for details.

### 4.13 Cell Death Suppression Assay

The cell death suppression assay was performed as previously described (Nguyen et al. 2010). *P. fluorescens* 55 at a concentration of OD_600_ equal to 0.5 (~2.5 × 10^8^ CFU/mL), 0.1 (~5 × 10^7^ CFU/mL) and 0.015 (~7.5 × 10^6^ CFU/mL) was used as the PTI inducer. Pst DC3000 at a concentration of 2 × 10^6^ CFU/mL was used as the challenger in the assay. The challenge of PTI was conducted 7 h after PTI induction. The appearance of cell death in the overlapping area, where both the inducer and challenger were infiltrated, was assessed. Photographs were taken on the fourth day after the infiltration of Pst DC3000.

Except for Figures 1A, 5B, and 6D, Figures S9C, S13, and S17 that were conducted with two biological replicates, all other experiments in this study were performed with three biological replicates, yielding similar results.

## Author Contributions

**Chaofeng Wang:** investigation, formal analysis, writing – review and editing. **Bangjun Zhou:** methodology, investigation, formal analysis, writing – review and editing. **Xuanyang Chen:** investigation. **Lirong Zeng:** methodology, formal analysis, writing – original draft preparation, review and editing.

## Conflicts of Interest

The authors declare no conflicts of interest.

## Supporting information


**Data S1:** Protein sequence alignment of ubiquitin E1 enzymes from Arabidopsis, tomato, *N. benthamiana*, *Nicotiana tabacum* , soybean and rice.

**Data S2:**Detailed description of experimental procedures for some experiments.

**Figure S1:** Tomato E1 proteins SlUBA1 and SlUBA2 possess typical domain organisation of ubiquitin E1 enzymes.

**Figure S2:** Phylogenetic analysis of ubiquitin E1 proteins from Arabidopsis, tomato, *N. benthamiana*, rice and human.

**Figure S3:** DNA sequence alignment of tomato *SlUBA1* and *N. benthamiana* E1 genes *NbUBA1a* and *NbUBA1b*.

**Figure S4:** DNA sequence alignment of tomato *SlUBA2* and *N. benthamiana* E1 genes *NbUBA2a* and *NbUBA2b*.

**Figure S5:** DNA sequence alignment of tomato *SlUBA1* and *SlUBA2* gene fragments used for virus‐induced gene silencing (VIGS) of *SlUBA1/NbUBA1a/1b* and *SlUBA2/NbUBA2a/2b*, respectively and corresponding regions of *NbUBA1a/1b* and *NbUBA2a/2b* genes.

**Figure S6:** The DNA fragments used for VIGS of *SlUBA1*/*NbUBA1a/*1b and *SlUBA2 NbUBA2a/2b*, respectively share minimal homology.

**Figure S7:** The ubiquitin E1 genes *SlUBA1* and *SlUBA2*, *NbUBA1a/1b* and *NbUBA2a/2b* were specifically and efficiently silenced in tomato and *N. benthamiana* plants.

**Figure S8:** Effects of E1 gene silencing on leaf development in tomato and *Nicotiana benthamiana*.

**Figure S9:** Effects of E1 gene silencing on leaf development in tomato and *Nicotiana benthamiana*.

**Figure S10:** Systematic analysis of the efficiencies in charging ubiquitin E2 enzymes by SlUBA1 and SlUBA2.

**Figure S11:** Comparison of the efficiencies in E2 charging by Human and tomato E1s.

**Figure S12:** The UFD is an important but not sole factor that governs the specificities of E2 charging by tomato ubiquitin E1s.

**Figure S13:** Protein sequence alignment of C‐terminal region of ubiquitin E1s from Arabidopsis, tomato, *N. benthamiana* and rice.

**Figure S14:** Predicted three‐dimensional structure of tomato E1s SlUBA1 and SlUBA2 by AlphaFold3.

**Figure S15:** Protein sequence alignment of tomato and Arabidopsis UBC3, UBC12 and group V E2s, and predicted three‐dimensional structure of tomato UBC7 and UBC12 by AlphaFold3.

**Figure S16:** Group IV E2s exhibit stronger interaction with SlUBA2 than with SlUBA1 in yeast two‐hybrid assay.

**Figure S17:** Expression levels of *IAA17* and *JAZ1* homologues in tomato and N. benthamiana plants with silenced ubiquitin E1 genes.

**Table S1:** Ubiquitin‐activating enzymes (E1) encoded by crop and model plant genomes.

**Table S2:** SlUBA1 and SlUBA2 show differential efficiencies to four groups of E2s.

**Table S3:** List of primers used for this study.

## Data Availability

Sequence data of tomato, Arabidopsis, rice, and soybean ubiquitin E1 and E2 proteins that were used in this article can be found in the GenBank library of the National Center for Biotechnology Information (NCBI), https://www.ncbi.nlm.nih.gov/genbank/based on the accession numbers: tomato E1s SlUBA1 (Solyc06g007320.2.1), XP_004240416; SlUBA2 (Solyc09g018450.2.1), XP_004246264; soybean E1s GmUBA1 (Glyma.14G196800), KRH17078; GmUBA2 (Glyma.11G166100), XP_006591250; GmUBA3 (Glyma.02G229700), XP_003518319; GmUBA4 (Glyma.18G058900), XP_006602078; Arabidopsis E1s AtUBA1 (AT2G30110), NP_565693; AtUBA2 (AT5G06460), NP_568168; rice E1s LOC_Os12g01520.1, ABA95612.2; LOC_Os11g01510.2, XP_015616970; LOC_Os07g49230.1, XP_015647669; LOC_Os03g18380.3, XP_015632802; Arabidopsis E2s AtUBC32 (AT3G17000), NP_566563; AtUBC33 (AT5G50430), NP_199854; AtUBC34 (AT1G17280), NP_001077554; tomato E2s SlUBC32 (Solyc12g099310.1.1), KY246924; SlUBC33 (Solyc03g123660.2.1), KY246925; SlUBC34 (Solyc06g063100.2.1), KY246926; SlIAA17 (Solyc06g053830), XP_004241030; SlJAZ1 (Solyc07g042170), XP_004243696. The sequence of ubiquitin E1 proteins from *Nicotiana benthamiana* and *Nicotiana tabacum* can be found at the Nicotiana benthamiana and tabacum Omics database, http://lifenglab.hzau.edu.cn/Nicomics/index.php (Wang et al. 2024): NbUBA1a, Nbe03g13750.1; NbUBA1b, Nbe04g02160.1; NbUBA2a, Nbe14g09490.1; NbUBA2b, Nbe18g13930.1; NtUBA1a, Nta03g01970.2; NtUBA1b, Nta04g02230.2; NtUBA2a, Nta17g06720.1; NtUBA2b, Nta18g08720.1; NbIAA17.1, Nbe01g31880; NbIAA17.2, Nbe02g04210; NbJAZ1.1, Nbe14g17870; NbJAZ1.2, Nbe16g02010.
