# Supplementary material for: The Two Tomato Ubiquitin E1 Enzymes Play Unequal Roles in Host Immunity
Source: Mol Plant Pathol. 2025 Sep 29;26(10):e70160. doi: 10.1111/mpp.70160 (PMC12477439; doi:10.1111/mpp.70160)
Supplement: Supplementary file 1 — Data S1: Protein sequence alignment of ubiquitin E1 enzymes from Arabidopsis, tomato, N. benthamiana, Nicotiana tabacum , soybean and rice. [file MPP-26-e70160-s022.docx]

**Supplementary data file I: Protein sequence alignment of ubiquitin E1 enzymes from Arabidopsis, tomato, *N. benthamiana*, *Nicotiana tabacum*, soybean, and rice**

XP_015647669.1(LOC_Os07g49230.1) ----------------------------MRCLRFLRRGLLSMLPTNKRAAGTDDDRP--- 29

XP_015632802.1(LOC_Os03g18380.3) -----------------------------------------MLPTKRA-NGAEAESS--- 15

ABA95612.2(LOC_Os12g01520.1) -----------------------------------------MLTRKRE------------ 7

XP_015616970.1(LOC_Os11g01510.2) -----------------------------------------MLTRKRE------------ 7

SlUBA2 -----------------------------------------MLPVKRSSIVEVGGDNDGG 19

NbUBA2a(Nbe14g09490.1) -----------------------------------------MLPVKR--TVEVGGENDTI 17

NbUBA2b(Nbe18g13930.1) -----------------------------------------MLPVKR--TVDVGGENDTI 17

NtUBA2a(Nta17g06720.1) -----------------------------------------MLPVKR--TVEVGGENDTV 17

NtUBA2b(Nta18g08720.1) -----------------------------------------MLPVKR--TVEVGGENDDV 17

AtUBA2 ------------------------------------------------------------ 0

AtUBA1 -----------------------------------------MLH-KRAS----------E 8

SlUBA1 -----------------------------------------MLPRKRPAEGVVVEG---- 15

NbUBA1a(Nbe03g13750.1) ------------------------------------------------------------ 0

NbUBA1b(Nbe04g02160.1) -----------------------------------------MLPRKRSAEGVVVDGDGDG 19

NtUBA1b(Nta04g02230.2) -----------------------------------------MLPRKRSAEGVVVDGD--G 17

NtUBA1a(Nta03g01970.2) -----------------------------------------MLPRKRSAEGVVVDGD--G 17

Glyma.02G229700.1 -----------------------------------------MLPRKRASEGGVVVEGDTD 19

Glyma.14G196800.1 MDLLLYYYDFNFNLIVFAFVLLIISMVRFRCV--FCSLLHYMLPRKRVREGGVVVEVDSD 58

Glyma.11G166100.1 -----------------------------------------MLPTKRPCEGLVAEEEIDH 19

Glyma.18G058900.1 -----------------------------------------MLPTKRPCEGLVAEEETHN 19

XP_015647669.1(LOC_Os07g49230.1) -----------------------------TD-PK-RPK---------------------- 36

XP_015632802.1(LOC_Os03g18380.3) -----------------------------SDAPAKKARVGASASEAE------------- 33

ABA95612.2(LOC_Os12g01520.1) ----------------------------------------------------------DL 9

XP_015616970.1(LOC_Os11g01510.2) ----------------------------------------------------------EL 9

SlUBA2 ----------------------------SVDPLTKKYKTAAAAAGGDSSTVTMAGAGSA- 50

NbUBA2a(Nbe14g09490.1) ----------------------------SVDPLTKKHKAIAAA-AGESSMVTMGGAGSAT 48

NbUBA2b(Nbe18g13930.1) ----------------------------SVDPLTKKHKATAAA-AGDSSTVTMGGAGSAT 48

NtUBA2a(Nta17g06720.1) ----------------------------SVDPLTKKHKATAAA-AGDSSTVTMGGAGSAI 48

NtUBA2b(Nta18g08720.1) ----------------------------SVDPLTKKHKATAAA-SGDSSTVTMGGTGSAT 48

AtUBA2 ----------------MEPFVVKENIIASASSPMKKRRIDHTESADGSAINASNS----- 39

AtUBA1 A----------------NDKNDNTIIG-SDLASSKKRRIDFTESSSD--------KSSSI 43

SlUBA1 NSSSC-----------------------DPESSLKKHKISCVIS-SGTEENTSGCSSNKV 51

NbUBA1a(Nbe03g13750.1) ------------------------------------------------------------ 0

NbUBA1b(Nbe04g02160.1) DSSSC-----------------------DPERVLKKHRISCVIS-SGAKENTSGCSTNKI 55

NtUBA1b(Nta04g02230.2) NSSSC-----------------------DPERVLKKHRISCVIS-SGAKENTSGCSTNKI 53

NtUBA1a(Nta03g01970.2) NSSSC-----------------------DPERVLKKHRISCVIS-SGAKENTSGCSTNKI 53

Glyma.02G229700.1 PTN-SSNSG--------------------AASFSKKARIGSLAACSGAGAAESAVN---V 55

Glyma.14G196800.1 ATTTNTNSA--------------------AASFPKKARIGSFAACSGAGAADSPVN---V 95

Glyma.11G166100.1 NINNNNNS---------------------NSSSLKKKRIA-------AGTADSTVKNDES 51

Glyma.18G058900.1 NNDNNNNSNNNNNNNNNNNNNNNSNNSSSSSSSLKKNRIAA------ARTADSTVKNYES 73

XP_015647669.1(LOC_Os07g49230.1) ---------------------VAQNGSTNGVVVPEIDEDLHSRQLAVYGRETMRRLFASH 75

XP_015632802.1(LOC_Os03g18380.3) ---AMVA---GEAGG------GGGGVSGNGSEVAEIDEDLHSRQLAVYGRETMRRLFASN 81

ABA95612.2(LOC_Os12g01520.1) AGEVVH-DLHK--KTRADDEPADDNHTMTTGRAPEIDEDLHSRQLAVYGRETMKRLFASN 66

XP_015616970.1(LOC_Os11g01510.2) AGE-VH-DLHK--KTRADDEPADDNHTMTTGRAPEIDEDLHSRQLAVYGRETMKRLFASN 65

SlUBA2 TGDVSAN---GNATNG----RTGGVSPVDLRNLSDIDEDLHSRQLAVYGRETMRKLFAAN 103

NbUBA2a(Nbe14g09490.1) TGDVNAN---GNAAN--------GKSPIDARNSPDIDEDLHSRQLAVYGRETMRRLFASN 97

NbUBA2b(Nbe18g13930.1) SGDVNAN---GNTAN--------GKSPIDARNSPDIDEDLHSRQLAVYGRETMRRLFASN 97

NtUBA2a(Nta17g06720.1) TGDVNAN---GNATN--------GKSPIDARNSPDIDEDLHSRQLAVYGRETMRRLFASN 97

NtUBA2b(Nta18g08720.1) TGDVNTN---GNATN--------GKSPIDARNSPDIDEDLHSRQLAVYGRETMRRLFASN 97

AtUBA2 SSIGLNN-----SIGGNDTVMSMAEFGNDNSNNQEIDEDLHSRQLAVYGRETMRKLFASN 94

AtUBA1 LASGSSRGF------HGDSVVQQIDMAFGNSNRQEIDEDLHSRQLAVYGRETMRRLFASN 97

SlUBA1 VSNNTN----GNTSSGSVGERSVTEMAFDDGNPHDIDEDLHSRQLAVYGRETMRRLFASN 107

NbUBA1a(Nbe03g13750.1) -------------------------MAFDDGNPHDIDEDLHSRQLAVYGRETMQRLFASN 35

NbUBA1b(Nbe04g02160.1) VGNNFK----GNASSSSAGEQSVTEMAFDDGNPHDIDEDLHSRQLAVYGRETMRRLFASN 111

NtUBA1b(Nta04g02230.2) VGNNFK----GNASSSSAGEQSVTEMAFDDGNPHDIDEDLHSRQLAVYGRETMRRLFASN 109

NtUBA1a(Nta03g01970.2) LGKNFK----GNASSSSAGEQSVIEMAFDDGNPHDIDEDLHSRQLAVYGRETMRRLFASN 109

Glyma.02G229700.1 SGQGFGSG----S-GDDSVGNSVGGMALGNSQPAEIDEDLHSRQLAVYGRETMRRLFASS 110

Glyma.14G196800.1 SGQGFSSG----GGGDNSLGNSVGGMALGNSHPAEIDEDLHSRQLAVYGRETMRRLFASS 151

Glyma.11G166100.1 TVRSFNNSNSNNSSNSGDASEGASDMALGESNPPDIDEDLHSRQLAVYGRETMRRLFGSN 111

Glyma.18G058900.1 TDQSFNN----NNSNSGNASEGASDMALGESNQPDIDEDLHSRQLAVYGRETMRRLFGSN 129

:******************::**.:

XP_015647669.1(LOC_Os07g49230.1) VLVSGLNGLGAEIAKNLALAGVKSVTLHDVKNVEMWDLSANFFLSENDIGKNRAAACVSK 135

XP_015632802.1(LOC_Os03g18380.3) VLVSGLNGLGAEIAKNLALAGVKSITLHDMGNVEMWDLSGNFFLSEDDIGKNRAVACTAK 141

ABA95612.2(LOC_Os12g01520.1) VLVSGLNGLGAEIAKNLVLAGVKSVTLHDDDNVELWDLSSNFFLTEKDVGQNRAQTCVQK 126

XP_015616970.1(LOC_Os11g01510.2) VLVSGLNGLGAEIAKNLVLAGVKSVNLHDDDNVELWDLSSNFFLTEKDVGQNRAQTCVQK 125

SlUBA2 VLISGLQGLGAEIAKNLILAGVKSVTLHDEGNVELWDLSSNFIFTEEDVGKNRALASVQK 163

NbUBA2a(Nbe14g09490.1) VLVSGLQGLGAEIAKNLILAGVKSVTLHDEGNVELWDLSSNFIFTEEDVGKNRALASIQK 157

NbUBA2b(Nbe18g13930.1) VLVSGLQGLGAEIAKNLILAGVKSVTLHDEGNVELWDLSSNFIFTEEDVGKNRALASIQK 157

NtUBA2a(Nta17g06720.1) VLVSGLQGLGAEIAKNLILAGVKSVTLHDEGNVELWDLSSNFIFTEEDVGKNRALASIQK 157

NtUBA2b(Nta18g08720.1) VLVSGLQGLGAEIAKNLILAGVKSVTLHDEGNVELWDLSSNFIFTEEDVGKNRALASIQK 157

AtUBA2 VLISGMQGLGVEIAKNIILAGVKSVTLHDENVVELWDLSSNFVFTEEDIGKNRALASVHK 154

AtUBA1 VLISGMHGLGAEIAKNLILAGVKSVTLHDERVVELWDLSSNFVFSEDDVGKNRADASVQK 157

SlUBA1 VLVSGIQGLGAEIAKNLILAGVKSVTLHDEGEVQLWDLSSNFIFSESDVGMNRALASVQK 167

NbUBA1a(Nbe03g13750.1) VLVSGIQGLGAETAKNLILAGVKSVTLHDEGEVQLWDLSSNFIFSESDVGKNRALASVQK 95

NbUBA1b(Nbe04g02160.1) VLVSGIQGLGAETAKNLILAGVKSVTLHDEGEVQLWDLSSNFIFSESDVGKNRALASVQK 171

NtUBA1b(Nta04g02230.2) VLVSGIQGLGAETAKNLILAGVKSVTLHDEGEVQLWDLSSNFIFSESDVGKNRALASVQK 169

NtUBA1a(Nta03g01970.2) VLVSGIQGLGAETAKNLILAGVKSVTLHDEGEVQLWDLSSNFIFSESDVGKNRALASVQK 169

Glyma.02G229700.1 ILVSGMQGLGVEIAKNLILAGVKSVTLHDEGNVELWDLSSNFVFSENDVGKNRAEASVGK 170

Glyma.14G196800.1 VLVSGMQGLGVEIAKNLILAGVKSVTLHDEENVELWDLSSNFVFSENDVGKNRAEASVSK 211

Glyma.11G166100.1 VLVSGMQGVGVEIAKNLILAGVKSVTLHDEGTVELWDLSSNFVFSENDVGKNRAAASVSK 171

Glyma.18G058900.1 VLVSGMQGLGVEIAKNLILAGVKSVTLHDEGTVELWDLSSNFVFSENDVGKNRAAASVSK 189

:*:**::*:*.* ***: ******:.*** *::****.**.::*.*:* *** :. *

XP_015647669.1(LOC_Os07g49230.1) LQELNNAVLVSALTEELTTDHLSKFQAVVFTDIGLDKAYEFDDYCHSHCPPISFIKAEVC 195

XP_015632802.1(LOC_Os03g18380.3) LQELNNAVLISTLTEDLTNEHLSKFQAVVFTDISLDKAFEFDDYCRNHQPSISFIKAEVC 201

ABA95612.2(LOC_Os12g01520.1) LQELNNAVIISTITGDLTKEQLSNFQAVVFTDISLEKAVEFDSYCHNHQPPIAFIKSEIR 186

XP_015616970.1(LOC_Os11g01510.2) LQELNNAVIISTITGDLTKEQLSNFQAVVFTDISLEKAVEFDSYCHNHQPPIAFIKSEIR 185

SlUBA2 LQELNNTVIISTLTDALTKEQLSNFQAVVFTDISLENAFEFDDYCHMHQPPIAFIKTEVR 223

NbUBA2a(Nbe14g09490.1) LQELNNSVIISTLTDALTREQLSNFQAVVFTDISLEKAVEFDDYCHQHQPPIAFIKAEVR 217

NbUBA2b(Nbe18g13930.1) LQELNNAVIISTLTDALTKEHLSNFQAVVFTDISLEKAVEFDDYCHQHQPPIAFIKAEVR 217

NtUBA2a(Nta17g06720.1) LQELNNAVIISTLTDALTKEQLSNFQAVVFTDISLEKAVKFDDYCHQHQPPIAFIKAEVR 217

NtUBA2b(Nta18g08720.1) LQELNNAVIISTLTDALTKEQLSNFQAVVFTDISLEKAVEFDDYCHKHQPPIAFIKAEVR 217

AtUBA2 LQELNNAVAVSTLTGKLTKEQLSDFQVVVFVDISFEKATEIDDYCHSHQPPIAFIKADVR 214

AtUBA1 LQDLNNAVVVSSLTKSLNKEDLSGFQVVVFSDISMERAIEFDDYCHSHQPPIAFVKADVR 217

SlUBA1 LQELNNAVVVSSFTTKLTKEKLSDFQAVVFTNTSLEDALEFNDYCHNHQPPIAFIRTEVR 227

NbUBA1a(Nbe03g13750.1) LQELNNAVAVSTLTRKLTKEKLSDFQAVVFTNTSLEDALEFSDYCHNHQPPIAFIRTEVR 155

NbUBA1b(Nbe04g02160.1) LQELNNAVAVSTLTTKLTKEKLSDFQAVVFTNTSLEDALEFSDYCHNHQPPIAFIRTEVR 231

NtUBA1b(Nta04g02230.2) LQELNNAVAVSTLTTKLNKEKLSDFQAVVFTNTSLEDALEFSGYCHNHQPPIAFIRTEVR 229

NtUBA1a(Nta03g01970.2) LQELNNAVAVSTLTTKLTKEKLSDFQAVVFTNTSLEDALEFSDYCHNHHPPIAFIRTEVR 229

Glyma.02G229700.1 LQELNNAVVVLTLTTKLTKEQLSNFQAVVFTEVSLEKAIEFNDYCHSHQPPIAFIKSEVR 230

Glyma.14G196800.1 LQELNNAVVVLSLTSKLTKEQLSNFQAVVFTEISLEKAIEFNDYCHSHQPPIAFIKSEVR 271

Glyma.11G166100.1 LQELNNAVVVQSLTTQLTKEHLSNFQAVVFTDISLEKACEFNDYCHSHQPHIAFIKTEVR 231

Glyma.18G058900.1 LQELNNAVIVQSLTTQLTKEHLSNFQAVVFTDISLEKAFEFNDYCHSHQPPIAFIKTEVR 249

**:***:* : ::* *. :.** **.*** : .:: * ::..**: * * *:*:::::

XP_015647669.1(LOC_Os07g49230.1) GLFGTVFCDFGPEFTVLDVDGEDPHTGIIASISNDNPALVSCVDDERLEFQDGDFVVFSE 255

XP_015632802.1(LOC_Os03g18380.3) GLFGSVFCDFGPKFTVLDVDGEEPHTGIIASISNDNPAMISCVDDERLEFQDGDLVVFSE 261

ABA95612.2(LOC_Os12g01520.1) GLFGSVFCDFGPEFTVLDVDGEEPHTGIVASISNDNPALVSCVDDERLEFQDGDLVVFSE 246

XP_015616970.1(LOC_Os11g01510.2) GLFGSVFCDFGPEFTVLDVDGEEPHTGIVASISNDNPALVSCVDDERLEFQDGDLVVFSE 245

SlUBA2 GLFGSVFCDFGPDFTVVDVDGEDPHTGIIASISNDNLALVACIDDERLEFQDGDLVIFSE 283

NbUBA2a(Nbe14g09490.1) GLFGSVFCDFGPEFTVADVDGEDPHTGIIASISNDNPALVGCIDDERLEFQDGDLVIFSE 277

NbUBA2b(Nbe18g13930.1) GLFGSVFCDFGPEFTVADVDGEDPHTGIIASISNDNPALVGCIDDERLEFQDGDLVIFSE 277

NtUBA2a(Nta17g06720.1) GLFGSVFCDFGPEFTIADVDGEDPHTGIIASISNDNPALVGCIDDERLEFQDGDLVIFSE 277

NtUBA2b(Nta18g08720.1) GLFGSVFCDFGPEFTVADVDGEDPHTGIIASISNDNPALVGCIDDERLEFQDGDLVIFSE 277

AtUBA2 GLFGSLFCDFGPHFTVLDVDGEEPHSGIIASVSNENPGFVSCVDDERLEFEDGNLVVFSE 274

AtUBA1 GLFGSVFCDFGPEFAVLDVDGEEPHTGIIASISNENQAFISCVDDERLEFEDGDLVVFSE 277

SlUBA1 GLFGYVFCDFGPEFTVFDVDGEEPHTGIIASISNDNPALISCVDDERLEFQDGDLVVFSE 287

NbUBA1a(Nbe03g13750.1) GLFGYVFCDFGPEFTVFDVDGEEPHTGIIASISNDNPALVSCVDDERLEFQDGDLVVFSE 215

NbUBA1b(Nbe04g02160.1) GLFGYAFCDFGPEFTVFDVDGEEPHTGIIASVSNDNPALVSCVDDERLEFQDGDLVVFSE 291

NtUBA1b(Nta04g02230.2) GLFGYVFCDFGPEFTVFDVDGEEPHTSIIASISNDNPALVSCVDDERLEFQDGDLVVFSE 289

NtUBA1a(Nta03g01970.2) GLFGYVFCDFGPEFTVFDVDGEEPHTGIIASISNDNPALVSCVDDERLEFQDGDLVVFSE 289

Glyma.02G229700.1 GLFGSLFCDFGPEFTVVDVDGEDPHTGIIASISNDNPALVSCVDDERLEFQDGDLVVFSE 290

Glyma.14G196800.1 GLFGSLFCDFGPEFTVVDVDGEDPHTGIIASISNDNPALVSCVDDERLEFQDGDLVVFSE 331

Glyma.11G166100.1 GLFGSVFCDFGPEFTVVDVDGEEPRTGIIASINNDNPALVSCVDDERLEFQDGDLVVFSE 291

Glyma.18G058900.1 GLFGSVFCDFGPEFTVVDVDGEEPHTGIIASISNDNPALVSCVDDERLEFQDGDLVVFSE 309

**** ******.*:: *****:*::.*:**:.*:* .::.*:*******:**::*:***

XP_015647669.1(LOC_Os07g49230.1) VHGMAELNDGKPRKVKNARPFSFCIEEDTTKYDMYIKGGIVTQIKEPKILRFKSLRDAMR 315

XP_015632802.1(LOC_Os03g18380.3) VQGMTELNDGKPRKIINARPYSFCIQEDTSKFGIYAKGGIVTQVKEPINLEFKSLRDSIR 321

ABA95612.2(LOC_Os12g01520.1) VHGMSELNDGKPRKIKNARPYSFTLEEDTTSYGTYVRGGIVTQVKPPKVLKFKTLKDAIK 306

XP_015616970.1(LOC_Os11g01510.2) VHGMSELNDGKPRKIKNARPYSFTLEEDTTSYGTYVRGGIVTQVKPPKVLKFKTLKDAIK 305

SlUBA2 VRGMTELNDGKPRKVKCARPYSFTIEDDTTEYKAYERGGIVTQVKEPKVLKFKPLRKAIS 343

NbUBA2a(Nbe14g09490.1) VRGMTELNDGKPRKIKNARPYSFTIEEDTSNYAAYERGGIVTQVKEPKVLKFKPLREAIK 337

NbUBA2b(Nbe18g13930.1) VRGMTELNDGKPRKIKNARPYSFTIEEDTSTYAAYERGGIVTQVKEPKVLKFKPLREAIK 337

NtUBA2a(Nta17g06720.1) VRGMTELNDGKPRKIKNARPYSFTIEEDTSNYAAYERGGIVTQVKEPKVLKFKPLREAIK 337

NtUBA2b(Nta18g08720.1) VRGMTELNDGKPRKIKNARPYSFTIEEDTSNYAAYERGGIVTQVKEPKVLKFKPLREAIK 337

AtUBA2 VEGMTELNDGKPRKIKNVKPFSFTLEEDTSSYGQYMKGGIVTQVKQPKVLNFKPLREALK 334

AtUBA1 VEGMTELNDGKPRKIKSTRPYSFTLDEDTTNYGTYVKGGIVTQVKQPKLLNFKPLREALK 337

SlUBA1 VQGMTELNDGKPRKISSARPYSFTLDEDTTNFGPYVRGGIVTQVKPPKILNFKTLRESIM 347

NbUBA1a(Nbe03g13750.1) VRGMTELNDGKPRKIRSARPYSFTLDDDTTNFGPYERGGIVTQVKQPKLLKFKTLRQAIR 275

NbUBA1b(Nbe04g02160.1) VRGMIELNDGKPRKIRSARPYSFTLEEDTTNFGPYERGGIVTQVKQPKLLKFKTLREAIR 351

NtUBA1b(Nta04g02230.2) VRGMTELNDGKPRKIRSARPYSFTLDEDTTNFGPYERGGIVTQVKQPKLLKFKTLREAIR 349

NtUBA1a(Nta03g01970.2) VRGMTELNDGKPRKIRSARPYSFTLDEDTTNFGPYERGGIVTQVKQPKLLKFKTLSEAIR 349

Glyma.02G229700.1 VHGMEELNDGKPRKIKNARAYSFTLEEDTTNYGRYEKGGIVTQVKQPKVLNFKPLREALS 350

Glyma.14G196800.1 VHGMKELNDGKPRKIKNARAYSFTLEEDTTNYGRYEKGGIVTQVKQPKVLNFKPLREALS 391

Glyma.11G166100.1 IHGMKELNDGKPRKIKNARAYSFTLEEDTTNYGMYEKGGIVTQVKQPKVLNFKPLREALS 351

Glyma.18G058900.1 VHGMKELNDGKPRKIKDARAYSFTLEEDTTNYGTYEKGGIVTQVKQPKVLNFKPLKEAIT 369

:.** *********: .: :** :::**: : * :******:* * *.** * .::

XP_015647669.1(LOC_Os07g49230.1) DPGDFLLSDFSKFERSPVLHLAFQALDKFKKEYGRYPAPGCEQDAQSFLKCAADINEALT 375

XP_015632802.1(LOC_Os03g18380.3) EPGNFLLSDFSKFDRPPLLHFAFLALDKFRKEFGRFPGAGCDQDAQRFIEFVASVNEATI 381

ABA95612.2(LOC_Os12g01520.1) EPGEFLMSDFSKFDRPPLLHLAFQASASFCKWFQ-------------------------- 340

XP_015616970.1(LOC_Os11g01510.2) EPGEFLMSDFSKFDRPPLLHLAFQALDKFRNDLRRFPIAGSSDDVQRLIDFAISINESLG 365

SlUBA2 DPGDFLLSDFSKFDRPPILHLTFQALDKFVSSSGRFPVAGSEEDAQRLISLVTDMNNSQD 403

NbUBA2a(Nbe14g09490.1) DPGDFLLSDFSKFDRPPILHLAFQALDKFVSESGCFPLAGSEEDAQRLISFVTDLNNSLS 397

NbUBA2b(Nbe18g13930.1) DPGDFLLSDFSKFDRPPILHLAFQALDRFVSESGRFPLAGSEEDAQRLISFVTNLNNSLS 397

NtUBA2a(Nta17g06720.1) DPGDFLLSDFSKFDRPPILHLAFQALDRFVSESGRFPLAGSEEDAQRLISFVTDLNNSLS 397

NtUBA2b(Nta18g08720.1) DPGDFLLSDFSKFDRPPILHLAFQALDRFVSESGRFPLAGSEEDAQRLISFVTDLNNSLS 397

AtUBA2 DPGDFLLSDFSKFDRPPLLHLAFQALDRFSSQAGRFPFAGSEEDAQKLVEIAVDINEGLG 394

AtUBA1 DPGDFLFSDFSKFDRPPLLHLAFQALDHFKAEAGRFPVAGSEEDAQKLISIATAINTGQG 397

SlUBA1 DPGDFLLSDFSKFDRPPLLHLAFQALDKFRSDLARFPLAGSEDDAQTLISIATNLNESNG 407

NbUBA1a(Nbe03g13750.1) DPGDFLLSDFSKFERPPLLHLAFQALDKFRSELARFPLAGSEDDAQRLISIAIDLNESMG 335

NbUBA1b(Nbe04g02160.1) DPGDFLLSDFSKFERPPLLHLAFQALDKFRYELARFPLAGSEDDAQRLISIATDLNEGMG 411

NtUBA1b(Nta04g02230.2) DPGDFLLSDFSKFDRPPLLHLAFQALDKFRSELARFPLAGSEDDAQRLITIATDLNESRG 409

NtUBA1a(Nta03g01970.2) DPGDFLLSDFSKFERPPLLHLAFQALDKFRSELARFPLAGSEDDAQRLISIATDLNESMG 409

Glyma.02G229700.1 DPGDFLLSDFSKFDRPPLLHLAFQALDKFVSEIDRFPVAGSEDDAQKLISIASNINGSLG 410

Glyma.14G196800.1 DPGDFLLSDFSKFDRPPLLHLAFQALDKFVSEIGRFPVAGSEDDAQKLISIASNINGSLG 451

Glyma.11G166100.1 DPGDFLLSDFSKFDRPPLLHLAFQALDKFIFELGRFPFAGSEDDALKFISFASYINDSLG 411

Glyma.18G058900.1 DPGDFLLSDFSKFDRPPLLHLAFQALDKFISELGRFPVAGSEDDAQKLISVASHINDSLR 429

:**:**:******:* *:**::* * *

XP_015647669.1(LOC_Os07g49230.1) DHKLDTIDEKLFRHFASGSRAVLNPMAAMFGGIVGQEVVKACSGKFHPLYQFFYFDSVES 435

XP_015632802.1(LOC_Os03g18380.3) DYKMDELDGKLLRNFASGSRAVLNPMAAMFGGIVGQEVVKACSGKFHPQYQFFYFDSAES 441

ABA95612.2(LOC_Os12g01520.1) ----------------------------------------GCSE---------------P 345

XP_015616970.1(LOC_Os11g01510.2) DSKLEELDKKLLHHFASGSRAVLNPMAAMFGGIVGQEVVKACSGKFHPLYQFFYFDSVES 425

SlUBA2 --AKVEIDHELIRNFSFGARAVLNPMAAMFGGIVGQEVVKACSGKFHPLYQFFYFDSVES 461

NbUBA2a(Nbe14g09490.1) DGKLEEIDQKLLRNFAFGARAVLNPMAAMFGGIVAQEVVKACSGKFHPLYQFFYFDSVES 457

NbUBA2b(Nbe18g13930.1) DGKLEEIDQKLLRNFAFGARAVLNPMAAMFGGIVGQEVVKACSGKFHPLYQFFYFDSVES 457

NtUBA2a(Nta17g06720.1) DGKLEEIDQKLLRNFAFGARAVLNPMAAMFGGIVGQEVVKACSGKFHPLYQFFYFDSVES 457

NtUBA2b(Nta18g08720.1) DGKLEEIDQKLLRNFAFGARAVLNPMAAMFGGIVGQEVVKACSGKFHPLYQFFYFDSVES 457

AtUBA2 DARLEDVNSKLLRHLAFGSRAVLNPMAAMFGGIVGQEVVKACSGKFHPIFQFFYFDSVES 454

AtUBA1 DLKVENVDQKLLRHFSFGAKAVLNPMAAMFGGIVGQEVVKACSGKFHPLFQFFYFDSVES 457

SlUBA1 NVKLDDINPKLLQKFSYGARAELNPMAAMFGGIVGQEVVKACSGKFHPLYQFFYFDSLES 467

NbUBA1a(Nbe03g13750.1) EGKLEDINPKLLEQFSSGAGAELNPMAAMFGGIVGQEVVKACSGKFHPLFQFFYFDSVES 395

NbUBA1b(Nbe04g02160.1) EGKLEDINPKLLRQFSSGAGAELNPMAAMFGGIVGQEVVKACSGKFHPLFQFFYFDSVES 471

NtUBA1b(Nta04g02230.2) EGKLEDINPKLLQQFSSGAGAELNPMAAMFGGVVGQEVVKACSGKFHPLFQFFYFDSVES 469

NtUBA1a(Nta03g01970.2) EGKLEDINPKLLQQFSSGAGAELNPMAAMFGGIVGQEVVKACSGKFHPLFQFFYFDSVES 469

Glyma.02G229700.1 DGRLEDVNPKLLQQFAFGARAVLNPMAAMFGGIVGQEVVKACSGKFHPLFQFLYFDSVES 470

Glyma.14G196800.1 DGRLEDVNPKLLQQFSFGARAVLNPMAAMFGGIVGQEVVKACSGKFHPLFQFFYFDSVES 511

Glyma.11G166100.1 DGKLEDINPKLLRYFAFGSRAVLNPMAAVFGGIVGQEVVKACSGKFHPLFQFFYFDSVES 471

Glyma.18G058900.1 DGKLEDINPKLLRYFAFGSRAVLNPMAAMFGGIVGQEVVKACSGKFYPLFQFFYFDSVES 489

.**

XP_015647669.1(LOC_Os07g49230.1) LPTYPLDSEDIKPSNSRYDAQISVFGSKLQKKLEEANTFVVGSGALGCEFLKNLALMGVS 495

XP_015632802.1(LOC_Os03g18380.3) LPTYPLDSKDLKPLNSRYDAQISVFGSKLQKKMRDANVFVVGSGALGCEFLKNLALMGVS 501

ABA95612.2(LOC_Os12g01520.1) YGCNVWWHSELKPENTRYDAQISVFGSNLQKKLEQAKIFMVGSGALGCEFLKNLALMGIS 405

XP_015616970.1(LOC_Os11g01510.2) LPVEPLEPAELKPENTRYDAQISVFGSNLQKKLEQAKIFMVGSGALGCEFLKNLALMGIS 485

SlUBA2 LPTEPLDPNDLKPLNCRYDAQISVFGNKLQQKLEEAKAFVVGSGALGCEFLKNLALMGVC 521

NbUBA2a(Nbe14g09490.1) LPSAPLDPNDLKPLNSRYDAQISVFGNKLQKKLEEAKAFVVGSGALGCEFLKNLALMGVC 517

NbUBA2b(Nbe18g13930.1) LPTAPLDPSDLKPLNSRYDAQISVFGNKLQKKLEEAKAFVVGSGALGCEFLKNLALMGVC 517

NtUBA2a(Nta17g06720.1) LPTAPLDPNDLKPLNSRYDAQISVFGNKLQKKLEEAKAFVVGSGALGCEFLKNLALMGVC 517

NtUBA2b(Nta18g08720.1) LPTAPLDPNDLKPLNSRYDAQISVFGNKLQKKLEEAKAFVVGSGALGCEFLKNLALMGVC 517

AtUBA2 LPKEPLDASEFRPQNSRYDAQISVFGSTLQKKLEDARVFVVGAGALGCEFLKNLALMGVS 514

AtUBA1 LPSEPVDSSDFAPRNSRYDAQISVFGAKFQKKLEDAKVFTVGSGALGCEFLKNLALMGVS 517

SlUBA1 LPTEPLDPSDLKPLNTRYDAQISVFGKKFQKKLEDAKVFMVGSGALGCEFLKNLALMGVA 527

NbUBA1a(Nbe03g13750.1) LPTEPLDPSDLKPLNTRYDAQISVFGHKFQKKLEDAKVFLVGS----------------- 438

NbUBA1b(Nbe04g02160.1) LPTEPLDPSDLKPLNTRYDAQISVFGHSFQKKLEDAKVFLVGSGALGCEFLKNLALMGVS 531

NtUBA1b(Nta04g02230.2) LPTEPLDPSDLKPLNTRYDAQISVFGHKFQKKLEDAKVFLVGSGALGCEFLKNLALMGVS 529

NtUBA1a(Nta03g01970.2) LPTEPLDPSDLKPLNTRYDAQISVFGHKFQKKLEDANVFLVGSGALGCEFLKNLALMGVS 529

Glyma.02G229700.1 LPTEPLDPNDLKPLNSRYDAQISVFGQKLQKKLEDAEVFVVGSGALGCEFLKNLALMGVS 530

Glyma.14G196800.1 LPTEPLDANDLKPLNSRYDAQISVFGQKLQKKLEDAEVFVVGSGALGCEFLKNLALMGVS 571

Glyma.11G166100.1 LPSEPLDPNDFRPVNGRYDAQISVFGHKLQKKLEDSKVFVVGSGALGCEFLKNLALMGVS 531

Glyma.18G058900.1 LPSEPVDPNDFRPVNGRYDAQISVFGQKLQKKLEDSKVFVVGSGALGCEFLKNLALMGVS 549

:: * * ********** .:*:*:.::. * **:

XP_015647669.1(LOC_Os07g49230.1) CSPKGKLTITDDDVIEKSNLSRQFLFRDWNIRQAKSTVAAAAASAINPNLCIDALQNRAC 555

XP_015632802.1(LOC_Os03g18380.3) CGLKGKLTITDDDIIEKSNLSRQFLFRDWNIGQAKSTVAAAAASAINSSLHINALQNRAC 561

ABA95612.2(LOC_Os12g01520.1) CNQNGKLTVTDDDVIEKSNLSRQFLFRDWNIGQPKSTVAATAAMAINPKLHVEALQNRAS 465

XP_015616970.1(LOC_Os11g01510.2) CNQNGKLIVTDDDVIEKSNLSRQFLFRDWNIGQPKSTVAATAAMAINPKLHVEALQNRAS 545

SlUBA2 CGVEGKLTITDDDVIEKSNLSRQFLFRDWNIGQAKSTVAAAAASLINPRIRIEALQNRAS 581

NbUBA2a(Nbe14g09490.1) CGDKGKLIITDDDVIEKSNLSRQFLFRDWNIGQAKSTVAAAAASLINPCIHIEALQNRAS 577

NbUBA2b(Nbe18g13930.1) CGDKGKLTITDDDVIEKSNLSRQFLFRDWNIGQAKSTVAAAAASLINPRIHIEALQNRAS 577

NtUBA2a(Nta17g06720.1) CGDKGKLTITDDDVIEKSNLSRQFLFRDWNIGQAKSTVAAAAASLINPRIHIEALQNRAS 577

NtUBA2b(Nta18g08720.1) CGDKGKLTITDDDVIEKSNLSRQFLFRDWNIGQAKSTVAAAAASLINPRIHIEALQNRAS 577

AtUBA2 CGTQGKLTVTDDDVIEKSNLSRQFLFRDWNIGQAKSTVAATAAAGINSRLNIDALQNRVG 574

AtUBA1 CGSQGKLTVTDDDIIEKSNLSRQFLFRDWNIGQAKSTVAASAAAVINPRFNIEALQNRVG 577

SlUBA1 CTEQGKLTVTDDDVIEKSNLSRQFLFRDWNIGQAKSTVAAAAATSINPQLRVEALQNRVG 587

NbUBA1a(Nbe03g13750.1) --------VTDDDVIEKSNLSRQFLFRDWNIGQAKSTVAATAAASINSQLQVEALQNRVG 490

NbUBA1b(Nbe04g02160.1) CNKQGKLTVTDDDVIEKSNLSRQFLFRDWNIGQAKSTVAATAAASINSQLQVEALQNRVG 591

NtUBA1b(Nta04g02230.2) CNKQGKLTVTDDDVIEKSNLSRQFLFRDWNIGQAKSTVAATAAASINSQLQVEALQNRVG 589

NtUBA1a(Nta03g01970.2) CNKQGKLAVTDDDVIEKSNLSRQFLFRDWNIGQAKSTVAATAAASINSQLQVEALQNRVG 589

Glyma.02G229700.1 CG-QGKLTITDDDVIEKSNLSRQFLFRDWNIGQAKSTVAASAAASINPCLNIDALQNRVG 589

Glyma.14G196800.1 CG-QGKLTITDDDVIEKSNLSRQFLFRDWNIGQAKSTVAASAAASINPRLNIDALQNRVG 630

Glyma.11G166100.1 CGSQGKLTITDDDVIEKSNLSRQFLFRDWNIGQAKSTVAASAAAAINPSFNIEALQNRVG 591

Glyma.18G058900.1 CGSQGKLTITDDDVIEKSNLSRQFLFRDWNIGQAKSTVAASAAAAINPSFNIEALQNRVG 609

:****:***************** * ******:** ** : ::*****.

XP_015647669.1(LOC_Os07g49230.1) PDTENVFHDTFWEGLDVVINALDNVNARMYMDMRCLYFQKALLESGTLGAKCNTQMVIPH 615

XP_015632802.1(LOC_Os03g18380.3) PETEHVFHDKFWEGLDVIINALDNVNARMYMDMRCLYFQKPLLESGTLGPKCNTQMVIPH 621

ABA95612.2(LOC_Os12g01520.1) PETENVFNDAFWESLDAVVNALDNVTARMYIDSRCVYFQKPLLESGTLGAKCNTQMVIPH 525

XP_015616970.1(LOC_Os11g01510.2) PETENVFNDAFWESLDAVVNALDNVTARMYIDSRCVYFQKPLLESGTLGAKCNTQMVIPH 605

SlUBA2 PETESVFDDTFWENLSVVVNALDNVNARLYIDQRCLYFQKPLLESGTLGAKCNTQMVIPH 641

NbUBA2a(Nbe14g09490.1) PETESVFDDTFWENLSVVINALDNVNARLYIDQRCLYFQKPLLESGTLGAKCNTQMVIPH 637

NbUBA2b(Nbe18g13930.1) PETESVFDDTFWENLSVVINALDNVNARLYIDQRCLYFQKPLLESGTLGAKCNTQMVIPH 637

NtUBA2a(Nta17g06720.1) PETESVFDDTFWENLSVVINALDNVNARLYIDQRCLYFQKPLLESGTLGAKCNTQMVIPH 637

NtUBA2b(Nta18g08720.1) PETESVFDDTFWENLSVVINALDNVNARLYIDQRCLYFQKPLLESGTLGAKCNTQMVIPH 637

AtUBA2 PETENVFDDSFWENLTVVVNALDNVTARLYVDSRCVYFQKPLLESGTLGAKCNTQMVIPH 634

AtUBA1 AETENVFDDAFWENLTVVVNALDNVNARLYVDSRCLYFQKPLLESGTLGTKCNTQSVIPH 637

SlUBA1 PETENVFDDTFWENLSVVINALDNVNARLYVDQRCLYFQKPLLESGTLGAKCNTQMVIPH 647

NbUBA1a(Nbe03g13750.1) PETENVFDDTFWENLSVVINALDNIKARLYVDQRCLYFQKPLLESGTLGAKCNTQMVIPH 550

NbUBA1b(Nbe04g02160.1) PETENVFDDTFWENLSVVINALDNINARLYVDQRCLYFQKPLLESGTLGAKCNTQMVIPH 651

NtUBA1b(Nta04g02230.2) PETENVFDDTFWENLSVVINALDNINARLYVDQRCLYFQKPLLESGTLGAKCNTQMVIPH 649

NtUBA1a(Nta03g01970.2) PETENVFDDTFWENLSVVINALDNINARLYVDQRCLYFQKPLLESGTLGAKCNTQMVIPH 649

Glyma.02G229700.1 PETENVFHDTFWENLSVVINALDNVNARLYVDQRCLYFQKPLLESGTLGAKCNTQMVIPH 649

Glyma.14G196800.1 PETENVFHDTFWENLSVVINALDNVNARLYVDQRCLYFQKSLLESGTLGAKCNTQMVIPH 690

Glyma.11G166100.1 SETENVFNDTFWENLSVVVNALDNVNARLYVDQRCLYFQKPLLESGTLGAKCNTQMVIPH 651

Glyma.18G058900.1 TETENVFNDTFWENLSVVVNALDNVNARLYVDQRCLYFQKPLLESGTLGAKCNTQMVIPH 669

:** **.* ***.* .::*****:.**:*:* **:**** ******** ***** ****

XP_015647669.1(LOC_Os07g49230.1) LTENYGASRDPPEKQAPMCTVHSFPHNIDHCLTWARSEFEGLLEKTPGEVNSFLSNPAQY 675

XP_015632802.1(LOC_Os03g18380.3) LTENYGASRDPPEKQAPMCTVHSFPHNIDHCLTWARSEFEGLLEKTPNEVNSFISNPAQY 681

ABA95612.2(LOC_Os12g01520.1) LTENYGASRDPPEKQAPMCTVHSFPHNIDHCLTWARSEFEGLLEKTPTEVNAFLSNPGGY 585

XP_015616970.1(LOC_Os11g01510.2) LTENYGASRDPPEKQAPMCTVHSFPHNIDHCLTWARSEFEGLLEKTPTEVNAFLSNPGGY 665

SlUBA2 LTENYGASRDPPEKQAPMCTVHSFPHNIDHCLTWARSEFEGLLEKTPTEVNAYLINPTDY 701

NbUBA2a(Nbe14g09490.1) LTENYGASRDPPEKQAPMCTVHSFPHNIDHCLTWARSEFEGLLEKTPTEVNAFLINPSDY 697

NbUBA2b(Nbe18g13930.1) LTENYGASRDPPEKQAPMCTVHSFPHNIDHCLTWARSEFEGLLEKTPTEVNAYLINPSDY 697

NtUBA2a(Nta17g06720.1) LTENYGASRDPPEKQAPMCTVHSFPHNIDHCLTWARSEFEGLLEKTPTEVNAYLINPSDY 697

NtUBA2b(Nta18g08720.1) LTENYGASRDPPEKQAPMCTVHSFPHNIDHCLTWARSEFEGLLEKTPTEVNAYLINPSDY 697

AtUBA2 LTENYGASRDPPEKQAPMCTVHSFPHNIDHCLTWARSEFEGLLEKTPAEVNAYLSDPVEY 694

AtUBA1 LTENYGASRDPPEKQAPMCTVHSFPHNIDHCLTWARSEFEGLLEKTPAEVNAYLSSPVEY 697

SlUBA1 LTENYGASRDPPEKQAPMCTLHSFPHNIDHCLTWARSEFEGLLEKTPAEVNAYLSNPNEY 707

NbUBA1a(Nbe03g13750.1) LTENYGASRDPPEKQAPMCTLHSFPHNIDHCLTWARSEFEGLLEKIPAEVNAYLSKPSEY 610

NbUBA1b(Nbe04g02160.1) LTENYGASRDPPEKQAPMCTLHSFPHNIDHCLTWARSEFEGLLEKIPAEVNAYLSNPSEY 711

NtUBA1b(Nta04g02230.2) LTENYGASRDPPEKQAPMCTLHSFPHNIDHCLTWARSEFEGLLEKIPAEVNAYLSNPSEY 709

NtUBA1a(Nta03g01970.2) LTENYGASRDPPEKQAPMCTLHSFPHNIDHCLTWARSEFEGLLEKIPAEVNAYLSNPSEY 709

Glyma.02G229700.1 LTENYGASRDPPEKQAPMCTVHSFPHNIDHCLTWARSEFEGLLEKTPAEVNAYLSNPNEY 709

Glyma.14G196800.1 LTENYGASRDPPEKQAPMCTVHSFPHNIDHCLTWARSEFEGLLEKTPAEVNAYLSNPNEY 750

Glyma.11G166100.1 LTENYGASRDPPEKQAPMCTVHSFPHNIDHCLTWARSEFEGLLEKTPAEVNAYLSNPSEY 711

Glyma.18G058900.1 LTENYGASRDPPEKQAPMCTVHSFPHNIDHCLTWARSEFEGLLEKTPAEVNAYLSNPSEY 729

********************:************************ * ***::: .* *

XP_015647669.1(LOC_Os07g49230.1) AAAMRKAGDAQARELLERVSECLGKERCSLFEDCIRWARLKFEDYFSNRVKQLTFTFPED 735

XP_015632802.1(LOC_Os03g18380.3) AAAMRKAGDAQARELLERVCECLDKERCDGFEDCIAWARLKFEDYFANRVKQLTFTFPED 741

ABA95612.2(LOC_Os12g01520.1) ATVARTAGDAQARDQLERVIECLEREKCETFQDCITWARLKFEDYFSNRVKQLTYTFPED 645

XP_015616970.1(LOC_Os11g01510.2) ATVARTAGDAQARDQLERVIECLEREKCETFQDCITWARLKFEDYFSNRVKQLTYTFPED 725

SlUBA2 ISSMQKAGDAQARDILDRVLECLDKERCDSFEDCITWARLRFEDYFADRVKQLTYTFPED 761

NbUBA2a(Nbe14g09490.1) ISAMQKAGDAQARDTLDRVLECLDKERCDTFQDCITWARLRFEDYFADRVKQLTFTFPEE 757

NbUBA2b(Nbe18g13930.1) ISAMQKAGDAQARDTLDRVLECLDKERCDTFQDCITWARLRFEDYFADRVKQLTFTFPEE 757

NtUBA2a(Nta17g06720.1) ISAMQKAGDAQARDTLDRVLECLDKERCDTFQDCITWARLRFEDYFADRVKQLTFTFPEE 757

NtUBA2b(Nta18g08720.1) ISAMQKAGDAQARDTLDRVLECLDKERCDTFQDCITWARLRFEDYFADRVKQLTFTFPEE 757

AtUBA2 MKAMRTAGDAQARDTLGRVVECLEKEKCNSFQDCITWARLRFEDYFANRVKQLCYTFPED 754

AtUBA1 TNSMMSAGDAQARDTLERIVECLEKEKCETFQDCLTWARLRFEDYFVNRVKQLIYTFPED 757

SlUBA1 TSAQTNAGDAQARDNLERILECLDRESCETFEDCIAWARLKFEEYFANRVKQLIFTFPED 767

NbUBA1a(Nbe03g13750.1) TSAQTNAGDAQARDNLERVLECLDRESCETFEDCIAWARLKFEDYFANRVKQLIFTFPED 670

NbUBA1b(Nbe04g02160.1) TSAQTNAGDAQARDNLERVLECLDRESCETFEDCIAWARLKFEDYFANRVKQLIFTFPED 771

NtUBA1b(Nta04g02230.2) TSAQTNAGDAQARDNLERVLECLDLESCETFEDCIAWARLKFEDYFANRVKQLIFTFPED 769

NtUBA1a(Nta03g01970.2) TSAQTNAGDAQARDNLERVLECLDRESCETFEDCIAWARLKFEDYFANRVKQLIFTFPED 769

Glyma.02G229700.1 TNAMKNAGDAQARDNLERVLECLDKEKCETFEDCITWARLKFEDYFANRVKQLIYTFPED 769

Glyma.14G196800.1 TNAMRNAGDAQARDNLERVLECLDKEKCETFEDCITWARLKFEDYFANRVKQLIYTFPED 810

Glyma.11G166100.1 TNAMKNAGDAQARDNLERVLECLDREKCETFEDCITWARLKFEDYFVNRVKQLIYTFPED 771

Glyma.18G058900.1 TNAMKNAGDAQARDNLERVLECLDQEKCETFEDCITWARLKFEDYFVNRVKQLIYTFPED 789

.*******: * *: *** * *. *:**: ****:**:** :***** :****:

XP_015647669.1(LOC_Os07g49230.1) AATSTGAPFWSAPKRFPRPLQFSVSDPSHIHFIMSASILRAESFGIAIPDWAKNTSKLAD 795

XP_015632802.1(LOC_Os03g18380.3) AVTSTGAFFWSAPKRFPRPLQFSTVNSSHIHFILAASILRAVSFGISIPDWAKNTSNLVD 801

ABA95612.2(LOC_Os12g01520.1) AMTSSGAPFWSAPKRFPRPLEFLTSDPSQLNFILAAAILRAETFGIPIPDWVKNPAKMAE 705

XP_015616970.1(LOC_Os11g01510.2) AMTSSGAPFWSAPKRFPRPLEFLTSDPSQLNFILAAAILRAETFGIPIPDWVKNPAKMAE 785

SlUBA2 AATSSGAPFWSAPKRFPRPLQFSVDDASHLQFLLAASMLRAETFGISIPDWVNSPQKLAE 821

NbUBA2a(Nbe14g09490.1) ATTSSGAPFWSSPKRFPRPLQFSVDDASHLQFLLAASILRAETFGILIPDWVKSPQKLAE 817

NbUBA2b(Nbe18g13930.1) ATTSSGAPFWSAPKRFPRPLQFSVDDASHLQFLLAASILRAETFGILIPDWVKSPQKLAE 817

NtUBA2a(Nta17g06720.1) ATTSSGAPFWSAPKRFPRPLQFSVDDASHLQFLLAASILRAETFGILIPDWVKSPQKLAE 817

NtUBA2b(Nta18g08720.1) ATTSSGAPFWSAPKRFPRPLQFSVDDASHLQFLLAASILRAETFGILIPDWVKSPQKLAE 817

AtUBA2 AATSTGAPFWSAPKRFPRPLQFSSTDLSHINFVMAASILRAETFGIPTPEWAKTRAGLAE 814

AtUBA1 AATSTGAPFWSAPKRFPRPLQYSSSDPSLLNFITATAILRAETFGIPIPEWTKNPKEAAE 817

SlUBA1 AVTSSGAPFWSAPKRFPRPLQFSSTDPSHLHFIMAASILRAETFGIPIPDWVKHPQKLSE 827

NbUBA1a(Nbe03g13750.1) AMTNSGAPFWSAPKRFPHPLQFSSTDPSHLHFIMAASILRAETFGIRIPDWAKHPKKLSE 730

NbUBA1b(Nbe04g02160.1) SMTSSGAPFWSAPKRFPHPLQFSSTDPSHLHFIMAASILRAETFGIPIPDWAKHPKKLNE 831

NtUBA1b(Nta04g02230.2) AMTSSGAPFWSAPKRFPHPLQFSSTDPSHLHFIMAASILRAETFGIPIPDWAKHPKKLSE 829

NtUBA1a(Nta03g01970.2) SMTSSGAPFWSAPKRFPHPLQFSSTDPSHLHFIMAASILRAETFGIPIPDWAKHPKKLSE 829

Glyma.02G229700.1 AATSTGAPFWSAPKRFPHPLQFSSSDLGHLQFLMAASILRAETFGIPIPDWVKNPKKLAE 829

Glyma.14G196800.1 AATSTGAPFWSAPKRFPHPLQFSSSDLGHLLFLMAASILRAETFGIPIPDWVKHPKKLAE 870

Glyma.11G166100.1 AATSTGALFWSAPKRFPRPLQFSATDLGHLYFVLSASILRAETFGIPIPDWGKNPRKMAE 831

Glyma.18G058900.1 AATSTGAPFWSAPKRFPRPLQFSASDLGHLNFVSSASILRAETFGIPIPDWGKNPRKMAE 849

: *.:** ***:*****:**:: : . : *: ::::*** :*** *:* : :

XP_015647669.1(LOC_Os07g49230.1) AVSEVAVPQFEPKKGVSIVTDEKATSLS-SASVDDVSVIDDLLAKLEECAKRLPPGFQMK 854

XP_015632802.1(LOC_Os03g18380.3) AVSKVVVPEFEPKSGVKIETDEKASNIS-SASVDDASVIEDLLTKLEASAKKLPPGFQMK 860

ABA95612.2(LOC_Os12g01520.1) AVDKVIVPDFQPKQGVKIVTDEKATSLS-SASVDDAAVIEELIAKLEAISKTLQPGFQMK 764

XP_015616970.1(LOC_Os11g01510.2) AVDKVIVPDFQPKQGVKIVTDEKATSLS-SASVDDAAVIEELIAKLEAISKTLQPGFQMK 844

SlUBA2 AVDKVMVPDFQPKKDVKIVTDEKATSMS-ASSIDDAAVINELVMQLETCRQKLPSGYKMN 880

NbUBA2a(Nbe14g09490.1) AVDKVIVPDFQPKKDVKIVTDEKATSMA-ASSIDDAAVINELVMKLETCRQKLPSGYKMN 876

NbUBA2b(Nbe18g13930.1) AVDKVMVPDFQPKKDVKIVTDEKATSMA-ASSIDDAAVINELVMKLETCRQKLPSGYKMN 876

NtUBA2a(Nta17g06720.1) AVDKVIVPDFQPKKDVKIVTDEKATSMA-ASSIDDAAVINELVMKLETCRQKLPSGYKMN 876

NtUBA2b(Nta18g08720.1) AVDKVIVPDFQPKKDVKIVTDEKATSMA-ASSIDDAAVINELVMKLETCRQELPSGYKMN 876

AtUBA2 AVERVIVPDFEPKKDATIVTDEKATTLS-TASVDDAAVIDELNAKLVRCRMSLQPEFRMK 873

AtUBA1 AVDRVIVPDFEPRQDAKIVTDEKATTLT-TASVDDAAVIDDLIAKIDQCRHNLSPDFRMK 876

SlUBA1 AVHKVMVPCFQPRKDAKIVTDEKATSLSSSASIDDAAVIDELISKLECGRKNLPPGFRMK 887

NbUBA1a(Nbe03g13750.1) AVDKVMVPDFQPKKDAKIVTDEKETSLS-TASIDDAAVIDELISKLEHYRKNLTPGFRMK 789

NbUBA1b(Nbe04g02160.1) AVDKVMVPDFQPTKDAKIVTDEKATSLS-TASIDDAAVIDELISKLEHYRKNLTPGFRMK 890

NtUBA1b(Nta04g02230.2) AVDKVMVPDFQPKKDAKIVTDEKATSLS-TASIDDAAVIDELISKLEHYRKNLTPGFRMK 888

NtUBA1a(Nta03g01970.2) AVDKVMVPDFQPKKDAKIVTDEKATSLS-TASIDDAAVIDELISKLEHYRKNLTPGFRMK 888

Glyma.02G229700.1 AVDRVIVPDFQPKKDAKIVTDEKATSLS-SASIDDAAVINDLILKLEGCRTKLLPEFRMK 888

Glyma.14G196800.1 AVDRVIVPDFQPKKDAKIVTDEKATSLS-SASIDDAAVINDLIVKLEGCRTKLQPEFRMK 929

Glyma.11G166100.1 AVDRVIVPDFQPKKDVKIVTDEKATSLS-TASIDDAAVINDLVIKLERCRANLSPVFRMK 890

Glyma.18G058900.1 AVDRVIVPDFQPKKDVKIVTDEKATSLS-TASIDDAAVINDLVIKLERCRANLPPVFMMK 908

** .* ** *:* ....* **** :.:: ::*:**.:**::* :: * : *:

XP_015647669.1(LOC_Os07g49230.1) PIQFEKDDDTNFHMDLISGFANMRARNYSIPEVDKLKAKFIAGRIIPAIATSTAMATGLV 914

XP_015632802.1(LOC_Os03g18380.3) AIQFEKDDDTNFHMDLIAGLANMRARNYGIQEVDKLKAKFIAGRIIPAIATSTAMATGLV 920

ABA95612.2(LOC_Os12g01520.1) PIQFEKDDDTNYHMDVIAGFANMRARNYSIPEVDKLKAKFIAGRIIPAIATSTAMATGLV 824

XP_015616970.1(LOC_Os11g01510.2) PIQFEKDDDTNYHMDVIAGFANMRARNYSIPEVDKLKAKFIAGRIIPAIATSTAMATGLV 904

SlUBA2 PIQFEKDDDTNYHMDFIAGLANMRARNYSIPEVDKLKAKFIAGRIIPAIATSTAMATGLV 940

NbUBA2a(Nbe14g09490.1) PIQFEKDDDTNYHMDLIAGLANMRARNYSIPEVDKLKAKFIAGRIIPAIATSTAMATGLV 936

NbUBA2b(Nbe18g13930.1) PIQFEKDDDTNYHMDLIAGLANMRARNYSIPEVDKLKAKFIAGRIIPAIATSTAMATGLV 936

NtUBA2a(Nta17g06720.1) PIQFEKDDDTNYHMDLIAGLANMRARNYSIPEVDKLKAKFIAGRIIPAIATSTAMATGLV 936

NtUBA2b(Nta18g08720.1) PIQFEKDDDTNYHMDLIAGLANMRARNYSIPEVDKLKAKFIAGRIIPAIATSTAMATGLV 936

AtUBA2 AIQFEKDDDTNYHMDMIAGLANMRARNYSVPEVDKLKAKFIAGRIIPAIATSTAMATGFV 933

AtUBA1 PIQFEKDDDTNYHMDVIAGLANMRARNYSIPEVDKLKAKFIAGRIIPAIATSTAMATGLV 936

SlUBA1 PIQFEKDDDTNFHMDLIAALANMRARNYCIPEVDKLKAKFIAGRIIPAIATTTAMATGLV 947

NbUBA1a(Nbe03g13750.1) PIQFEKDDDTNYHMDLIAALANMRARNYSIPEVDKLKAKFIAGRIIPAIATTTAMATGLV 849

NbUBA1b(Nbe04g02160.1) PIQFEKDDDTNYHMDLIAALANMRARNYSIPEVDKLKAKFIAGRIIPAIATTTAMATGLV 950

NtUBA1b(Nta04g02230.2) PIQFEKDDDTNYHMDLIAALANMRARNYSIPEVDKLKAKFIAGRIIPAIATTTAMATGLV 948

NtUBA1a(Nta03g01970.2) PIQFEKDDDTNYHMDLIAALANMRARNYSIPEVDKLKAKFIAGRIIPAIATTTAMATGLV 948

Glyma.02G229700.1 PVQFEKDDDTNYHMDLIAGLANMRARNYSIPEVDKLKAKFIAGRIIPAIATSTAMATGLV 948

Glyma.14G196800.1 PVQFEKDDDTNYHMDLIAGLANMRARNYSIPEVDKLKAKFIAGRIIPAIATSTAMATGLV 989

Glyma.11G166100.1 PIQFEKDDDTNYHMDVIAGLANMRARNYSIPEVDKLKAKFIAGRIIPAIATSTAMATGLV 950

Glyma.18G058900.1 PIQFEKDDDTNYHMDVIAGLANMRARNYSIPEVDKLKAKFIAGRIIPAIATSTAMATGLV 968

:*********:***.*:.:******** : ********************:******:*

XP_015647669.1(LOC_Os07g49230.1) CLELYKVIAGEHPIEDYRNTFANLALPLFSMAEPVPPKVMKHQDMSWTVWDRWSIKGNLT 974

XP_015632802.1(LOC_Os03g18380.3) CLELYKVLAGGHPVEDYRNSFANLAIPMFSMAEPLPPKVIKHQDMRWTIWDRWSIEGNIT 980

ABA95612.2(LOC_Os12g01520.1) CLELYKVLGGGHKVEDYRNTFANLAIPLFSMAEPVPPKTIKHQDMAWTVWDRWTITGNIT 884

XP_015616970.1(LOC_Os11g01510.2) CLELYKVLGGGHKVEDYRNTFANLAIPLFSMAEPVPPKTIKHQDMAWTVWDRWTITGNIT 964

SlUBA2 CLELYKVLNGGHKVEDYRNTFANLALPLFSMAEPVPPKVIKHQDMSWTVWDRWILKDNPT 1000

NbUBA2a(Nbe14g09490.1) CLELYKVLDGGHKVEDYRNTFANLALPLFSMAEPVPPKVTKHQDMNWTVWDRWILKDNPT 996

NbUBA2b(Nbe18g13930.1) CLELYKVLDGGHKVEDYRNTFANLALPLFSMAEPVPPKVVKHQDMNWTVWDRWILKDNPT 996

NtUBA2a(Nta17g06720.1) CLELYKVLDGGHKVEDYRNTFANLALPLFSMAEPVPPKVVKHQDMNWTVWDRWILKDNPT 996

NtUBA2b(Nta18g08720.1) CLELYKVLDGGHKVEDYRNTFANLALPLFSMAEPVPPKVVKHQDMNWTVWDRWILKDNPT 996

AtUBA2 CLEMYKVLDGSHKVEDYRNTFANLALPLFSMAE-VPPKVVKHQDQSWTVWDRWVMRGNPT 992

AtUBA1 CLELYKVLDGGHKVEAYRNTFANLALPLFSMAEPLPPKVVKHRDMAWTVWDRWVLKGNPT 996

SlUBA1 CLELYKVLDGSHKLEDYRNTFANLALPLFSIAEPVPPKIIKHNDLSWTVWDRWVIKDNPT 1007

NbUBA1a(Nbe03g13750.1) CLELYKVLDGGHKLEDYRNTFANLALPLFSMAEPVPPKVVKHQDMSWTVWDRWVIKDNPT 909

NbUBA1b(Nbe04g02160.1) CLELYKVLDGGHKLEDYRNTFANLALPLFSMAEPVPPKVVKHQDMSWTVWDRWVIKDNPT 1010

NtUBA1b(Nta04g02230.2) CLELYKVLDGGHKLEDYRNTFANLALPLFSMAEPVPPKVVKHQDMSWTVWDRWVIKDNPT 1008

NtUBA1a(Nta03g01970.2) CLELYKVLDGGHKLEDYRNTFANLALPLFSMAEPVPPKVVKHQDMSWTVWDRWVIKDNPT 1008

Glyma.02G229700.1 CLELYKALDGGHKVEDYRNTFANLALPLFSMAEPVPPKVIKHQDMSWTVWDRWILKDNPT 1008

Glyma.14G196800.1 CLELYKALDGGHKVEDYRNTFANLALPLFSIAEPVPPKVIKHQDMSWTVWDRWILKDNPT 1049

Glyma.11G166100.1 CLELYKALDGGHKVEDYRNTFANLALPLFSMAEPVPPKIIKHQDMSWTVWDRWILGNNPT 1010

Glyma.18G058900.1 CLELYKVLDGGHKVEDYRNTFANLALPLFSMAEPVPPKIIKHQDMSWTVWDRWILGDNPT 1028

***:**.: * * :* ***:*****:*:**:** :*** **.* **:**** : .* *

XP_015647669.1(LOC_Os07g49230.1) VAELLQWFSDKGLTAYSISCGTSLLYNNMFARHKERLNKKVVDVAREVAKVDVPEYRKHL 1034

XP_015632802.1(LOC_Os03g18380.3) VAELLKWLSDKGLSAYSVSCGTSLLYNTMFPRHKDRVNKKLVDVAKEVAKVDVPAYRRHL 1040

ABA95612.2(LOC_Os12g01520.1) LRELLDWLKEKGLNAYSISCGTSLLYNSMFPRHKERLDKKVVDVAREVAKVEVPPYRRHL 944

XP_015616970.1(LOC_Os11g01510.2) LRELLDWLKEKGLNAYSISCGTSLLYNSMFPRHKERLDKKVVDVAREVAKVEVPPYRRHL 1024

SlUBA2 LRELLQWLQNKGLNAYSISYGSCLLYNSMFPKHKERMDRKMVELAKEVAKADLPPYRKHF 1060

NbUBA2a(Nbe14g09490.1) LRELLQWLQNKGLNAYSISYGSCLLYNSMFPKHKERMDRKLVDLAREVAKADLPPYRKHF 1056

NbUBA2b(Nbe18g13930.1) LRELLQWLQNKGLNAYSISYGSCLLYNSMFPKHKERMDRKMVDLAREVAKADLPPYRKHF 1056

NtUBA2a(Nta17g06720.1) LRELLQWLQNKGLNAYSISYGSCLLYNSMFPKHKERMDRKLVDLAREVAKADLPPYRKHF 1056

NtUBA2b(Nta18g08720.1) LRELLQWLQNKGLNAYSISYGSCLLYNSMFPKHKERMDRKLVDLAREVAKADLPPYRKHF 1056

AtUBA2 LRELLDWLKEKGLNAYSISCGSSLLYNSMFSRHKERMNRRVVDLARDVAGVELPAYRRHV 1052

AtUBA1 LREVLQWLEDKGLSAYSISCGSCLLFNSMFTRHKERMDKKVVDLARDVAKVELPPYRNHL 1056

SlUBA1 LRELIQWLADKGLNAYSISCGSCLLFNSMFPRHKERMDQKVVDLARDVAKMEIPPYRRHL 1067

NbUBA1a(Nbe03g13750.1) LRELIQWLADKGLDAYSISCGSCLLFNSMFPRHKERMDLKVVDLARDVAKVELPPYRRHL 969

NbUBA1b(Nbe04g02160.1) LRELIQWLADRGLNAYSISCGSCLLFNSMFPRHKERMDKKVVDLARDVAKVELPPYRRHL 1070

NtUBA1b(Nta04g02230.2) LRELIQWLADKGLNAYSISCGSCLLFNSMFPRHKERMDKKVVDLARDVAKVELPPYRRHL 1068

NtUBA1a(Nta03g01970.2) LRELIQWLADKGLNAYSISCGSCLLFNSMFPRHKERMDKRVVDLARDVAKVELPPYRRHL 1068

Glyma.02G229700.1 LRELLEWLKSKGLNAYSISCGSCLLYNSMFPRHRERMDKKMVDLAREVAKVEIPSYRRHL 1068

Glyma.14G196800.1 LRELLEWLKAKGLNAYSISCGSCLLYNSMFPRHRERMDKKMVDLAREVAKVEIPSYRRHL 1109

Glyma.11G166100.1 LRELLEWLKAKGLNAYSISCGSCLLYNSMFPRHKDRMDKKVADLARDVAKLEIPSYRRHL 1070

Glyma.18G058900.1 LRELLEWLKAKGLNAYSISCGSCLLYNSMFPRHKDRMDKKVADLAREVAKFEILAYRRHL 1088

: *::.*: :** ***:* *:.**:*.** :*::*:: ::.::*::** :: **.*.

XP_015647669.1(LOC_Os07g49230.1) DLVAACEDDDGNDIDIPLVSVYFR 1058

XP_015632802.1(LOC_Os03g18380.3) DVVVACEDDDGNDVDIPLISIYFR 1064

ABA95612.2(LOC_Os12g01520.1) DVVVACEDDDDNDVDIPLVSIYFR 968

XP_015616970.1(LOC_Os11g01510.2) DVVVACEDDDDNDVDIPLVSIYFR 1048

SlUBA2 DVVVACEDDEDNDVDIPQVSIYFR 1084

NbUBA2a(Nbe14g09490.1) DVVVACEDEEDNDVDIPQMSIYFR 1080

NbUBA2b(Nbe18g13930.1) DVVVACEDEEDNDVDIPQMSIYFR 1080

NtUBA2a(Nta17g06720.1) DVVVACEDEEDNDVDIPQMSIYFR 1080

NtUBA2b(Nta18g08720.1) DVVVACEDEEDNDVDIPQMSIYFR 1080

AtUBA2 DVVVACEDDNDADVDIPLVSVYFA 1076

AtUBA1 DVVVACEDEDDNDVDIPLVSIYFR 1080

SlUBA1 DVVVACDDDNDEDVDIPLVSVYFR 1091

NbUBA1a(Nbe03g13750.1) DVVVACEDDEDNDVDIPLMSVYFR 993

NbUBA1b(Nbe04g02160.1) DVVVACEDDEDNDVDIPLVSVYYR 1094

NtUBA1b(Nta04g02230.2) DVVVACEDDEDNDVDIPLVSVYFR 1092

NtUBA1a(Nta03g01970.2) DVVVACEDDEDNDVDIPLVSVYFR 1092

Glyma.02G229700.1 DVVVACEDDDDNDIDIPQISIYFR 1092

Glyma.14G196800.1 DVVVACEDDEDNDIDIPQISIYFR 1133

Glyma.11G166100.1 DVVVACEDDEDNDIDIPQISVYFR 1094

Glyma.18G058900.1 DVVVACEDDEDNDIDIPQISIYFR 1112

*:*.**:*::. *:*** :*:*:
