# Supplementary material for: The Two Tomato Ubiquitin E1 Enzymes Play Unequal Roles in Host Immunity
Source: Mol Plant Pathol. 2025 Sep 29;26(10):e70160. doi: 10.1111/mpp.70160 (PMC12477439; doi:10.1111/mpp.70160)
Supplement: Supplementary file 2 — Data S2:Detailed description of experimental procedures for some experiments. [file MPP-26-e70160-s013.docx]

**Supplementary Methods**

**DNA Manipulations and Plasmid Constructions**

Standard molecular biology techniques were employed for DNA manipulations (Green *et al.*, 2012). The opening reading frame (ORF) of the E1 and E2 genes of tomato, *Nicotiana benthamiana*, and Arabidopsis, as well as fragments of the tomato E1 genes for virus-induced gene silencing of tomato and *N. benthamiana* E1 genes were amplified from tomato cDNA or plasmid DNA using the Q5 High-Fidelity DNA Polymerase (New England Biolabs) and sequence confirmed. The chimeric SlUBA1-Ufd^SlUBA2^ and SlUBA2-Ufd^SlUBA1^ were cloned by overlapping PCR of N-terminus fragment and the Ufd domains (Ufd^SlUBA1^ and Ufd^SlUBA2^). All cloned genes and VIGS fragments were inserted into the pENTR/SD/D-TOPO entry vector by Gateway cloning according to the protocols provided by the manufacturer (Invitrogen Life Technologies), followed by construction into the appropriate expression vectors. For BiFC assay, the genes were constructed into BiFC vectors through the digestions and ligations (pSPYNE173 and pSPYCE(M). The pDEST15 and pDEST17 vectors were used for building constructs that express GST-tagged and 6HIS-tagged proteins, respectively (Invitrogen Life Science Technologies).

**Sequence Alignment and Phylogenetic Analysis**

For sequence alignment, sequences of interest in the FASTA format were entered into the Clustal Omega program and aligned using the Clustal Omega algorithm (Sievers *et al.*, 2011). The phylogenetic analysis was then performed with the MEGAX program using the aligned sequences (Tamura *et al.*, 2021). To build an unrooted phylogenetic tree using MEGAX, the evolutionary history was inferred using the neighbor-joining method with 500 bootstrap trials. The evolutionary distances were computed using the p-distance method in which the evolutionary distance unit represents the number of amino acid (or nucleotide) substitutions per site (Nei & Kumar, 2000). Branches corresponding to partitions reproduced <50% bootstrap replicates were collapsed in the tree.

**Examination of Charging Ubiquitin E2s by E1s via Thioester Assay**

To examine the efficiencies of charging E2s by E1s, the thioester assay was performed as described with modifications (Mural *et al.*, 2013). In a 15 µL reaction, 40 ng of ubiquitin E1 (tomato E1 GST-SlUBA1, GST-SlUBA2, tomato chimeric E1 GST-SlUBA1-UFD^SlUBA2^, GST-SlUBA2-UFD^SlUBA1^, GST-SlUBA2^Q1009A^, GST-SlUBA2^Q1009K^, Arabidopsis GST-AtUBA1, and GST-AtUBA2, respectively) was pre-incubated with 2.0 μg of FLAG-ubiquitin in 20 mM Tris-HCl pH 7.5, 10 mM MgCl_2_, and 1 mM ATP at 28 °C for 10 min, which is followed by adding 100 ng of the GST or 6HIS - fused wild type or mutated E2 protein to be tested. The reaction was then continued for 15 min before being stopped with SDS sample loading buffer (62.5 mM Tris-HCl pH 6.8, 2% SDS, 0.01% bromophenol blue, 10% glycerol and 4M Urea). To test the DTT sensitivity of E2-ubiquitin linkage in the thioester assay, the reaction volume was scaled up to 20 µL. The reaction was then equally split and terminated by addition of SDS sample loading buffer with either 100 mM dithiothreitol (DTT+) or 4 M urea sample buffer (DTT-). The reactions were immunoblotted with mouse monoclonal anti-FLAG M2-peroxidase-conjugated antibody (Sigma-Aldrich) before being detected using ECL kit (Pierce, now Thermo Fisher). The formation of DTT-sensitive ubiquitin adducts to tomato E2 SlUBC3 is denoted as charged E2. To examine the effect of knocking down *NbUBA1a/1b* or *NbUBA2a/2b* on the charging of SlUBC32, SlUBC33, and SlUBC34 *in planta*, *Agrobacterium*-mediated transient expression was employed to express 10Myc-tagged E2s in the leaves of *N. benthamiana* plants where *NbUBA1a/1b* or *NbUBA2a/2b* were silenced. Plant proteins were extracted using extraction buffer without DTT and approximately 50 μg of total proteins was used for immunoblotting.

**Yeast Two-Hybrid Assays**

For testing the interaction of two proteins using the LexA-based yeast two-hybrid system, procedures were followed as described(Golemis *et al.*, 2008). In brief, *Ufd^SlUBA1^*, *Ufd^SlUBA2^*, *SlUBA1* and *SlUBA2* were introduced into the bait Gateway destination vector pNLexAattR containing the LexA DNA binding domain as well as a nuclear localization signal. *SlUBC32*, *SlUBC33*, *SlUBC34*, were introduced into the prey Gateway destination vector pJZ4attR, respectively, which contains the activation domain and a Gal-inducible promoter (Prasad *et al.*, 2010). The bait and prey vectors were first transformed into yeast strain EGY48 carrying the reporter plasmid pSH18-34 using the lithium acetate/polyethylene glycol method as described in the Yeast Protocols Handbook (Clontech). Transformed yeast were selected and maintained on dropout medium (Sunrise Science Product) plus Glc but without uracil, His, and Trp. To detect an interaction, yeast colonies containing the bait and prey plasmids were streaked onto medium containing Gal/raffinose and 5-bromo-4-chloro-3-indoyl-β-d-galactopyranoside (BioShop, Canada) but lacking uracil, His, and Trp. Binding of activation domain fusion proteins to binding domain fusion proteins was detected by the presence of a blue color produced by the metabolism of 5-bromo-4-chloro-3-indoyl-β-d-galactopyranoside by β-galactosidase. Expression of fusion proteins was verified by immunoblot using either mouse anti-LexA (Dualsystems Biotech) or rat anti-HA horseradish peroxidase–conjugated (Roche) monoclonal antibodies.

**Bimolecular fluorescence complementation (BiFC) Assay**

The BiFC assay that is based on split yellow fluorescent protein (YFP) was used to test the interaction of various E1-E2 pairs in the leaves and protoplasts (Chen et al., 2006; Waadt et al., 2008). For the assay using *N. benthamiana* leaves, N-terminal YFP-fused and C-terminal YFP-fused proteins were transiently coexpressed in the leaves of *N. benthamiana* plants. N-terminal YFP-fused and C-terminal GUS proteins or empty vectors were used as negative controls. The leaves were imaged at 48 h after infection. For the assay using protoplasts, the empty vectors expressing the N terminus and C terminus of YFP (nYFP-EV and cYFP-EV) were used as negative controls. Protoplasts were prepared from leaves of tomato and *N. benthamiana* plants as described (Rosebrock et al., 2007). Approximately 1 × 10^4^ protoplasts that were suspended in a volume of 200 μL were then cotransfected with 10 µg of plasmid DNA of each individual of the construct pair to be tested. The cotransfected protoplast was imaged 21 h after transfection using an Olympus FV500 Inverted (Olympus IX-81) confocal microscope with the following excitation and emission wavelengths: YFP, 514.5 nm (excitation) and 525 to 555 nm (emission); chlorophyll autofluorescence, 640.5 nm (excitation) and 663 to 738 nm (emission).

**Coimmunoprecipitation**

The coimmunoprecipitation assay of HA-tagged E1s and FLAG-tagged E2s was performed as described previously with some modifications (Zhou *et al.*, 2017)(Moffett et al., 2002). Protein extracts from *Agro*-infiltrated *N. benthamiana* leaves were prepared by grinding 0.8 g of leaf tissue in 1.5 mL of extraction buffer (25 mm Tris-HCl, pH 7.5, 1 mm EDTA, 150 mm NaCl, 10% glycerol, 1 mm DTT, and 0.15% Nonidet P-40) in the presence of plant protease inhibitor cocktail (Sigma-Aldrich). Extracts were spun for 5 min at 12,000 rpm two times, and the supernatant was added to 25 µL anti-HA (3F10) agarose beads (Roche). Extracts were incubated with shaking at 4°C for 3 h followed by washing three times with the washing buffer (25 mm Tris-HCl, pH 7.5, 1 mm EDTA, 150 mm NaCl, 10% glycerol, 0.15% Nonidet P-40, and 1× protease inhibitor cocktail), and the pellet was resuspended in 100 μL of 1× SDS-PAGE loading buffer. Immunoprecipitated samples were separated by 10% SDS-PAGE and analyzed by immunoblot using anti-HA and anti-FLAG antibodies (Santa Cruz).

**Virus-Induced Gene Silencing (VIGS)**

Gene silencing was induced using the *N. benthamiana* rattle virus (TRV) vectors as previously described (Mural et al., 2013). *Agrobacterium* (OD600 = 0.5) containing appropriate pTRV plasmids was induced with acetosyringone and used to infiltrate two leaf-stage tomato seedlings and 3-week-old *N. benthamiana* seedlings. VIGS-treated tomato plants were maintained for 3 to 4 weeks at 21°C /21°C, 16/8 h day/night condition, whereas VIGS-treated *N. benthamiana* plants were maintained for 3 to 4 weeks at 24°C /22°C, 16/8 h day/night condition to allow silencing to occur.

**Bacterial Population Assay**

The bacterial population assay was conducted as described previously (Nguyen *et al.*, 2010). Briefly, for assaying the DC3000Δ*hopQ1-1* growth, *N. benthamiana* plants about four weeks after VIGS infection were first vacuum infiltrated with *P. fluorescens* 55 (*P. flu*55) by submersion of the aerial parts of the plant in a suspension of *P. flu*55 (5 × 10^7^ CFU/mL) containing 0.002% Silwet L-77 and 10 mM MgCl_2_. The plants were then inoculated with *Pst* DC3000Δ*hopQ1-1* (2 × 10^5^ CFU/mL) in the presence of 0.002% Silwet L-77 and 10 mM MgCl_2_ by vacuum infiltration 7 h after the treatment with *P. flu*55. For assaying the growth of *Pst* strains DC3000 and DC3000*ΔhrcQ-U*, about four-week-old tomato plants about four weeks after VIGS infection were inoculated with the suspension of pathogen DC3000*ΔhrcQ-U* (1 × 10^9^ CFU/mL) containing 0.002% Silwet L-77 and 10 mM MgCl_2_ by vacuum infiltration. Inoculated plants were maintained in a growth chamber and monitored daily for symptom development. To assess bacterial populations, leaf discs were harvested from three to four plants of each treatment on day 3 and day 4 after the inoculation and ground, serially diluted, and plated to determine the amount of the bacteria grown as described (Zhou et al., 2017).

**Measurement of plant leaf area**

Plant leaves were photographed vertically from above using a Nikon D3300 camera, with a ruler placed alongside the leaves against a black cloth background. Leaf areas were measured using ImageJ software following its user manual. The protocol included the following steps: (1) Open the image in ImageJ (File > Open); (2) Set the scale by drawing a line along a 1.0-inch ruler using the straight line tool, then selecting Analyze > Set Scale, entering 1.0 in "Known Distance," setting the unit to "inches," and checking "Global" to apply the scale; (3) Convert the image to 8-bit (Image > Type > 8-bit) for thresholding; (4) Adjust the threshold (Image > Adjust > Threshold) to isolate green leaf areas using Hue, Saturation, and Brightness sliders, then click "Apply" to create a binary image; (5) Outline each leaf using the freehand selection tool and measure its area in square inches (Analyze > Measure); (6) Repeat for all leaves. Leaf areas were recorded to three decimal places, and statistical analysis was performed using the Tukey-Kramer HSD test. At least eight leaves from a minimum of four plants per treatment were analyzed.

**References**

Golemis, E. A., Serebriiskii, I., Finley Jr, R. L., Kolonin, M. G., Gyuris, J. and Brent, R. (2008) Interaction trap/two-hybrid system to identify interacting proteins. In: *Current Protocol in Molecular Biology.* (Ausubel, F. M., Brent, R., Kingston, R. E., Moore, D. D., Seidman, J. G., Smith, J. A.*, et al.*, eds.). New York: John Wiley, pp. 20.21.21-20.21.35.

Green, M. R., Sambrook, J. and Sambrook, J. (2012) *Molecular cloning : a laboratory manual*. Cold Spring Harbor, N.Y.: Cold Spring Harbor Laboratory Press.

Mural, R. V., Liu, Y., Rosebrock, T. R., Brady, J. J., Hamera, S., Connor, R. A.*, et al.* (2013) The tomato Fni3 lysine-63-specific ubiquitin-conjugating enzyme and suv ubiquitin E2 variant positively regulate plant immunity. *Plant Cell,* **25,** 3615-3631.

Nei, M. and Kumar, S. (2000) *Molecular evolution and phylogenetics*. Oxford ; New York: Oxford University Press.

Nguyen, H. P., Chakravarthy, S., Velasquez, A. C., McLane, H. L., Zeng, L., Nakayashiki, H.*, et al.* (2010) Methods to study PAMP-triggered immunity using tomato and Nicotiana benthamiana. *Mol. Plant. Microbe Interact.,* **23,** 991-999.

Prasad, M. E., Schofield, A., Lyzenga, W., Liu, H. and Stone, S. L. (2010) Arabidopsis RING E3 ligase XBAT32 regulates lateral root production through its role in ethylene biosynthesis. *Plant Physiol.,* **153,** 1587-1596.

Sievers, F., Wilm, A., Dineen, D., Gibson, T. J., Karplus, K., Li, W.*, et al.* (2011) Fast, scalable generation of high-quality protein multiple sequence alignments using Clustal Omega. *Mol. Syst. Biol.,* **7,** 539.

Tamura, K., Stecher, G. and Kumar, S. (2021) MEGA11: Molecular Evolutionary Genetics Analysis Version 11. *Mol Biol Evol,* **38,** 3022-3027.

Zhou, B., Mural, R. V., Chen, X., Oates, M. E., Connor, R. A., Martin, G. B.*, et al.* (2017) A Subset of Ubiquitin-Conjugating Enzymes Is Essential for Plant Immunity. *Plant Physiol.,* **173,** 1371-1390.
