# Supplementary material for: The Two Tomato Ubiquitin E1 Enzymes Play Unequal Roles in Host Immunity
Source: Mol Plant Pathol. 2025 Sep 29;26(10):e70160. doi: 10.1111/mpp.70160 (PMC12477439; doi:10.1111/mpp.70160)
Supplement: Supplementary file 3 — Figure S1: Tomato E1 proteins SlUBA1 and SlUBA2 possess typical domain organisation of ubiquitin E1 enzymes. [file MPP-26-e70160-s001.pdf]

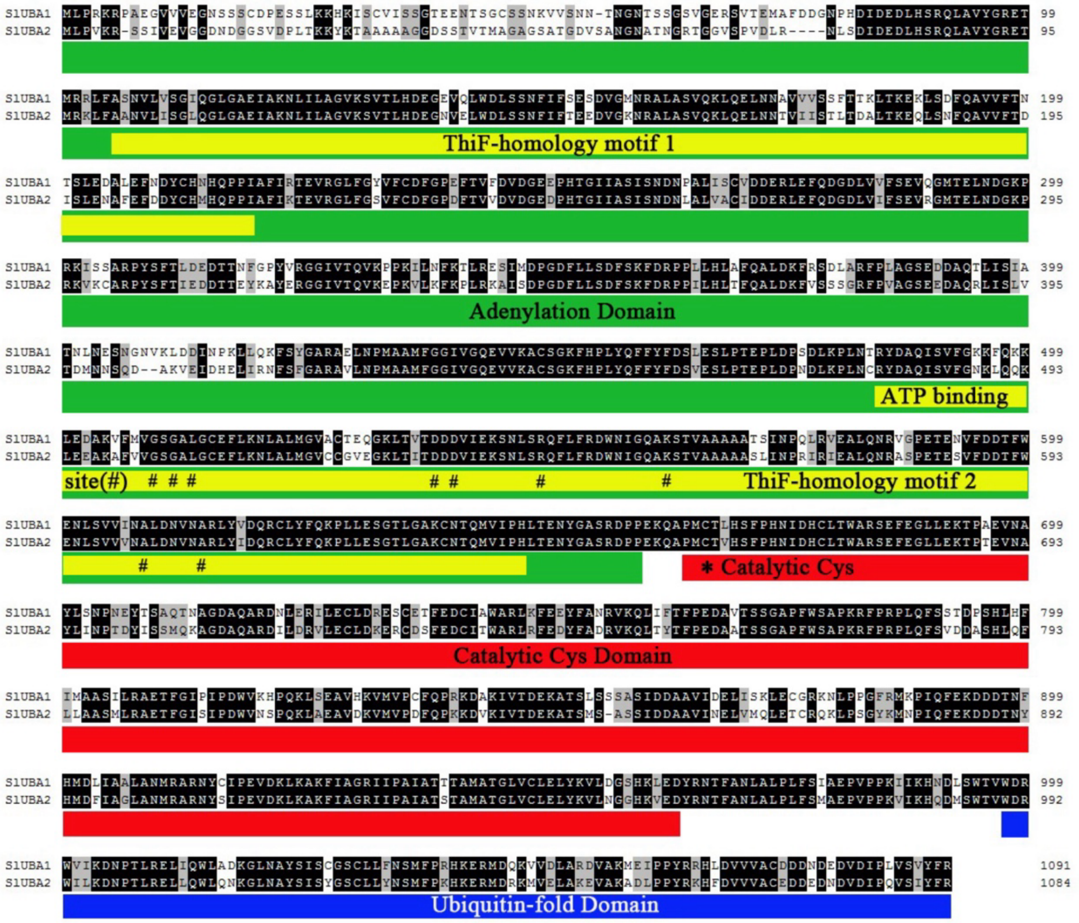

Supplementary Figure 1. Tomato E1 proteins SlUBA1 and SlUBA2 possess typical domain organization of ubiquitin E1 enzymes.

The amino acids spanning the adenylate domain is dark green underlined; the amino acids constitute the two ThiF motifs are yellow underlined; Red underline denotes the catalytic cysteine domain (CCD); and blue underline marks the ubiquitin-fold domain (UFD). The SlUBA1 protein has 1091 amino acid residues and shares 76.3% and 74.1% identity with AtUBA1 and AtUBA2, respectively. Meanwhile, SlUBA2 possesses 1084 amino acid residues and shares 73.7% identity with AtUBA1 and 72.8% identity with AtUBA2.
