# Supplementary material for: The Two Tomato Ubiquitin E1 Enzymes Play Unequal Roles in Host Immunity
Source: Mol Plant Pathol. 2025 Sep 29;26(10):e70160. doi: 10.1111/mpp.70160 (PMC12477439; doi:10.1111/mpp.70160)
Supplement: Supplementary file 4 — Figure S2: Phylogenetic analysis of ubiquitin E1 proteins from Arabidopsis, tomato, N. benthamiana, rice and human. [file MPP-26-e70160-s005.pdf]

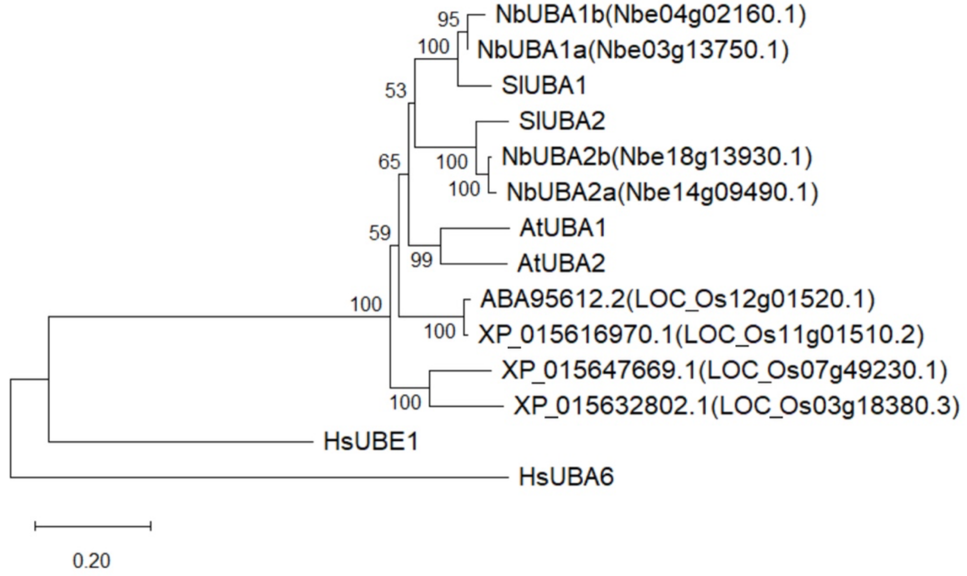

Supplementary Figure 2. The tomato E1s SlUBA1 and SlUBA2 show highest homology to the *Nicotiana benthamiana* E1s.

An unrooted phylogenetic tree of the E1 enzymes from tomato, *N. benthamiana(Wang et al., 2024)*, *N. tabacum(Wang et al., 2024)*, Arabidopsis(Zhao *et al.*, 2013), rice(Hamilton *et al.*, 2024), and human. The Human E1 HsUBA6 is included as an outsider. Evolutionary analyses were conducted using MEGA12 (Kumar *et al.*, 2024) with 1,000 bootstrap trials(Tamura *et al.*, 2013). The evolutionary history was inferred using the Neighbor-Joining method(Saitou & Nei, 1987). The optimal tree with the sum of branch length = 3.140 is shown. The tree is drawn to scale, with branch lengths in the same units as those of the evolutionary distances used to infer the phylogenetic tree. The evolutionary distances were computed using the JTT matrix-based method (Jones *et al.*, 1992) and are in the units of the number of amino acid substitutions per site. The rate variation among sites was modeled with a gamma distribution (shape parameter = 1).

Reference

Hamilton, John P., Li, C. and Buell, C. R. (2024) The rice genome annotation project: an updated database for mining the rice genome. *Nucleic Acids Res.,* **53,** D1614–D1622.

Jones, D. T., Taylor, W. R. and Thornton, J. M. (1992) The rapid generation of mutation data matrices from protein sequences. *Comput. Appl. Biosci.,* **8,** 275–282.

Kumar, S., Stecher, G., Suleski, M., Sanderford, M., Sharma, S. and Tamura, K. (2024) MEGA12: Molecular Evolutionary Genetic Analysis Version 12 for Adaptive and Green Computing. *Molecular Biology and Evolution,* **41**.

Saitou, N. and Nei, M. (1987) The neighbor-joining method: a new method for reconstructing phylogenetic trees. *Mol Biol Evol,* **4,** 406–425.

Tamura, K., Stecher, G., Peterson, D., Filipski, A. and Kumar, S. (2013) MEGA6: Molecular Evolutionary Genetics Analysis Version 6.0. *Molecular Biology and Evolution,* **30,** 2725–2729.

Wang, J., Zhang, Q., Tung, J., Zhang, X., Liu, D., Deng, Y.*, et al.* (2024) High-quality assembled and annotated genomes of Nicotiana tabacum and Nicotiana benthamiana reveal chromosome evolution and changes in defense arsenals. *Mol Plant,* **17,** 423–437.

Zhao, Q., Tian, M., Li, Q., Cui, F., Liu, L., Yin, B.*, et al.* (2013) A plant-specific in vitro ubiquitination analysis system. *Plant J.,* **74,** 524–533.
