# Supplementary material for: The Two Tomato Ubiquitin E1 Enzymes Play Unequal Roles in Host Immunity
Source: Mol Plant Pathol. 2025 Sep 29;26(10):e70160. doi: 10.1111/mpp.70160 (PMC12477439; doi:10.1111/mpp.70160)
Supplement: Supplementary file 6 — Figure S4: DNA sequence alignment of tomato SlUBA2 and N. benthamiana E1 genes NbUBA2a and NbUBA2b. [file MPP-26-e70160-s014.pdf]

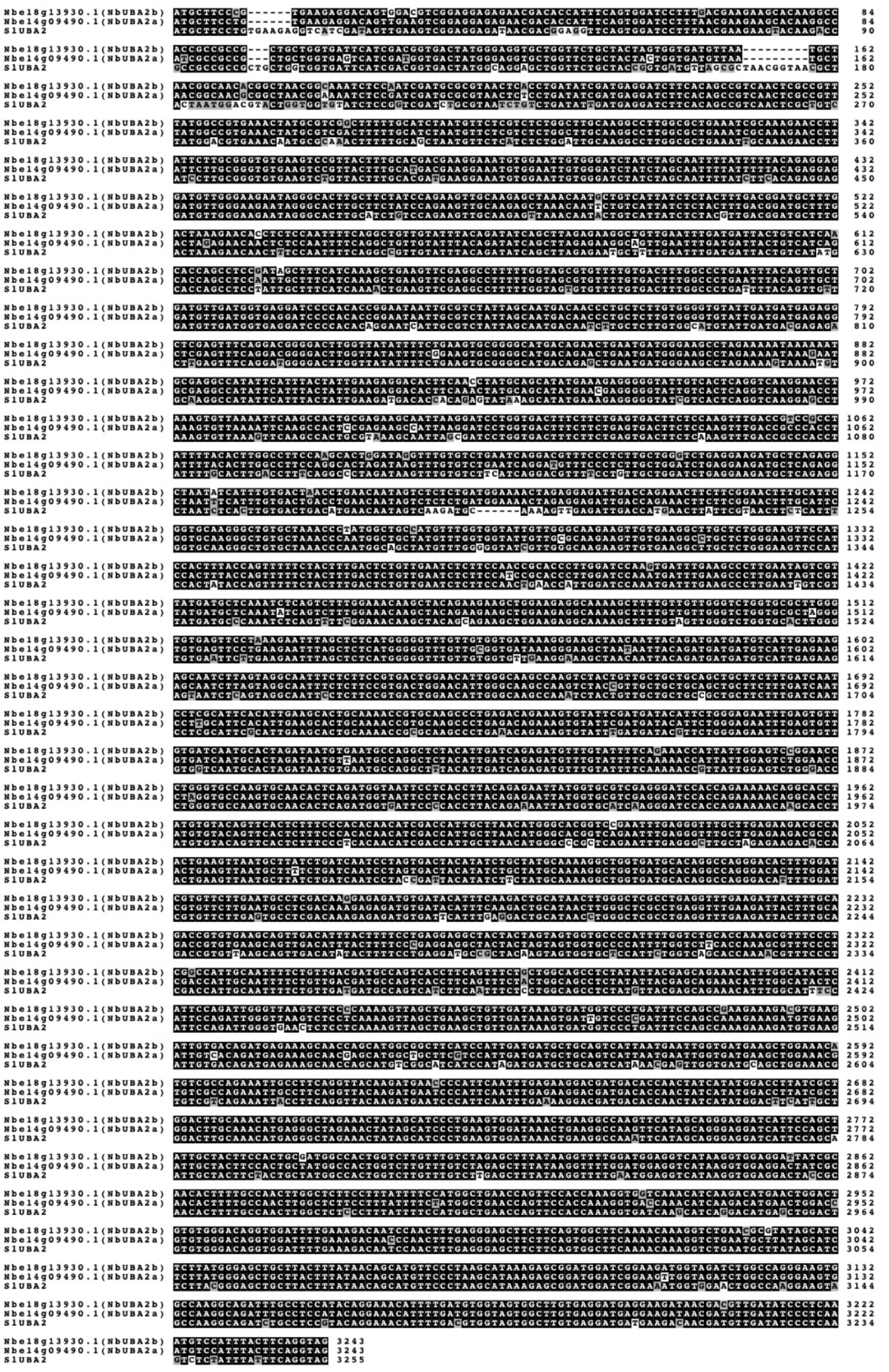

Supplementary Figure 4. **DNA sequence alignment of tomato *SlUBA2* and *N. benthamiana* E1 genes *NbUBA2a* and *NbUBA2b.*** *NbUBA2a* and *NbUBA2b* are 90.86% and 90.89% identical to *SlUBA2* in DNA sequence, respectively. The sequences were aligned using Clustal Omega algorithm with default parameters (Sievers *et al.*, 2011).

Reference

Sievers, F., Wilm, A., Dineen, D., Gibson, T. J., Karplus, K., Li, W., *et al.* (2011) Fast, scalable generation of high-quality protein multiple sequence alignments using Clustal Omega. *Mol. Syst. Biol.,* **7,** 539.
