# Supplementary material for: The Two Tomato Ubiquitin E1 Enzymes Play Unequal Roles in Host Immunity
Source: Mol Plant Pathol. 2025 Sep 29;26(10):e70160. doi: 10.1111/mpp.70160 (PMC12477439; doi:10.1111/mpp.70160)
Supplement: Supplementary file 7 — Figure S5: DNA sequence alignment of tomato SlUBA1 and SlUBA2 gene fragments used for virus‐induced gene silencing (VIGS) of SlUBA1/NbUBA1a/1b and SlUBA2/NbUBA2a/2b, respectively and corresponding regions of NbUBA1a/1b and NbUBA2a/2b genes. [file MPP-26-e70160-s010.pdf]

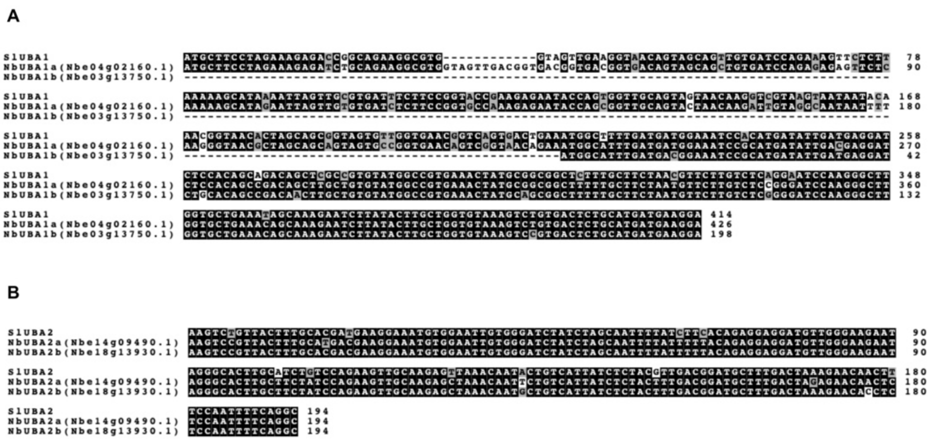

Supplementary Figure 5. DNA sequence alignment of tomato *SlUBA1* and *SlUBA2* gene fragments used for virus-induced gene silencing (VIGS) of *SlUBA1/NbUBA1a/1b* and *SlUBA2/NbUBA2a/2b*, respectively and corresponding regions of *NbUBA1a/1b* and *NbUBA2a/2b* genes. (**A**) DNA sequence alignments of tomato *SlUBA1* gene fragments used for VIGS and corresponding region of *NbUBA1a* and *NbUBA1b*. (**B**) DNA sequence alignments of tomato *SlUBA2* gene fragments used for VIGS and corresponding region of *NbUBA2a* and *NbUBA2b*. Identical DNA sequences of the three genes examined are highlighted in black.
