# Supplementary material for: The Two Tomato Ubiquitin E1 Enzymes Play Unequal Roles in Host Immunity
Source: Mol Plant Pathol. 2025 Sep 29;26(10):e70160. doi: 10.1111/mpp.70160 (PMC12477439; doi:10.1111/mpp.70160)
Supplement: Supplementary file 8 — Figure S6: The DNA fragments used for VIGS of SlUBA1/NbUBA1a/1b and SlUBA2 NbUBA2a/2b, respectively share minimal homology. [file MPP-26-e70160-s009.pdf]

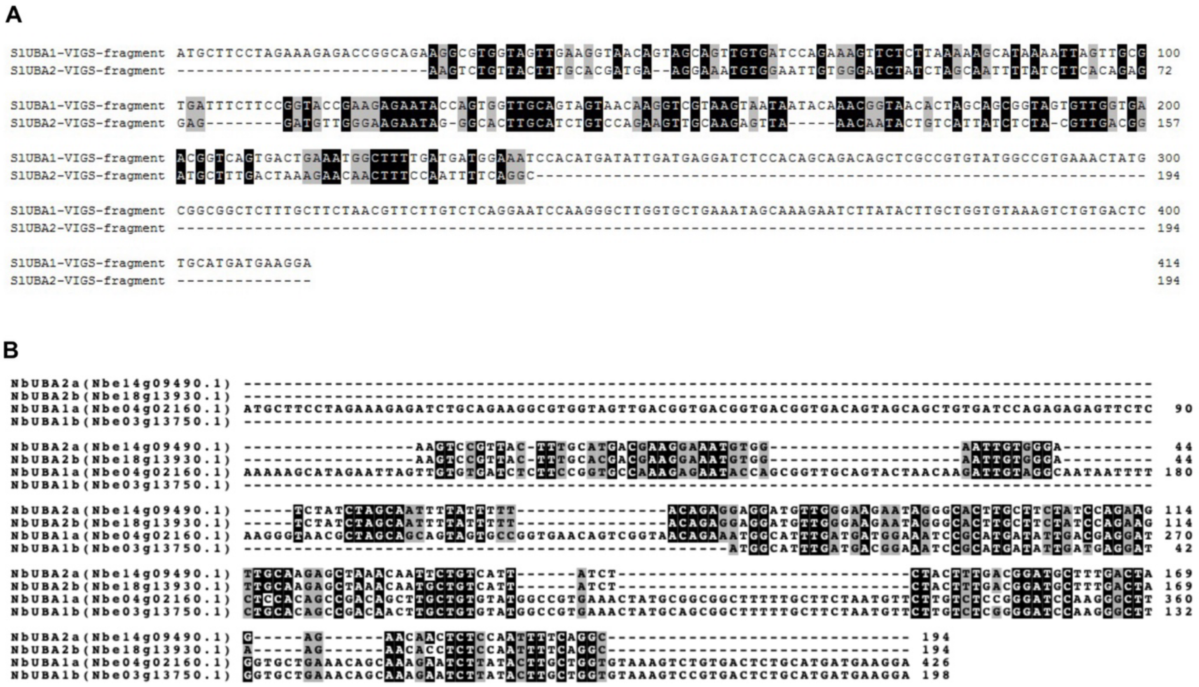

Supplementary Figure 6. The DNA fragments used for VIGS of *SlUBA1/NbUBA1a/1b* and *SlUBA2/NbUBA2a/2b*, respectively share minimal homology. (**A**) DNA sequence alignment of tomato *SlUBA1* and *SlUBA2* gene fragments used for silencing *SlUBA1 and SlUBA2*, respectively. (**B**) DNA sequence alignment of *N. benthamiana NbUBA1a/1b* and *NbUBA2a/2b* gene fragments that are targeted for virus-induced gene silencing. Identical DNA sequences of the three genes examined are highlighted in black.
