# Supplementary material for: The Two Tomato Ubiquitin E1 Enzymes Play Unequal Roles in Host Immunity
Source: Mol Plant Pathol. 2025 Sep 29;26(10):e70160. doi: 10.1111/mpp.70160 (PMC12477439; doi:10.1111/mpp.70160)
Supplement: Supplementary file 9 — Figure S7: The ubiquitin E1 genes SlUBA1 and SlUBA2, NbUBA1a/1b and NbUBA2a/2b were specifically and efficiently silenced in tomato and N. benthamiana plants. [file MPP-26-e70160-s017.pdf]

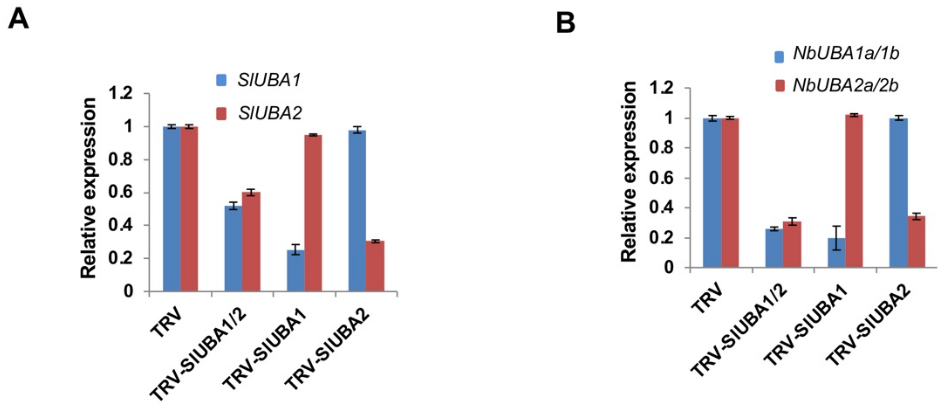

Supplementary Figure 7. The ubiquitin E1 genes *UBA1* and *UBA2* were specifically and efficiently silenced in tomato and tobacco plants.

The efficiency and specificity of silencing the E1 genes *SlUBA1* and *SlUBA2* in tomato **(A)** as well as *NbUBA1a/1b* and *NbUBA2a/2b* in *N. benthamiana* **(B)** were determined. The level of *SlUBA1* and *SlUBA2* as well as *NbUBA1a/1b* and *NbUBA2a/2b* transcripts in TRV-*SlUBA1*-, TRV-*SlUBA2*-, TRV-*SlBA1/2*- and empty vector (TRV)-infected tomato **(A)** and *N. benthamiana* **(B)** plants was examined by qRT-PCR. *EF1α* was used as an internal reference for the determination of the amount of cDNA template to be used.
