# Supplementary material for: The Two Tomato Ubiquitin E1 Enzymes Play Unequal Roles in Host Immunity
Source: Mol Plant Pathol. 2025 Sep 29;26(10):e70160. doi: 10.1111/mpp.70160 (PMC12477439; doi:10.1111/mpp.70160)
Supplement: Supplementary file 10 — Figure S8: Effects of E1 gene silencing on leaf development in tomato and Nicotiana benthamiana. [file MPP-26-e70160-s019.pdf]

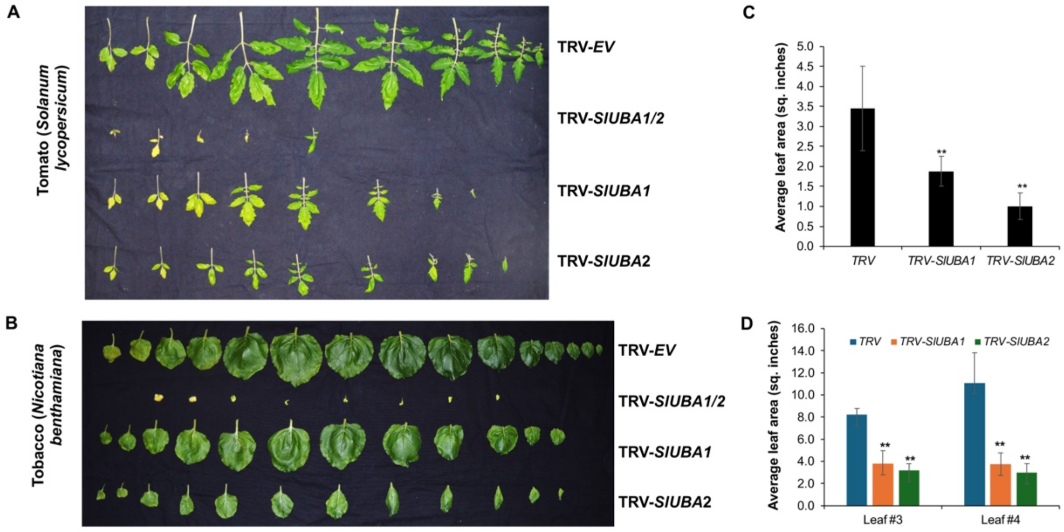

Supplementary Figure 8. Effects of E1 gene silencing on leaf development in tomato and *Nicotiana benthamiana*.

(**A**, **B**) Representative compound leaves of tomato (upper panel, **A**) and leaves of N. benthamiana (lower panel, **B**) from virus-induced gene silencing (VIGS)-treated plants, photographed ~6 weeks post-infiltration. Plants silenced for *SlUBA1*, *SlUBA2*, *NbUBA1a/1b*, or *NbUBA2a/2b* exhibited slower growth compared to those infiltrated with TRV empty vector (control). In *N. benthamiana*, control plants developed ~15 leaves, while *NbUBA1a/1b*- or *NbUBA2a/2b*-silenced plants developed ~13 leaves by 6 weeks post-infiltration. Plants with all E1 genes silenced showed severe developmental defects. (**C**) Average area of fully expanded tomato leaves (from the second to fourth compound leaves, starting at the top of the plant) from *SlUBA1*- or *SlUBA2*-knockdown and TRV empty vector (control) plants. (**D**) Average area of expanded N. benthamiana leaves (third and fourth leaves) from *NbUBA1a/1b*- or *NbUBA2a/2b*-knockdown and TRV empty vector (control) plants. Leaf areas were measured ~4 weeks post-VIGS infiltration using ImageJ, with at least eight tomato leaves and four *N. benthamiana* leaves (the third and fourth, respectively) from a minimum of four plants per treatment analyzed. Statistical analysis was performed using the Tukey-Kramer HSD test. $*P < 0.05$, $**P < 0.01$.
