# Supplementary material for: The Two Tomato Ubiquitin E1 Enzymes Play Unequal Roles in Host Immunity
Source: Mol Plant Pathol. 2025 Sep 29;26(10):e70160. doi: 10.1111/mpp.70160 (PMC12477439; doi:10.1111/mpp.70160)
Supplement: Supplementary file 11 — Figure S9: Effects of E1 gene silencing on leaf development in tomato and Nicotiana benthamiana. [file MPP-26-e70160-s002.pdf]

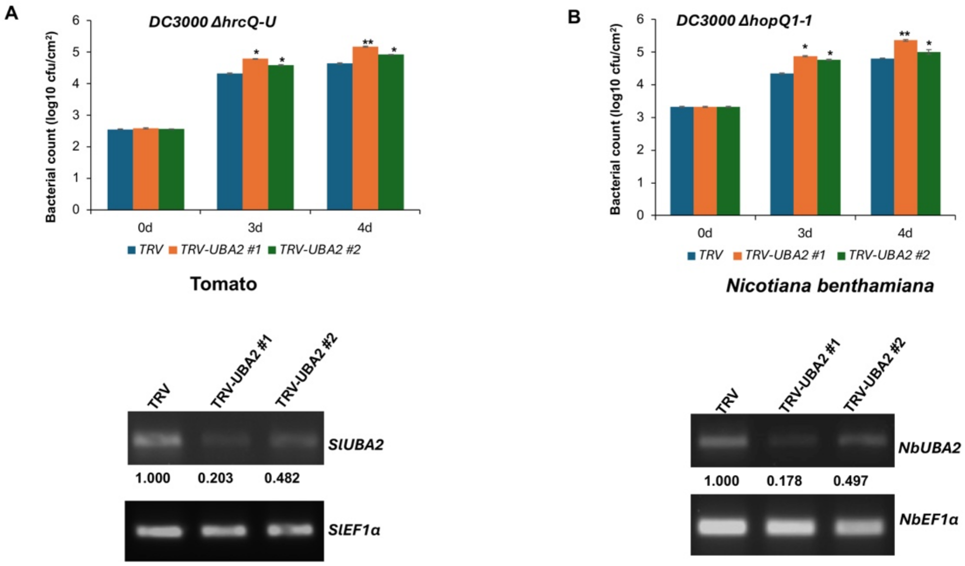

Supplementary Figure 9. ***UBA2*** **expression levels correlate with plant immunity in tomato and** ***N. benthamiana*****.**

(**A**) Tomato and (**B**) *N. benthamiana* plants with varying levels of *SlUBA2* or *NbUBA2a/b* silencing via virus-induced gene silencing (VIGS) were categorized into two groups: Group 1 (TRV-*UBA2* #1) with stronger silencing and Group 2 (TRV-*UBA2* #2) with weaker silencing. Bacterial growth of *Pseudomonas syringae pv. tomato (Pst)* DC3000*ΔhrcQ-U* or DC3000*ΔhopQ1-1* was assessed in plants ~4 weeks post-VIGS infiltration. Statistical significance was determined using one-way ANOVA (*$P < 0.05$, **$P < 0.01$). Lower panels show semi-quantitative PCR analysis of *SlUBA2* (**A**) and *NbUBA2a/b* (**B**) expression in silenced and control plants, with relative expression levels indicated below the gel bands.
