# Supplementary material for: The Two Tomato Ubiquitin E1 Enzymes Play Unequal Roles in Host Immunity
Source: Mol Plant Pathol. 2025 Sep 29;26(10):e70160. doi: 10.1111/mpp.70160 (PMC12477439; doi:10.1111/mpp.70160)
Supplement: Supplementary file 12 — Figure S10: Systematic analysis of the efficiencies in charging ubiquitin E2 enzymes by SlUBA1 and SlUBA2. [file MPP-26-e70160-s007.pdf]

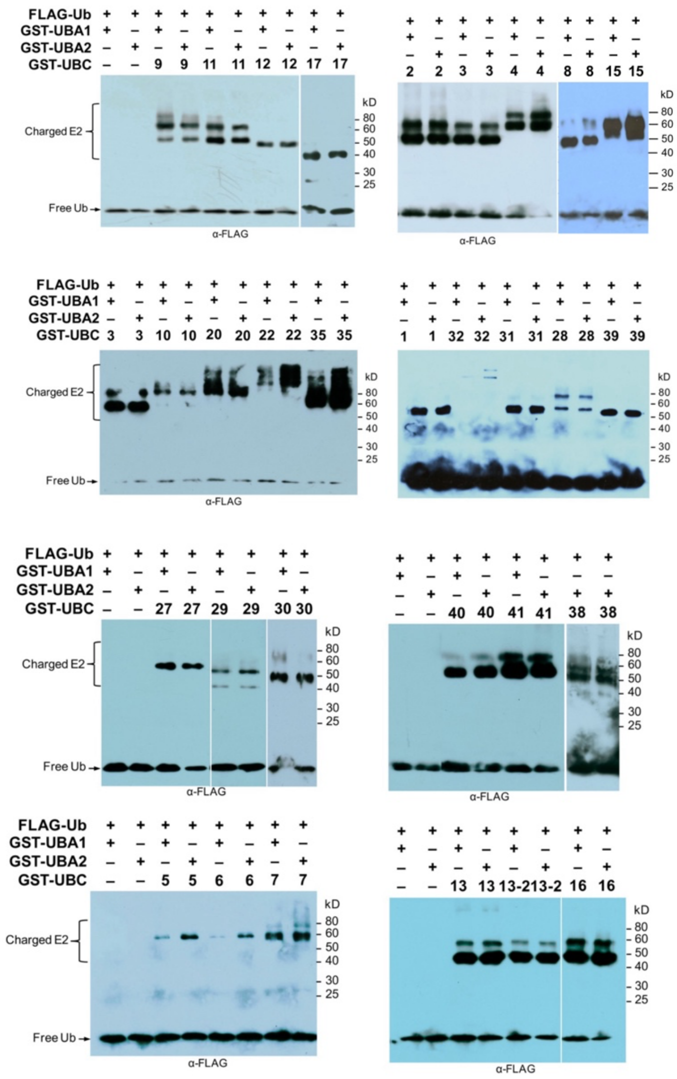

Supplementary Figure 10. Systematic analysis of the efficiencies in charging tomato ubiquitin E2 enzymes by tomato E1s SlUBA1 and SlUBA2. The thioester assay was used to examine efficiencies in E2 charging by two tomato E1s. Purified recombinant protein of tomato ubiquitin E2s and FLAG-tagged free ubiquitin (FLAG-Ub) were incubated with GST-tagged SlUBA1 and SlUBA2, respectively, at 30°C for 15 min and the reactions were then terminated by adding SDS sample loading buffer with 4M Urea. The formation of ubiquitin adducts to tomato E2s is denoted as charged E2. The numbers on the right denote the molecular mass of marker proteins in kilodaltons.
