# Supplementary material for: The Two Tomato Ubiquitin E1 Enzymes Play Unequal Roles in Host Immunity
Source: Mol Plant Pathol. 2025 Sep 29;26(10):e70160. doi: 10.1111/mpp.70160 (PMC12477439; doi:10.1111/mpp.70160)
Supplement: Supplementary file 13 — Figure S11: Comparison of the efficiencies in E2 charging by Human and tomato E1s. [file MPP-26-e70160-s015.pdf]

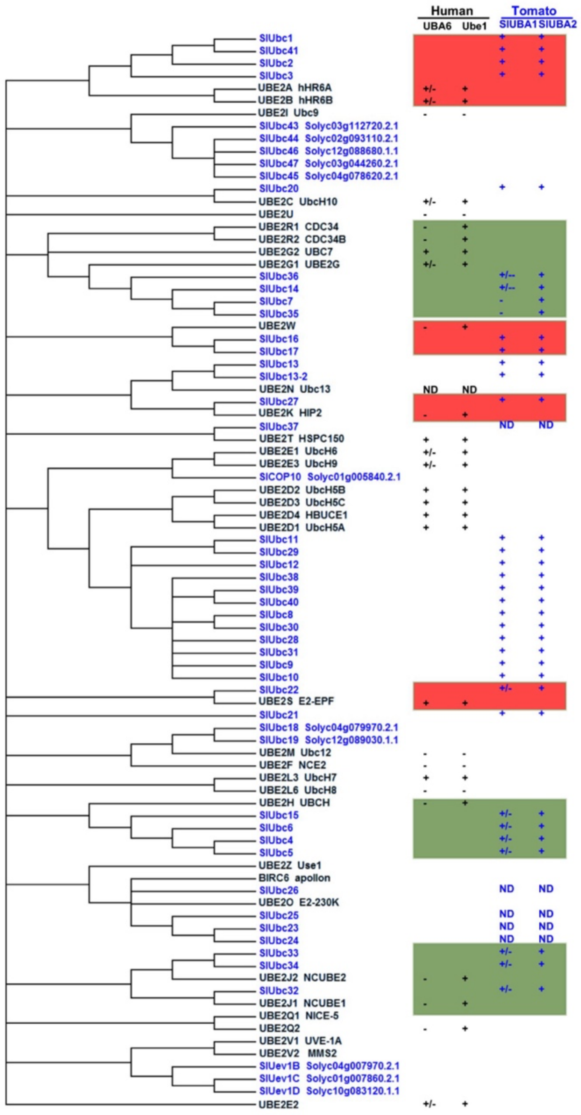

Supplementary Figure 11. Comparison of the efficiencies in E2 charging by Human and tomato E1s. The unrooted phylogenetic tree on the left was constructed based on sequences of human (names in black color) and tomato (names in blue color) ubiquitin E2 proteins. The phylogenetic tree is drawn to scale, with branch lengths in the same units as those of the evolutionary distances used to infer the phylogenetic tree. The middle columns in black font show the efficiencies of charging human E2s by human E1 UBA6 and UBE1(Jin *et al.*, 2007). The columns on the right in blue font show the efficiencies of charging E2s by tomato E1 SlUBA1 and SlUBA2. +, charging; -, no charging, +/- weak charging, +/-- very weak charging. Phylogenetically conserved human and tomato E2s that are both differently charged by corresponding dual ubiquitin E1 activation systems are highlighted in green; E2s that are phylogenetically conserved but only differently charged by either the human or the tomato dual ubiquitin E1 activation systems are highlighted in red.

Reference

Jin, J., Li, X., Gygi, S. P. and Harper, J. W. (2007) Dual E1 activation systems for ubiquitin differentially regulate E2 enzyme charging. *Nature,* **447,** 1135–1138.
