# Supplementary material for: The Two Tomato Ubiquitin E1 Enzymes Play Unequal Roles in Host Immunity
Source: Mol Plant Pathol. 2025 Sep 29;26(10):e70160. doi: 10.1111/mpp.70160 (PMC12477439; doi:10.1111/mpp.70160)
Supplement: Supplementary file 14 — Figure S12: The UFD is an important but not sole factor that governs the specificities of E2 charging by tomato ubiquitin E1s. [file MPP-26-e70160-s003.pdf]

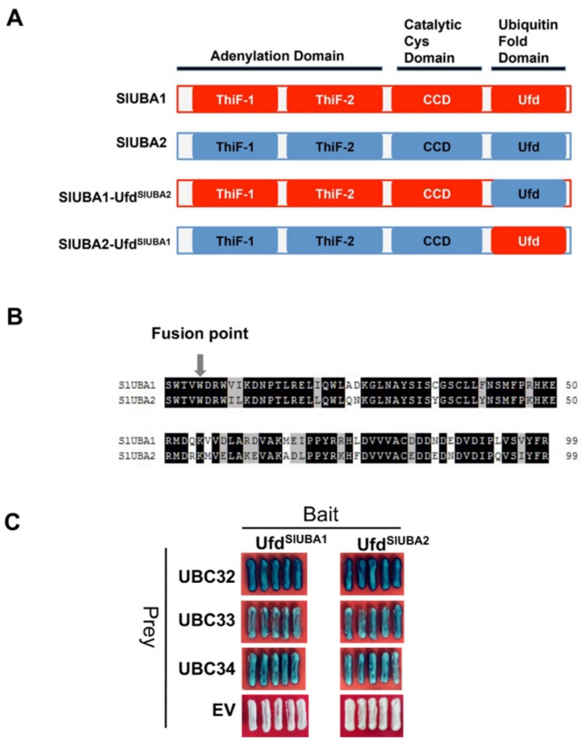


**Supplementary Figure 12. The UFD is an important but not sole factor that contribute to the efficiencies of E2 charging by tomato ubiquitin E1s.**

**(A)** Schematic representation of domain organization for the SlUBA1, SlUBA2 and the chimeric SlUBA1-Ufd$^{SlUBA2}$ and SlUBA2-Ufd$^{SlUBA1}$ protein. **(B)** Amino acid sequences of the UFD from SlUBA1 and SlUBA2. Identical and highly conserved amino acid residues in SlUBA1 and SlUBA2 are highlighted in black and grey, respectively. The arrow marks the position where the exchanged UFD domains are fused. **(C)** The UFD domain from SlUBA1 (Ufd$^{SlUBA1}$) and SlUBA2 (Ufd$^{SlUBA2}$) displayed comparable interaction intensity with members of the tomato group IV E2s in yeast two-hybrid assay. Photographs were taken 24 h after the yeast cells expressed the indicated bait and prey were transferred to X-gal-containing media.
