# Supplementary material for: The Two Tomato Ubiquitin E1 Enzymes Play Unequal Roles in Host Immunity
Source: Mol Plant Pathol. 2025 Sep 29;26(10):e70160. doi: 10.1111/mpp.70160 (PMC12477439; doi:10.1111/mpp.70160)
Supplement: Supplementary file 15 — Figure S13: Protein sequence alignment of C‐terminal region of ubiquitin E1s from Arabidopsis, tomato, N. benthamiana and rice. [file MPP-26-e70160-s008.pdf]

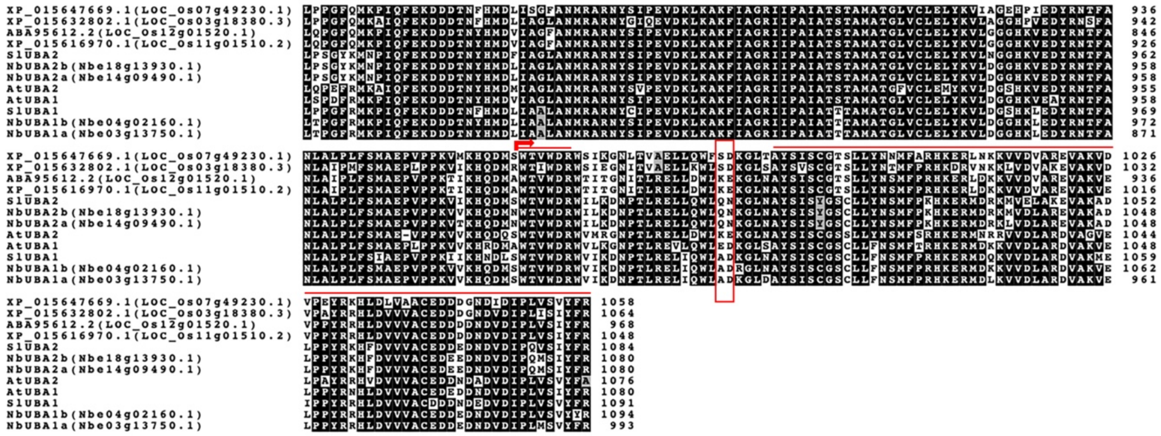

Supplementary Figure 13. Alignment of C-terminal protein sequence of ubiquitin E1s from Arabidopsis, tomato *N. benthamiana*, and rice.

C-terminal Protein sequence of ubiquitin E1s from Arabidopsis, tomato *N. benthamiana*, and rice harboring the UFD were input in FASTA format and aligned using the Clustal Omega algorithm (Sievers et al., 2011). The arrow denotes the start amino acid residue of the UFD. The red line above the sequence marks the regions that are putatively involved in forming the interface for interaction with E2. The red box mark residues of the plant E1s that show high variation at the positions.
