# Supplementary material for: The Two Tomato Ubiquitin E1 Enzymes Play Unequal Roles in Host Immunity
Source: Mol Plant Pathol. 2025 Sep 29;26(10):e70160. doi: 10.1111/mpp.70160 (PMC12477439; doi:10.1111/mpp.70160)
Supplement: Supplementary file 16 — Figure S14: Predicted three‐dimensional structure of tomato E1s SlUBA1 and SlUBA2 by AlphaFold3. [file MPP-26-e70160-s016.pdf]

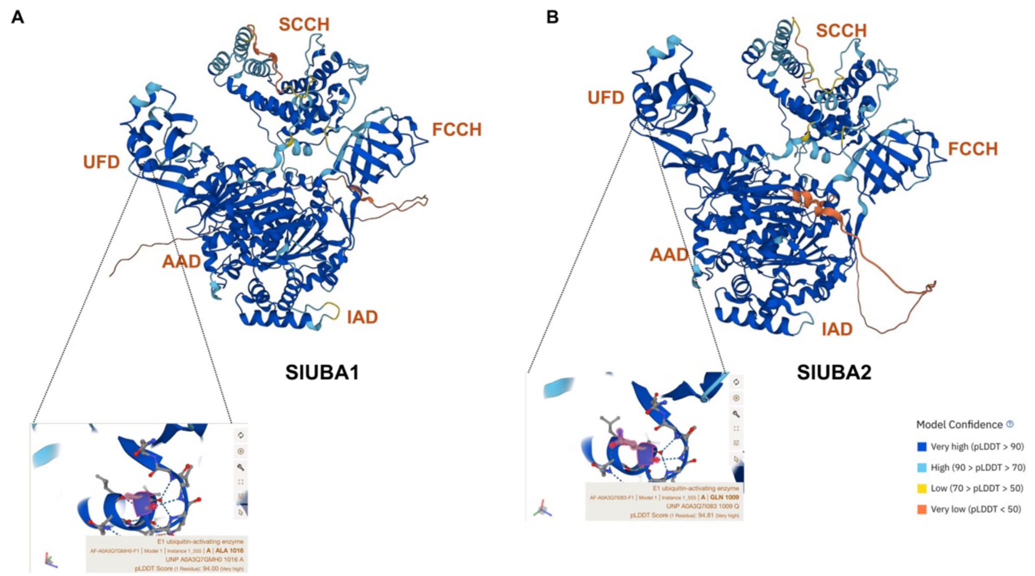

Supplementary Figure 14. Predicted three-dimensional structure of tomato E1s SlUBA1 and SlUBA2 by AlphaFold3.

The predicted 3-dimensional structure of SlUBA1 (Uniprot ID: A0A3Q7GMH0) and SlUBA2 (Uniprot ID: A0A3Q7I083) was retrieved from the online AlphaFold protein structure database (https://alphafold.ebi.ac.uk). AlphaFold produces a per-residue model confidence score (pLDDT) between 0 and 100. Some regions below 50 pLDDT may be unstructured in isolation. pLDDT corresponds to the model's prediction of its score on the local Distance Difference Test (lDDT-Cα). It is a measure of local accuracy - for interpreting larger scale features like relative domain positions. Model confidence bands are used to color-code the residues in the 3D viewer. The Zoom in windows show the Gln and Ala residue (red boxed) at the Gln^{1009} position of SlUBA2. UFD (ubiquitin-fold domain), AAD (active adenylation domain), IAD (inactive adenylation domain), FCCH (first catalytic cysteine half-domain), SCCH (second catalytic cysteine half-domain).
