# Supplementary material for: The Two Tomato Ubiquitin E1 Enzymes Play Unequal Roles in Host Immunity
Source: Mol Plant Pathol. 2025 Sep 29;26(10):e70160. doi: 10.1111/mpp.70160 (PMC12477439; doi:10.1111/mpp.70160)
Supplement: Supplementary file 17 — Figure S15: Protein sequence alignment of tomato and Arabidopsis UBC3, UBC12 and group V E2s, and predicted three‐dimensional structure of tomato UBC7 and UBC12 by AlphaFold3. [file MPP-26-e70160-s018.pdf]

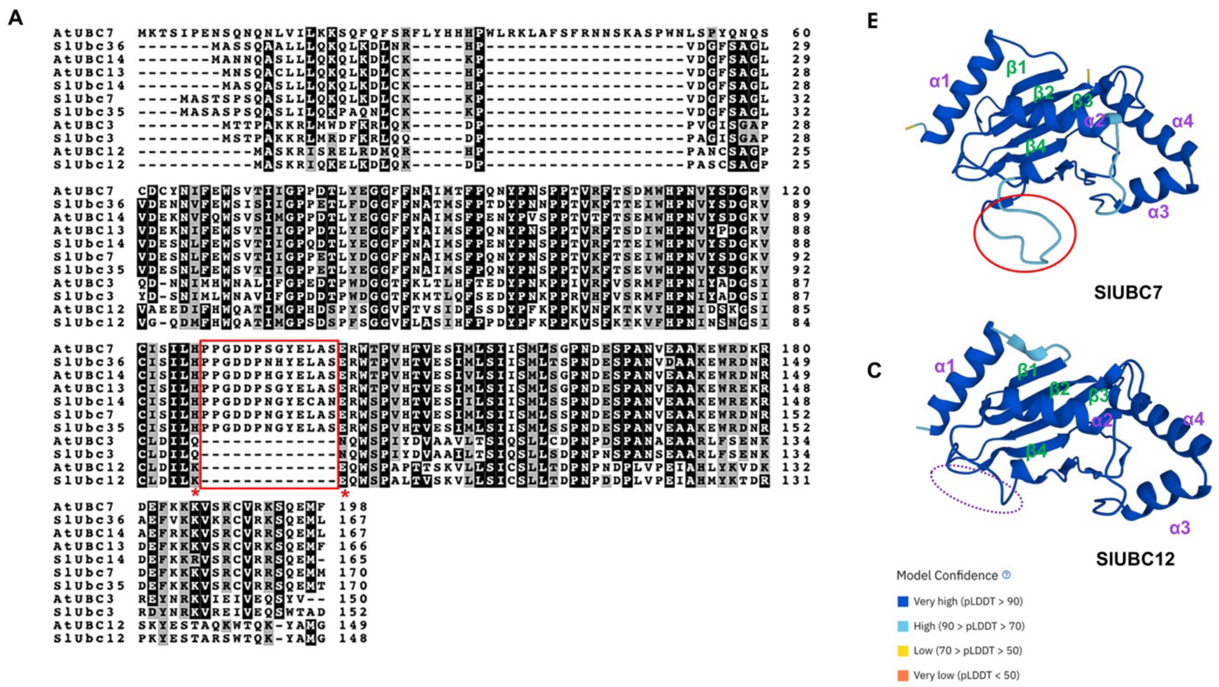

Supplementary Figure 15. Protein sequence alignment of tomato and Arabidopsis group V E2s, UBC3 and UBC12 and predicted three-dimensional structure of tomato UBC7 and UBC12 by AlphaFold3. (**A**)The protein sequence of indicated E2 enzymes were input in FASTA format and aligned using the T-Coffee algorithm. Black shaded residues denote >70% conserved among the E1s. Gray shaded mark residues from similar group (e.g. both amino acid residues have positively charged side chain). None shaded denote residues different from the residue that is black shaded or with high variation (<70% conservation). Red box denotes the 13 high conserved amino acid residues (13aa) insertion among group V E2s. Asterisks mark the two residues of SlUBC12 into which the 13aa are inserted for building the SlUBC12^{+13aa} mutant. (**B**) and (**C**) Predicted 3-dimensional structure of SlUBC7 (Uniprot ID: K4C2D3) and SlUBC12 (Uniprot ID: K4CHT4) by AlphaFold3. AlphaFold produces a per-residue model confidence score (pLDDT) between 0 and 100. Some regions below 50 pLDDT may be unstructured in isolation. pLDDT corresponds to the model's prediction of its score on the local Distance Difference Test (lDDT-Cα). It is a measure of local accuracy - for interpreting larger scale features like relative domain positions. Model confidence bands are used to color-code the residues in the 3D viewer.
