# Supplementary material for: The Two Tomato Ubiquitin E1 Enzymes Play Unequal Roles in Host Immunity
Source: Mol Plant Pathol. 2025 Sep 29;26(10):e70160. doi: 10.1111/mpp.70160 (PMC12477439; doi:10.1111/mpp.70160)
Supplement: Supplementary file 18 — Figure S16: Group IV E2s exhibit stronger interaction with SlUBA2 than with SlUBA1 in yeast two‐hybrid assay. [file MPP-26-e70160-s020.pdf]

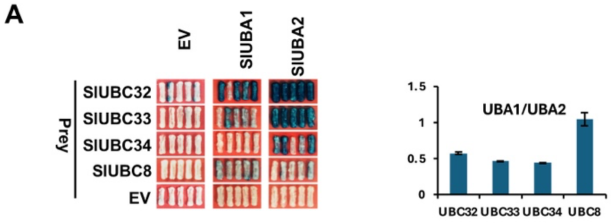


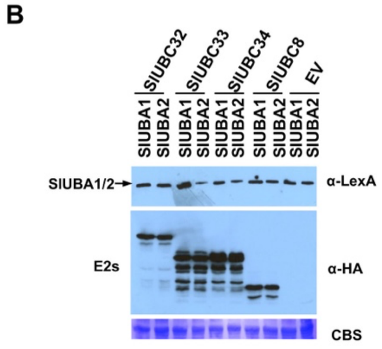


**Supplementary Figure 16. Group IV E2s interact stronger with SlUBA2 than with SlUBA1 in yeast two-hybrid assay.**

(**A**) The interaction of SlUBA1 and SlUBA2 with group IV E2s was detected by yeast two-hybrid. SlUBC8 and the empty prey and bait vectors (EV) were used as control. Interaction was demonstrated by activation of the lacZ reporter gene (blue patches). Photographs were taken 24 h after streaking the yeast cells onto plates containing X-Gal. The right panel shows ratio of blue color intensity in yeast cell patches of UBA1-E2 compared to that of the UBA2-E2 as shown in the left panel. (**B**) Comparable levels of SlUBA1 and SlUBA2 protein and similar level of the E2 protein were expressed for each pair of SlUBA1-E2 and SlUBA2-E2 in the yeast cells of the Y2H assay as shown in (**A**). CBB, Coomassie blue staining as an indicator of equal sample loading.
