# Supplementary material for: The Two Tomato Ubiquitin E1 Enzymes Play Unequal Roles in Host Immunity
Source: Mol Plant Pathol. 2025 Sep 29;26(10):e70160. doi: 10.1111/mpp.70160 (PMC12477439; doi:10.1111/mpp.70160)
Supplement: Supplementary file 19 — Figure S17: Expression levels of IAA17 and JAZ1 homologues in tomato and N. benthamiana plants with silenced ubiquitin E1 genes. [file MPP-26-e70160-s021.pdf]

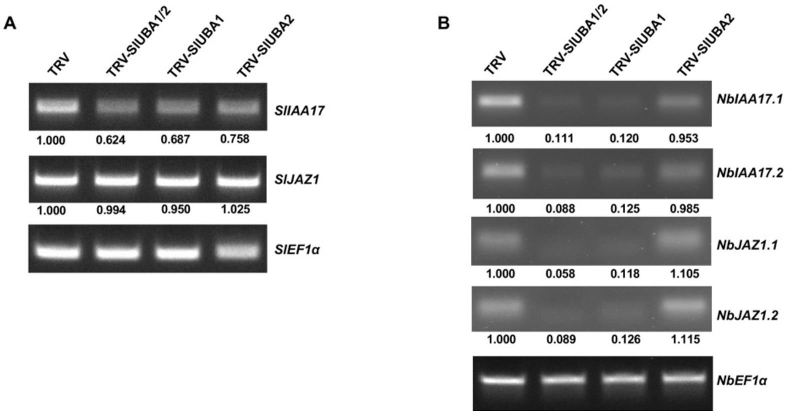

Supplementary Figure 17. Expression levels of *IAA17* and *JAZ1* homologs in tomato and *N. benthamiana* plants with silenced ubiquitin E1 genes. Expression of *SlIAA17* (Solyc06g053830), *SlJAZ1* (Solyc07g042170), *NbIAA17.1* (Nbe01g31880), *NbIAA17.2* (Nbe02g04210), *NbJAZ1.1* (Nbe14g17870), and *NbJAZ1.2* (Nbe16g02010) was assessed in tomato and *N. benthamiana* plants infiltrated with TRV-*SlUBA1*, TRV-*SlUBA2*, TRV-*SlUBA1/2*, or TRV control using semi-quantitative PCR. Relative expression levels, normalized to a housekeeping gene EF1α, are indicated below the gel bands. This experiment was performed twice with consistent results.
