# Supplementary material for: The Two Tomato Ubiquitin E1 Enzymes Play Unequal Roles in Host Immunity
Source: Mol Plant Pathol. 2025 Sep 29;26(10):e70160. doi: 10.1111/mpp.70160 (PMC12477439; doi:10.1111/mpp.70160)
Supplement: Supplementary file 20 — Table S1: Ubiquitin‐activating enzymes (E1) encoded by crop and model plant genomes. [file MPP-26-e70160-s006.docx]

**Supplementary Table 1 Ubiquitin-activating enzymes encoded by crop and model plant genomes**

| **Common Name** | **Scientific Name, Ploidy level, n*** | **Family** | **Number of Ub E1** | **GenBank Accession Number** | **References**  **(genome sequencing)** | **Note** |
| --- | --- | --- | --- | --- | --- | --- |
| African oil palm | *Elaeis guineensis*, 2X, n=16 | *Arecaceae* | 2 | XP_010922601, XP_010937921 | (*1*) |  |
| Arabidopsis (thale cress) | *Arabidopsis thaliana*, 2X, n=5 | *Brassicaceae* | 2 | AAB39246, AAB37569 | (*2, 3*) | Ecotype: Columbia |
| Banana | *Musa acuminata*, 2X, n=11 | *Musaceae* | 2 | >XP_009420363.2, >XP_009412523.2 | (*4*) | There is alternative splicing for each E1 gene |
| Barrel Medic | *Medicago truncatula*, 2X, n=8 | *Fabaceae* | 2 | XP_024640200.1, XP_024640279.1 | (*5*) | Model legume |
| Bread wheat | *Triticum aestivum*, 6X, n=21 | *Poaceae* | 9 | NP_001392904, XP_044375515, XP_044374377, XP_044364016, XP_044363192, XP_044330208, XP_044321962, XP_044396439, XP_044455149 | (*6*) | There are isoforms for some E1s. |
| Cocoa tree | *Theobroma cacao*, 2X, n=10 | *Malvaceae* | 2 | XP_017982608.1, XP_017983162.1 | (*7*) |  |
| Cucumber | *Cucumis sativus*, 2X, n=7 | *Cucurbitaceae* | 1 | XP_011657116.1 | (*8*) | *Cucumis sativus* var. *sativus* L. |
| Foxtail millet | *Setaria italica*, 2X, n=9 | *Poaceae* | 4 | XP_004977630.1, XP_004958791.1, XP_012698263.1, XP_004978585.1 | (*9*) | Model of C4 metabolism |
| Grape | *Vitis vinifera*, 2X, n=19 | *Vitaceae* | 2 | XP_002275617.3, XP_010653400.1 | (*10*) |  |
| Lettuce | *Lactuca sativa*, 2X, n=9 | *Asteraceae* | 2 | XP_023728772.1, XP_023760160.1 | (*11*) |  |
| Maize | *Zea mays*, 2X, n=10 | *Poaceae* | 5 | XP_008654582.1, XP_008653531.1, XP_008668365.1, NP_001348488.1, NP_001304750.1 | (*12, 13*) | Genome of cultivar B73 |
| Papaya | *Carica papaya*, 2X, n=9 | *Caricaceae* | 2 | XP_021909681.1, XP_021909824.1 | (*14*) |  |
| Peach | *Prunus persica*, 2X, n=8 | *Rosaceae* | 2 | XP_007208126.2, XP_007208384.2, | (*15*) | XP_007208126.2 has two isoforms |
| Pepper | *Capsicum annuum*, 2X, n=12 | *Solanaceae* | 2 | XP_016542098.2, XP_016576007.1 | (*16*) | Cultivar Zunla-1. XP_016576007.1 has an isoform (XP_047258385.1) |
| Pineapple | *Ananas comosus (L.) Merr.*, 2X, 2n=25 | *Bromeliaceae* | 2 | XP_020101243.1, OAY74399 | (*17*) |  |
| Pomegranate | *Punica granatum*, 2X, n=8 | *Lythraceae* | 2 | XP_031388388.1, XP_031401802.1 | (*18*) |  |
| Potato | *Solanum tuberosum*, 4X, n=24 | *Solanaceae* | 4 | XP_006344068.1, XP_006359551.1  KAH0667809.1, KAH0750677.1 | (*19*) | XP_006359551.1, KAH0641011.1, KAH0637923.1, and KAH0632828.1 are likely encode by the same gene. |
| Rapeseed | *Brassica napus*, 4X (allotetraploid), n=19 | *Brassicaceae* | 8 | XP_048636812.1, XP_048608361.1, XP_048630387.1, XP_048610284.1, XP_013731175.2, XP_013715587.2, XP_048633816.1, XP_048630287.1 | (*20*) | European winter oilseed cultivar ‘Darmor-bzh' |
| Rice | *Oryza sativa*, 2X, n=12 | *Poaceae* | 4 | ABA95612.2 (LOC_Os12g01520.1), XP_015632802.1 (LOC_Os03g18380.3),  XP_015616970.1 (LOC_Os11g01510.2), XP_015647669.1 (LOC_Os07g49230.1) | (*21, 22*) | https://rice.uga.edu |
| Rose gum | *Eucalyptus grandis*, 2X, n=11 | *Myrtaceae* | 2 | XP_010043403.1, XP_010033000.2 | (*23*) | Fibre and timber crop |
| Sorghum | *Sorghum bicolor*, 2X, n=10 | *Poaceae* | 4 | XP_002450102.1, XP_002461268.1, XP_021306927.1, XP_002442655.1 | (*24*) |  |
| Soybean | *Glycine max*, 2X, n=20 | *Fabaceae* | 4 | XP_006602078.1, XP_003544897.2, XP_003518319.2, XP_003537305.1, | (*25, 26*) | Variety Williams 82 |
| sugar beet | *Beta vulgaris*, 2X, n=9 | *Chenopodiaceae* | 2 | XP_010685926.1, XP_010676802.1 | (*27*) |  |
| Sunflower | *Helianthus annuus*, 2X, n=17 | *Asteraceae* | 4 | XP_021977875.1, XP_022034390.1, XP_022034191.1，XP_021970716.1 | (*28*) |  |
| Sweet Orange | *Citrus sinensis*, 2X, n=9 | *Rutaceae* | 2 | XP_024957890.1, XP_006481094.1 | (*29*) |  |
| Tobacco | *Nicotiana benthamiana*, 4X (allotetraploid), n=19 | *Solanaceae* | 4 | Nbe04g02160.1, Nbe03g13750.1, Nbe14g09490.1, Nbe18g13930.1 | (*30*)** |  |
| Tobacco | *Nicotiana tabacum*, 4X, n=24 | *Solanaceae* | 4 | Nta04g02230, Nta03g01970, Nta18g08720, Nta17g06720 | (*30*)** |  |
| Tomato | *Solanum lycopersicum*, 2X, n=12 | *Solanaceae* | 2 | XP_004240416.1, XP_004246264.1 | (*31*) | Cultivar Heinz 1706 |

*: “n” refers to the number of chromosomes for haploid.

**: The recently published *N. benthamiana* and *N. tabacum* genome sequences apparently are not deposited to the NCBI database. But they can be found at <http://lifenglab.hzau.edu.cn/Nicomics/index.php>.

**References**

1. R. Singh *et al.*, Oil palm genome sequence reveals divergence of interfertile species in Old and New worlds. *Nature* **500**, 335-339 (2013).

2. I. The Arabidopsis Genome, Analysis of the genome sequence of the flowering plant Arabidopsis thaliana. *Nature* **408**, 796-815 (2000).

3. P. M. Hatfield, M. M. Gosink, T. B. Carpenter, R. D. Vierstra, The ubiquitin-activating enzyme (E1) gene family in Arabidopsis thaliana. *Plant J.* **11**, 213-226 (1997).

4. A. D’Hont *et al.*, The banana (Musa acuminata) genome and the evolution of monocotyledonous plants. *Nature* **488**, 213-217 (2012).

5. N. D. Young *et al.*, The Medicago genome provides insight into the evolution of rhizobial symbioses. *Nature* **480**, 520-524 (2011).

6. P. M. Hatfield, R. D. Vierstra, Multiple forms of ubiquitin-activating enzyme E1 from wheat. Identification of an essential cysteine by in vitro mutagenesis. *J. Biol. Chem.* **267**, 14799-14803 (1992).

7. X. Argout *et al.*, The genome of Theobroma cacao. *Nature Genetics* **43**, 101-108 (2011).

8. S. Huang *et al.*, The genome of the cucumber, Cucumis sativus L. *Nature Genetics* **41**, 1275-1281 (2009).

9. J. L. Bennetzen *et al.*, Reference genome sequence of the model plant Setaria. *Nature Biotechnology* **30**, 555-561 (2012).

10. O. Jaillon *et al.*, The grapevine genome sequence suggests ancestral hexaploidization in major angiosperm phyla. *Nature* **449**, 463-467 (2007).

11. S. Reyes-Chin-Wo *et al.*, Genome assembly with in vitro proximity ligation data and whole-genome triplication in lettuce. *Nature Communications* **8**, 14953 (2017).

12. Y. Jiao *et al.*, Improved maize reference genome with single-molecule technologies. *Nature* **546**, 524-527 (2017).

13. P. S. Schnable *et al.*, The B73 Maize Genome: Complexity, Diversity, and Dynamics. *Science* **326**, 1112 (2009).

14. R. Ming *et al.*, The draft genome of the transgenic tropical fruit tree papaya (Carica papaya Linnaeus). *Nature* **452**, 991-996 (2008).

15. I. Verde *et al.*, The high-quality draft genome of peach (Prunus persica) identifies unique patterns of genetic diversity, domestication and genome evolution. *Nature Genetics* **45**, 487-494 (2013).

16. C. Qin *et al.*, Whole-genome sequencing of cultivated and wild peppers provides insights into &lt;em&gt;Capsicum&lt;/em&gt; domestication and specialization. *Proceedings of the National Academy of Sciences* **111**, 5135 (2014).

17. R. Ming *et al.*, The pineapple genome and the evolution of CAM photosynthesis. *Nature Genetics* **47**, 1435-1442 (2015).

18. Z. Yuan *et al.*, The pomegranate (*Punica granatum L*.) genome provides insights into fruit quality and ovule developmental biology. *Plant Biotechnol J* **16**, 1363-1374 (2018).

19. X. Xu *et al.*, Genome sequence and analysis of the tuber crop potato. *Nature* **475**, 189-195 (2011).

20. B. Chalhoub *et al.*, Early allopolyploid evolution in the post-Neolithic &lt;em&gt;Brassica napus&lt;/em&gt; oilseed genome. *Science* **345**, 950 (2014).

21. S. A. Goff *et al.*, A Draft Sequence of the Rice Genome (Oryza sativa L. ssp. japonica). *Science* **296**, 92 (2002).

22. John P. Hamilton, C. Li, C. R. Buell, The rice genome annotation project: an updated database for mining the rice genome. *Nucleic Acids Res.* **53**, D1614-D1622 (2024).

23. A. A. Myburg *et al.*, The genome of Eucalyptus grandis. *Nature* **510**, 356-362 (2014).

24. A. H. Paterson *et al.*, The Sorghum bicolor genome and the diversification of grasses. *Nature* **457**, 551-556 (2009).

25. J. Schmutz *et al.*, Genome sequence of the palaeopolyploid soybean. *Nature* **463**, 178-183 (2010).

26. C. Zhang *et al.*, Genome-wide analysis of genes encoding core components of the ubiquitin system in soybean (Glycine max) reveals a potential role for ubiquitination in host immunity against soybean cyst nematode. *BMC Plant Biol.* **18**, (2018).

27. J. C. Dohm *et al.*, The genome of the recently domesticated crop plant sugar beet (Beta vulgaris). *Nature* **505**, 546-549 (2014).

28. H. Badouin *et al.*, The sunflower genome provides insights into oil metabolism, flowering and Asterid evolution. *Nature* **546**, 148-152 (2017).

29. Q. Xu *et al.*, The draft genome of sweet orange (Citrus sinensis). *Nature Genetics* **45**, 59-66 (2013).

30. J. Wang *et al.*, High-quality assembled and annotated genomes of Nicotiana tabacum and Nicotiana benthamiana reveal chromosome evolution and changes in defense arsenals. *Mol Plant* **17**, 423-437 (2024).

31. S. Sato *et al.*, The tomato genome sequence provides insights into fleshy fruit evolution. *Nature* **485**, 635-641 (2012).
