# Supplementary material for: The Two Tomato Ubiquitin E1 Enzymes Play Unequal Roles in Host Immunity
Source: Mol Plant Pathol. 2025 Sep 29;26(10):e70160. doi: 10.1111/mpp.70160 (PMC12477439; doi:10.1111/mpp.70160)
Supplement: Supplementary file 21 — Table S2: SlUBA1 and SlUBA2 show differential efficiencies to four groups of E2s. [file MPP-26-e70160-s004.docx]

**Supplementary Table 2 SlUBA1 and SlUBA2 show differential efficiencies in charging four groups of tomato E2s**

| Chromosome locus | E2 Protein | Sub-Group | Efficiencies in E2 charging | |
| --- | --- | --- | --- | --- |
|  |  |  | **SlUBA1** | **SlUBA2** |
| **Solyc06g072570.2.1** | **SlUbc27** | I | + | + |
| **Solyc06g070980.2.1** | **SlUbc1** | II | + | + |
| **Solyc03g113100.2.1** | **SlUbc2** | II | + | + |
| **Solyc02g067420.2.1** | **SlUbc3** | II | + | + |
| **Solyc02g087750.2.1** | **SlUbc41** | II | + | + |
| **Solyc12g056100.1.1** | **SlUbc8** | III | + | + |
| **Solyc08g008220.2.1** | **SlUbc9** | III | + | + |
| **Solyc05g050230.2.1** | **SlUbc10** | III | + | + |
| **Solyc03g033410.2.1** | **SlUbc11** | III | + | + |
| **Solyc07g066080.2.1** | **SlUbc12** | III | + | + |
| **Solyc10g011740.2.1** | **SlUbc28** | III | + | + |
| **Solyc02g083570.2.1** | **SlUbc29** | III | + | + |
| **Solyc03g007470.2.1** | **SlUbc30** | III | + | + |
| **Solyc01g095490.2.1** | **SlUbc31** | III | + | + |
| **Solyc07g024070.1.1** | **SlUbc38** | III | + | + |
| **Solyc06g082600.2.1** | **SlUbc39** | III | + | + |
| **Solyc06g007510.2.1** | **SlUbc40** | III | + | + |
| **Solyc12g099310.1.1** | **SlUbc32** | IV | +/- | + |
| **Solyc03g123660.2.1** | **SlUbc33** | IV | +/- | + |
| **Solyc06g063100.2.1** | **SlUbc34** | IV | +/- | + |
| **Solyc05g054550.2.1** | **SlUbc7** | V | - | + |
| **Solyc04g011430.2.1** | **SlUbc14** | V | +/-- | + |
| **Solyc05g054540.2.1** | **SlUbc35** | V | - | + |
| **Solyc09g009720.1.1** | **SlUbc36** | V | +/-- | + |
| **Solyc10g012240.2.1** | **SlUbc4** | VI | +/- | + |
| **Solyc01g094810.2.1** | **SlUbc5** | VI | +/- | + |
| **Solyc08g081270.2.1** | **SlUbc6** | VI | +/- | + |
| **Solyc11g071870.1.1** | **SlUbc15** | VI | +/- | + |
| **Solyc11g071260.1.1** | **SlUbc21** | VII | ND | ND |
| **Solyc11g065190.1.1** | **SlUbc20** | VIII | + | + |
| **Solyc07g062570.2.1** | **SlUbc13** | IX | + | + |
| **Solyc10g007260.2.1** | **SlUbc13-2** | IX | + | + |
| **Solyc04g080810.2.1** | **SlUbc16** | X | + | + |
| **Solyc02g084760.2.1** | **SlUbc17** | X | + | + |
| **Solyc01g111680.2.1** | **SlUbc23** | XI | ND | ND |
| **Solyc02g078210.2.1** | **SlUbc24** | XI | ND | ND |
| **Solyc10g007000.2.1** | **SlUbc25** | XI | ND | ND |
| **Solyc01g079290.1.1** | **SlUbc26** | XI | ND | ND |
| **Solyc10g081160.1.1** | **SlUbc22** | XII | +/- | + |
| **Solyc07g024070.1.1** | **SlUbc37** | XIII | ND | ND |

+: similar efficiencies in being charged by SlUBA1 and SlUBA2; +/-: being charged at much lower efficiency; +/--: being charged at extremely lower efficiency; -: not being charged; ND: not tested
