# Supplementary material for: The Two Tomato Ubiquitin E1 Enzymes Play Unequal Roles in Host Immunity
Source: Mol Plant Pathol. 2025 Sep 29;26(10):e70160. doi: 10.1111/mpp.70160 (PMC12477439; doi:10.1111/mpp.70160)
Supplement: Supplementary file 22 — Table S3: List of primers used for this study. [file MPP-26-e70160-s012.docx]

**Supplemental Table 3. List of primers used in this study**

| **Name** | **Sequence(5'-3')** | **purpose** |
| --- | --- | --- |
| SlUBA1-F | CACCATGCTTCCTAGAAAGAGAC | SlUBA1 gene cloning |
| SlUBA1-R | GAATGCCAAATGTAGCAGAGG | SlUBA1 gene cloning |
| SlUBA2-F | CACCATGCTTCCTGTGAAGAGGTCA | SlUBA2 gene cloning |
| SlUBA2-R | CTACCTGAAATAAATAGAGACTTGAGGGATAT | SlUBA2 gene cloning |
| SlUBA-chimeric-F | TGAGCTGGACTGTTTGGGACAGGTGG | Construct UFD-domain chimeric SlUBA1 and SlUBA2 |
| SlUBA-chimeric-R | CCACCTGTCCCAAACAGTCCAGCTCA | Construct UFD-domain chimeric SlUBA1 and SlUBA2 |
| SlUBA1or2-UFD-F | CACCATGGACCTGAGCTGGACTGT | FOR Y2H |
| SlUBA1-VIGS-F | CACCAGAACTAATCCAATGGCTTG | SlUBA1, NbUBA1a/b VIGS |
| SlUBA1-VIGS-R | GTCTTCGTCGTTGTCGTCATC | SlUBA1, NbUBA1a/b VIGS |
| SlUBA2-VIGS-F | CACCAAGTCTGTTACTTTGCACGAT | SlUBA2, NbUBA2a/b VIGS |
| SlUBA2-VIGS-R | TCTGTAAATACAACGGCCTGA | SlUBA2, NbUBA2a/b VIGS |
| SlUBA-VIGS-F | CACCGCGGTAGTGTTGGTGAACGGTC | Sl/NbUBA1/2 VIGS |
| SlUBA-VIGS-R | TCCTTCATCGTGCAAAGTAACAGACTT | Sl/NbUBA1/2 VIGS |
| SlEF1α qRT-f | TCCAAAGATGGTCAGACCCGTGAA | RT-PCR control, tomato EF1a gene |
| SlEF1α qRT-r | ATACCTAGCCTTGGAGTACTTGGG | RT-PCR control, tomato EF1a gene |
| SlUbe1ch6-RT-F | TCGTGTGTGGATGATGAAAGG | SlUBA1 RT-PCR |
| SlUbe1ch6-RT-R | CGAGGGTAAAGGAATAAGGTCT | SlUBA1 RT-PCR |
| SlUbe1ch9-RT-F | ACAGAGGAGGATGTTGGGAAGA | SlUBA2 RT-PCR |
| SlUbech9-RT-R | TTTGATGAAAGCAATAGGAGGC | SlUBA2 RT-PCR |
| SlUBA1-XhoI-F | CACCTCGAGATGCTTCCTAGAAAGAGACCGG | BiFC vector construction |
| SlUBA1-smaI-R | CCCGGGACGGAAGTATACAGACACCAG | BiFC vector construction |
| SlUBA2-XhoI-F | CACCTCGAGATGCTTCCTGTGAAGAGGTC | BiFC vector construction |
| SlUBA2-smaI-R | CCCGGGCCTGAAATAAATAGAGACTTG | BiFC vector construction |
| slUbc12-KpnI-XhoI-F | GGTACCCTCGAGATGGCTTCAAAGAGGATTCAG | BiFC vector construction |
| slUbc12-PstI-R | GGGCTGCAGACCCATTGCGTATTTCTGGG | BiFC vector construction |
| slUbc32-BiFC-F | CACCCTCGAGATGGCGGAAGACAAGTATAATC | BiFC vector construction |
| slUbc32-BiFC-R | CCCGGGCGATTCATCCATAAAGACAGCAC | BiFC vector construction |
| slUbc33-BiFC-F | CACCCTCGAGATGGCAGAAAAAGCATGTGTAAAG | BiFC vector construction |
| slUbc33-BiFC-R | CCCGGGAAGCTGAAGCAGAGGCAGGGCC | BiFC vector construction |
| slUbc34-BiFC-F | CACCCTCGAGATGGCAGAAAAGGCATGTG | BiFC vector construction |
| slUbc34-BiFC-R | CCCGGGAAGCTGAAGTAGCGGCAGAG | BiFC vector construction |
| SlUBA2-F | CACCATGCTTCCTGTGAAGAGGTCA | to clone part A of SlUBA2 CDS |
| Sluba2-Q1009K-R | CAGACCTTTGTTTTTAAGCCACTGAAGAAGC | to clone part A of SlUBA2 CDS |
| Sluba2-Q1009K-F | AAAAACAAAGGTCTGAAT | to clone part B of SlUBA2 CDS |
| SlUBA2-R | CTACCTGAAATAAATAGAGACTTGAGGGATAT | to clone part B of SlUBA2 CDS |
| SlUBA1-XhoI-F | CACCCTCGAGATCTTCTCGTTCAATCGCAGTTT | Topo cloning for SlUBA1, then construction of pGWB5-SlUBA1 for SlUBA1-GFP expression |
| SlUBA1-SmaI-R | CCCGGGACGGAAGTATACAGACACCAGAG | Topo cloning for SlUBA1, then construction of pGWB5-SlUBA1 for SlUBA1-GFP expression |
| SlUBA2-XhoI-F | CACCCTCGAGCAAAGCATCTCTCATACAAACCC | Topo cloning for SlUBA1, then construction of pGWB5-SlUBA2 for SlUBA2-GFP expression |
| SlUBA2-SmaI-R | CCCGGGCCTGAAATAAATAGAGACTTGAGG | Topo cloning for SlUBA1, then construction of pGWB5-SlUBA2 for SlUBA2-GFP expression |
| SlUBA1-SmaI-F | CACCCCCGGGATGCTTCCTAGAAAGAGACCGGCA | for SlUBA1-HA expressed in plants |
| SlUBA1-SalI-R | GTCGACACGGAAGTATACAGACACCAGAGG | for SlUBA1-HA expressed in plants |
| SlUBA2-KpnI-F | CACCGGTACCATGCTTCCTGTGAAGAGGTCA | for SlUBA2-HA expressed in plants |
| SlUBA2-SalI-R | GTCGACCCTGAAATAAATAGAGACTTGAGG | for SlUBA2-HA expressed in plants |
| GFP.KpnI.F | CACCGGTACCATGGTGAGCAAGGGCGA | for GFP-FLAG expressed in plants |
| GFP.XbaI.R | CCTTCTAGACTTGTACAGCTCGTCCATGC | for GFP-FLAG expressed in plants |
| SlUBC12.KpnI.F | CACCGGTACCATGGCTTCAAAGAGGATTCAG | for SlUBC12-FLAG expressed in plants |
| SlUBC12.XbaI.R | TCTAGAACCCATTGCGTATTTCTG | for SlUBC12-FLAG expressed in plants |
| SlUBC32.KpnI.F | CACCGGTACCATGGCGGAAGACAAGTATAA | for SlUBC32-FLAG expressed in plants |
| SlUBC32.XbaI.R | TCTAGACGATTCATCCATAAAGACAGC | for SlUBC32-FLAG expressed in plants |
| SlUBC33.KpnI.F | CACCGGTACCATGGCAGAAAAAGCATGTG | for SlUBC33-FLAG expressed in plants |
| SlUBC33.BamHI.R | GGATCCAAGCTGAAGCAGAGGCA | for SlUBC33-FLAG expressed in plants |
| SlUBC34.KpnI.F | CACCGGTACCATGGCAGAAAAGGCATGTG | for SlUBC34-FLAG expressed in plants |
| SlUBC34.BamHI.R | GGATCCAAGCTGAAGTAGCGGCAGA | for SlUBC34-FLAG expressed in plants |
| SlUBA1-GST-F | CACCCTTCCTAGAAAGAGACCGGCAGAA | for GST-SlUBA1 expressed in E.coli |
| SlUBA1-GST-R | TCAACGGAAGTATACAGACAC | for GST-SlUBA1 expressed in E.coli |
| SlUBA2-GST-F | CACCCTTCCTGTGAAGAGGTCATCG | for GST-SlUBA2, SlUBA2Q1009A/K expressed in E.coli |
| SlUBA2-GST-R | CTACCTGAAATAAATAGAGACTTG | for GST-SlUBA2, SlUBA2Q1009A/K expressed in E.coli |
| SlUbc7-GW-F1 | CACCATGGCTTCAACTTCTCCTTC | SlUBC7 and SlUBC7deleta13aa gateway cloning in pDEST15 vector |
| SlUbc7-GW-R1 | TTACATCATTTCTTGAGACCG | SlUBC7 and SlUBC7deleta13aa gateway cloning in pDEST15 vector |
| SlUBC7-delta F2 | CACCATGGCTTCAACTTCTCCTTC | SlUBC7deleta13aa gateway cloning in pDEST15 vector |
| SlUBC7-delta R2 | AAGGATTGAGATGCAAACCTTTCCA | SlUBC7deleta13aa gateway cloning in pDEST15 vector |
| SlUbc12-GW-F | CACCGCTTCAAAGAGGATTCAGAAGG | SlUBC12 and SlUBC12+13aa gateway cloning in pDEST15 vector |
| Sl12+13aa-R1 | ATCACCAGGGGGATGTAGGATATCAAGACA | SlUBC12+13aa gateway cloning in pDEST15 vector |
| Sl12+13aa-F2 | CATCCCCCTGGTGATGATCCAAATGGCTAT | SlUBC12+13aa gateway cloning in pDEST15 vector |
| Sl12+13aa-F3 | GATCCAAATGGCTATGAGCTAGCTAAGGAACAATGGAGC | SlUBC12+13aa gateway cloning in pDEST15 vector |
| Sl12+13aa-R2 | TCAACCCATTGCGTATTT | SlUBC12 and SlUBC12+13aa gateway cloning in pDEST15 vector |
| Sl-UBC1-EcoRI-F | CGGAATTCATGTCGACTCCAGCT | Sl-*UBC1* in the pGEX-4T-1 vector |
| Sl-UBC1-XhoI-R | AACTCGAGTCAGTCAGCAGTCCA | Sl-*UBC1* in the pGEX-4T-1 vector |
| Sl-UBC3-EcoRI-F | AGGAATTCATGTCGACACCGGCGAAG | Sl-*UBC3* in the pGEX-4T-1 vector |
| Sl-UBC3-XhoI-R | CGCTCGAGTCAGTCTGCTGTCCAGCTT | Sl-*UBC3* in the pGEX-4T-1 vector |
| Sl-UBC5-EcoRI-F | TAGGATCCATGTCTTCTCCAAGCAAACG | Sl-*UBC5* in the pGEX-4T-1 vector |
| Sl-UBC5-XhoI-R | TGCTCGAGTCATGGATCAACAGGGCCT | Sl-*UBC5* in the pGEX-4T-1 vector |
| Sl-UBC6-EcoRI-F | CAGAATTCATGTCTTCCCCTAGCAAACG | Sl-*UBC6* in the pGEX-4T-1 vector |
| Sl-UBC6-XhoI-R | TGCTCGAGTTAGGGATCTGCTTTTCCAG | Sl-*UBC6* in the pGEX-4T-1 vector |
| Sl-UBC8-EcoRI-F | ACGGATCCATGGCATCCAAGCGGATTC | Sl-*UBC8* in the pGEX-4T-1 vector |
| Sl-UBC8-XhoI-R | CGCTCGAGCTATCCCATGGCAAATTTTTG | Sl-*UBC8* in the pGEX-4T-1 vector |
| Sl-UBC9-GW-F | CACCATGGCATCCAAGAGGATTCT | Sl-*UBC9* ORF gateway cloning in pDEST15 vector |
| Sl-UBC9-GW-R | CTAACCCATTGCATACTTTTG | Sl-*UBC9* ORF gateway cloning in pDEST15 vector |
| Sl-UBC10-ORF-F | CACCATGGCTTCGAAACGAATATTGAA | Sl-*UBC10* ORF gateway cloning in pDEST15 vector |
| Sl-UBC10-ORF-R | TTAACCCATGGCATACTTCTG | Sl-*UBC10* ORF gateway cloning in pDEST15 vector |
| Sl-UBC11-GW-F | CACCATGGCATCAAGGAGAATTCAA | Sl-*UBC11* ORF gateway cloning in pDEST15 vector |
| Sl-UBC11-GW-R | TCAATTCATAGCATATTTTTGGG | Sl-*UBC11* ORF gateway cloning in pDEST15 vector |
| Sl-UBC13-ORF-F | CGGAATTCATGGCTAACAGC | Sl-*UBC13* in the pGEX-4T-1 vector |
| Sl-UBC13-ORF-R | CCGCTCGAGTCATGCACCACTAG | Sl-*UBC13* in the pGEX-4T-1 vector |
| Sl-UBC13-2-ORF-F | CGGAATTCATGGCTAACAGC | Sl-*UBC13-2* in the pGEX-4T-1 vector |
| Sl-UBC13-2-ORF-R | CCGCTCGAGTCATGCACCACTAG | Sl-*UBC13-2* in the pGEX-4T-1 vector |
| Sl-UBC15-EcoRI-F | CGGAATTCATGTCTTCTCCAAGC | Sl-*UBC15* in the pGEX-4T-1 vector |
| Sl-UBC15-XhoI-R | AACTCGAGTCAAGGATCAGCATG | Sl-*UBC15* in the pGEX-4T-1 vector |
| Sl-UBC16-BamHI-F | GAGGATCCATGACTAGTGCTTCTGCTTC | Sl-*UBC16* in the pGEX-4T-1 vector |
| Sl-UBC16-XhoI-R | CACTCGAGCTACACTTTATCGTCATGGAA | Sl-*UBC16* in the pGEX-4T-1 vector |
| Sl-UBC17-BamHI-F | AAGGATCCATGTCGGCCTCCTCTGCC | Sl-*UBC17* in the pGEX-4T-1 vector |
| Sl-UBC17-XhoI-R | TGCTCGAGTCACACCTTATCATCATGGAA | Sl-*UBC17* in the pGEX-4T-1 vector |
| Sl-UBC20-BamHI-F | GTGGATCCATGGCGACAATGAACAGTGG | Sl-*UBC20* in the pGEX-4T-1 vector |
| Sl-UBC20-XhoI-R | CGCTCGAGCTACACACTAGGCTTGTATAG | Sl-*UBC20* in the pGEX-4T-1 vector |
| Sl-UBC22-EcoRI-F | CGGAATTCATGGCAACTAATGAA | Sl-*UBC22* in the pGEX-4T-1 vector |
| Sl-UBC22-XhoI-R | CGCTCGAGTTATAATCTCTTCAA | Sl-*UBC22* in the pGEX-4T-1 vector |
| Sl-UBC27-BamHI-F | GAGGATCCATGGTGGACTTGGCTAGGG | Sl-*UBC27* in the pGEX-4T-1 vector |
| Sl-UBC27-XhoI-R | CGCTCGAGTTAGCTGGACAACAGCTTTTC | Sl-*UBC27* in the pGEX-4T-1 vector |
| Sl-UBC28-ORF-F | CACCATGGCTTCTAAGCGGATATTG | Sl-*UBC28* ORF gateway cloning in pDEST15 vector |
| Sl-UBC28-ORF-R | TTAACCCATAGCATACTTCTG | Sl-*UBC28* ORF gateway cloning in pDEST15 vector |
| Sl-UBC29-GW-F | CACCATGGCATCCAGGAGAATTCA | Sl-*UBC29* ORF gateway cloning in pDEST15 vector |
| Sl-UBC29-GW-R | TCAGTTCATGGCATACTTTTG | Sl-*UBC29* ORF gateway cloning in pDEST15 vector |
| Sl-UBC30-GW-F | CACCATGGCTTCCAAGCGGATCT | Sl-*UBC30* ORF gateway cloning in pDEST15 vector |
| Sl-UBC30-GW-R | TTAGCCCATGGCATACTTCT | Sl-*UBC30* ORF gateway cloning in pDEST15 vector |
| Sl-UBC31-EcoRI-F | TAGAATTCATGGCTTCGAAACGGATATT | Sl-*UBC31* in the pGEX-4T-1 vector |
| Sl-UBC31-XhoI-R | TTCTCGAGTTAACCCATTGCATATTTCTGG | Sl-*UBC31* in the pGEX-4T-1 vector |
| Sl-UBC32-BamHI-F | GTGGATCCATGGCGGAAGACAAGTATAAT | Sl-*UBC32* in the pGEX-4T-1 vector |
| Sl-UBC32-XhoI-R | CGCTCGAGTTACGATTCATCCATAAAGACA | Sl-*UBC32* in the pGEX-4T-1 vector |
| Sl-UBC35-ORF-F | CACCATGGCTTCAGCTTCTCCTTC | Sl-*UBC35* ORF gateway cloning in pDEST15 vector |
| Sl-UBC35-ORF-R | TTACGTCATTTCTTGGGACCG | Sl-*UBC35* ORF gateway cloning in pDEST15 vector |
| Sl-UBC38new-GW-F | CACCATGGCGTCCAAGCGGATTCT | Sl-*UBC38* ORF gateway cloning in pDEST15 vector |
| Sl-UBC38-GW-R | CTAACCCATGGCGTACTTTT | Sl-*UBC38* ORF gateway cloning in pDEST15 vector |
| Sl-UBC39-EcoRI-F | AGGAATTCATGGCGTCGAAGCGCATAT | Sl-*UBC39* in the pGEX-4T-1 vector |
| Sl-UBC39-XhoI-R | TGCTCGAGTTATCCCATCGCATATTTTTGA | Sl-*UBC39* in the pGEX-4T-1 vector |
| Sl-UBC40-EcoRI-F | TTGAATTCATGGCGTCGAAGAGGATATT | Sl-*UBC40* in the pGEX-4T-1 vector |
| Sl-UBC40-XhoI-R | AACTCGAGTCATCCCATTGCATATTTCTGA | Sl-*UBC40* in the pGEX-4T-1 vector |
| Sl-UBC41-EcoRI-F | CGGAATTCATGTCGACGCCGGCT | Sl-*UBC41* in the pGEX-4T-1 vector |
| Sl-UBC41-XhoI-R | AACTCGAGTCAGTCCGCCGTCCA | Sl-*UBC41* in the pGEX-4T-1 vector |
| Sl-UBC12-F-GW | CACCATGGCTTCAAAGAGGATTCAG | Sl-*UBC12* ORF gateway cloning in pDEST17 vector |
| Sl-UBC12-R | CGCTCGAGTCAACCCATTGCGTATTTCT | Sl-*UBC12* ORF gateway cloning in pDEST17 vector |
| AtUBA1-GST-F | CACCATGCTTCACAAGCGAGCTAGTGAAGC | AtUBA1 ORF gateway cloning in pDEST15 vector |
| AtUBA1-GST-R | TCACCTGAAGTAGATAGAGACGAG | AtUBA1 ORF gateway cloning in pDEST15 vector |
| AtUBA2-GST-F | CACCATGGAACCATTCGTTGTTAAGGAG | AtUBA2 ORF gateway cloning in pDEST15 vector |
| AtUBA2-GST-R | TCAGGCGAAGTAGACTGATACGAG | AtUBA2 ORF gateway cloning in pDEST15 vector |
| AtUBC32-XhoI-F | CACCCTCGAGATGGCGGATGAGAGGTATAATC | Clone AtUBC32 for BiFC and Protein expression |
| AtUBC32-SmaI-R | CCCGGGAGACTGATCATCCATAAACCCAG | Clone AtUBC32 for BiFC and Protein expression |
| AtUBC33-XhoI-F | CACCCTCGAGATTTAGATGGCAGAAAAAGCTTG | Clone AtUBC33 for BiFC and Protein expression |
| AtUBC33-SmaI-R | CCCGGGCAGCTGAAGCAAAGGCAACG | Clone AtUBC33 for BiFC and Protein expression |
| AtUBC34-XhoI-F | CACCTCTAGAACGTAGATGGCAGAAAAGGCC | Clone AtUBC34 for BiFC and Protein expression |
| AtUBC34-SmaI-R | CCCGGGCAGTTGAAGCAGAGGCAACG | Clone AtUBC34 for BiFC and Protein expression |
| SlIAA17-RT-F | ATGTACAAGAGTTACCAACAAC | Forward semi-quantitative (sq)RT-PCR primer to test SlIAA17 |
| SlIAA17-RT-R | TCAGCTCCTGTTCTTGCATT | Reverse sqRT-PCR primer to test SlIAA17 |
| SlJAZ1-RT-F | GGCTAGTACTAGCAAGGG | Forward sqRT-PCR primer to test SlJAZ1 |
| SlJAZ1-RT-F | CTAGTATTGCTCAGTTTTCA | Reverse sqRT-PCR primer to test SlJAZ1 |
| NbEF1a-RT-F | AGCCTGGTATGGTTGTGACTTTTG | RT-PCR control, tobacco EF1a gene |
| NbEF1a-RT-R | CATGGGCTTGGTGGGAATC | RT-PCR control, tobacco EF1a gene |
| NbUBA2-RT-F | TTTATGGCCGTGAAACTATGCG | Forward sqRT-PCR primer to test NbUBA2 |
| NbUBA2-RT-R | ATTCTTCCCAACATCCTCCTCT | Reverse sqRT-PCR primer to test NbUBA2 |
| NbIAA17.1-RT-F | ATGTACAAAAGTTACCAGC | Forward sqRT-PCR primer to test NbIAA17.1 |
| NbIAA17.1-RT-R | TCAAATCCTGCGCTTG | Reverse sqRT-PCR primer to test NbIAA17.1 |
| NbIAA17.2-RT-F | CTGATGCTTTGGCCAAGATG | Forward sqRT-PCR primer to test NbIAA17.2 |
| NbIAA17.2-RT-R | TCAAACCCTGCGCTTGCATT | Reverse sqRT-PCR primer to test NbIAA17.2 |
| NbJAZ1.1-RT-F | GATAAAGCTAAGGAAATCAT | Forward sqRT-PCR primer to test NbJAZ1.1 |
| NbJAZ1.1-RT-R | CTAGAATTGCTCAGCTTTC | Reverse sqRT-PCR primer to test NbJAZ1.1 |
| NbJAZ1.2-RT-F | GATAAAGCTGAGGAAATC | Forward sqRT-PCR primer to test NbJAZ1.2 |
| NbJAZ1.2-RT-R | CTAGAATTGCTCAGCTTTC | Reverse sqRT-PCR primer to test NbJAZ1.2 |
